# Iodoarene-catalyzed cyclizations of *N*-propargylamides and β-amidoketones: synthesis of 2-oxazolines

**DOI:** 10.3762/bjoc.13.177

**Published:** 2017-08-31

**Authors:** Somaia Kamouka, Wesley J Moran

**Affiliations:** 1 Department of Chemistry, University of Huddersfield, Queensgate, Huddersfield HD1 3DH, UK. Tel: +44-1484-473741

**Keywords:** amides, catalysis, cyclization, hypervalent iodine, isoxazolines

## Abstract

Two complementary iodoarene-catalyzed methods for the preparation of 2-oxazolines are presented. The first involves the cyclization of *N*-propargylamides and the second involves the cyclization of β-amidoketones. These are proposed to proceed through different mechanisms and have different substrate scopes.

## Introduction

Hypervalent iodine reagents are of increasing importance in organic synthesis owing to their ease-of-use, low toxicity and relative low cost. Importantly, a wide range of useful reactivity has been uncovered with these compounds and many reviews are available [1–5]. One major advance in recent years is the emergence of conditions to effect catalytic processes with sub-stoichiometric quantities of iodine compound in the presence of an oxidant [6–11].

**Scheme 1:**
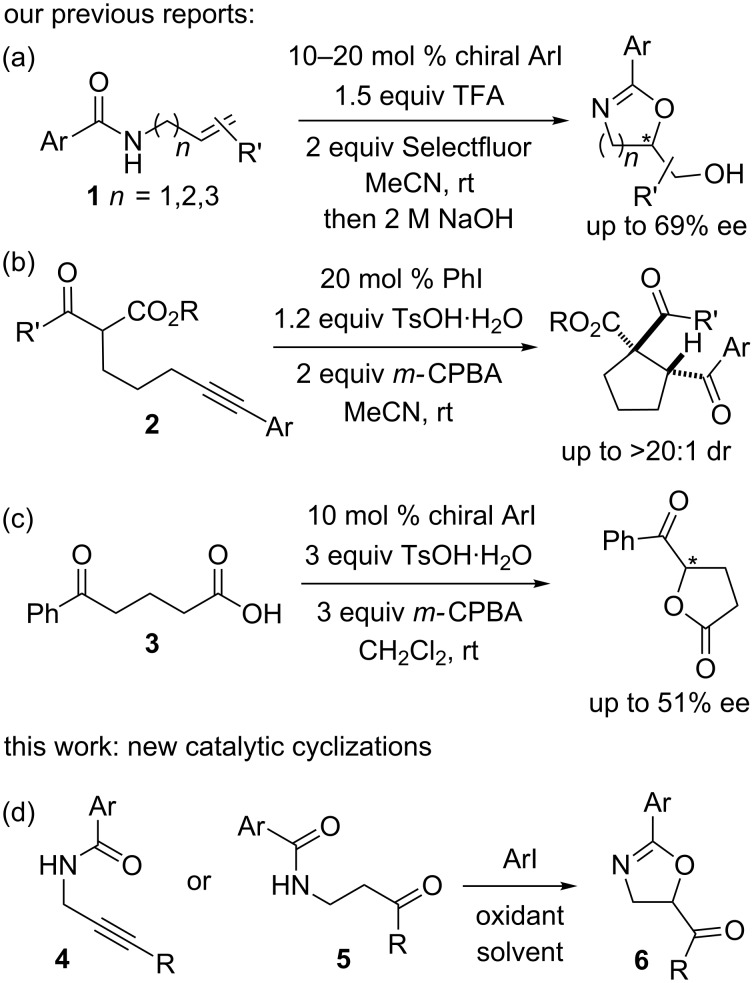
Our previous and current iodoarene-catalyzed cyclizations.

In this regard, we have reported the use of iodoarenes as precatalysts in the cyclizations of *N*-alkenylamides **1** [12], δ-alkynyl β-ketoesters **2** [13] and 5-oxo-5-phenylpentanoic acid (**3**, Scheme 1a–c) [14]. These three cyclizations exemplify three different proposed reaction pathways, i.e., iodine(III) activation of alkenes, alkynes and ketones. These cyclizations can be rendered enantioselective by the generation of non-racemic chiral iodine(III) species from chiral iodoarenes [15–17].

**Figure 1:**
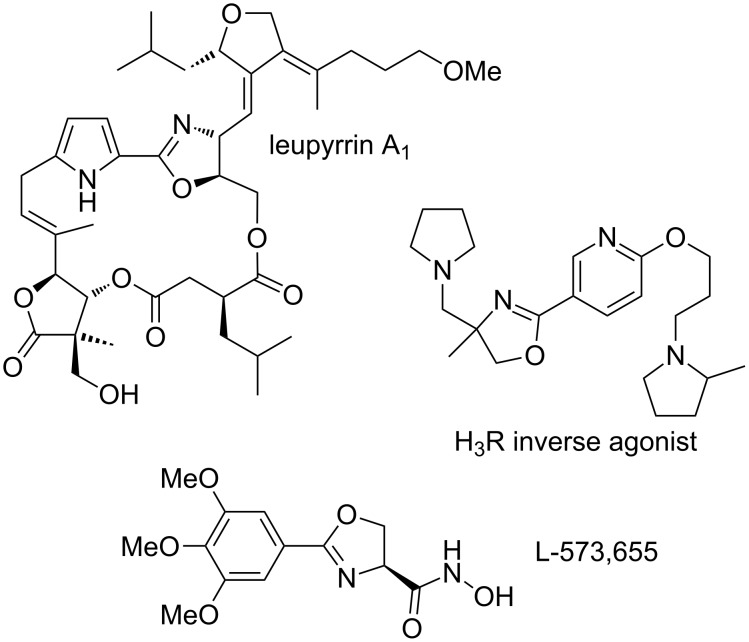
Examples of biologically-active compounds containing an oxazoline ring.

We wished to develop this cyclization methodology further and investigate the cyclization of the amide functional group on to alkynes and methylene groups adjacent to ketones in analogy to our previous work (Scheme 1d). This would provide two complementary routes to substituted 2-oxazolines, which are valuable heterocycles found in ligand scaffolds, natural products such as the leupyrrins [18–19], and potential pharmaceuticals (Figure 1) [20–22]. Traditional routes to this heterocycle include the dehydration of amino alcohols with carboxylic acids, however, this process typically requires forcing conditions such as heating at over 200 °C [23]. Several related processes that operate under milder conditions have been reported in recent years but they suffer from issues such as limited substrate scope or the requirement for expensive reagents or transition metal salts [24–29]. Saito and co-workers have reported the cyclization of propargylamides to form oxazoles rather than oxazolines under stoichiometric and, more recently, catalytic hypervalent iodine conditions [30–31]. Various other methods for the cyclization of unsaturated amides have been reported [32–36]. Gao and co-workers described the I_2_-catalyzed cyclization of β-acylaminoketones using *tert*-butyl hydroperoxide as the oxidant; notably, adding DBU led to oxazole formation whereas adding K_2_CO_3_ generated oxazolines [37–38]. Our proposed new approaches to oxazoline formation utilise readily available starting materials and operate under mild organocatalytic conditions.

## Results and Discussion

We initiated our study with readily available alkyne **4a** and subjected it to reaction conditions similar to those we have previously reported (Table 1). By using 2-iodoanisole as precatalyst in the presence of *p*-toluenesulfonic acid and *m*-chloroperbenzoic acid in acetonitrile, **4a** cyclized to **6a** in 92% yield as determined by ^1^H NMR analysis of the crude reaction mixture (Table 1, entry 1). In line with our previous findings with *N*-alkenylamides, the use of iodobenzene in place of 2-iodoanisole provided a diminished yield of **6a** (Table 1, entry 2) [12]. The iodoarene was found to be essential for the conversion of the starting material, as its absence led to complete return of **4a** (Table 1, entry 3). Using Oxone as oxidant led to essentially no conversion of **4a** (Table 1, entry 4). Changing the acid to TFA or changing the solvent to CH_2_Cl_2_ led to significantly lower yields of **6a** (Table 1, entries 5 and 6). Reducing the number of equivalents of oxidant and/or acid also led to lower yields (Table 1, entries 7–9). Importantly, formation of the six-membered ring was not observed under any conditions studied.

**Table 1:** Investigation of reaction conditions.

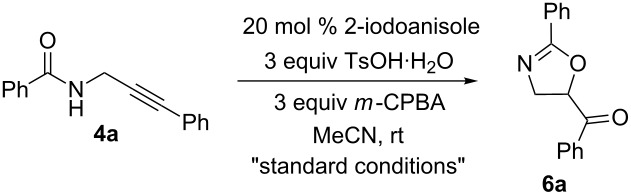		

Entry	Deviations from the standard conditions	Yield [%]^a^

1	none	92 (73)^b^
2	iodobenzene instead of 2-iodoanisole	60
3	no 2-iodoanisole	0
4	Oxone instead of *m*-CPBA	<5
5	TFA instead of TsOH·H_2_O	19
6	CH_2_Cl_2_ instead of MeCN	37
7	2 equiv *m*-CPBA and 2 equiv TsOH·H_2_O	54
8	1 equiv *m*-CPBA and 1 equiv TsOH·H_2_O	44
9	3 equiv *m*-CPBA and 1 equiv TsOH·H_2_O	41

^a^Determined by NMR analysis by comparison to a known quantity of 1,3,5-trimethoxybenzene. ^b^Yield of isolated compound.

**Scheme 2:**
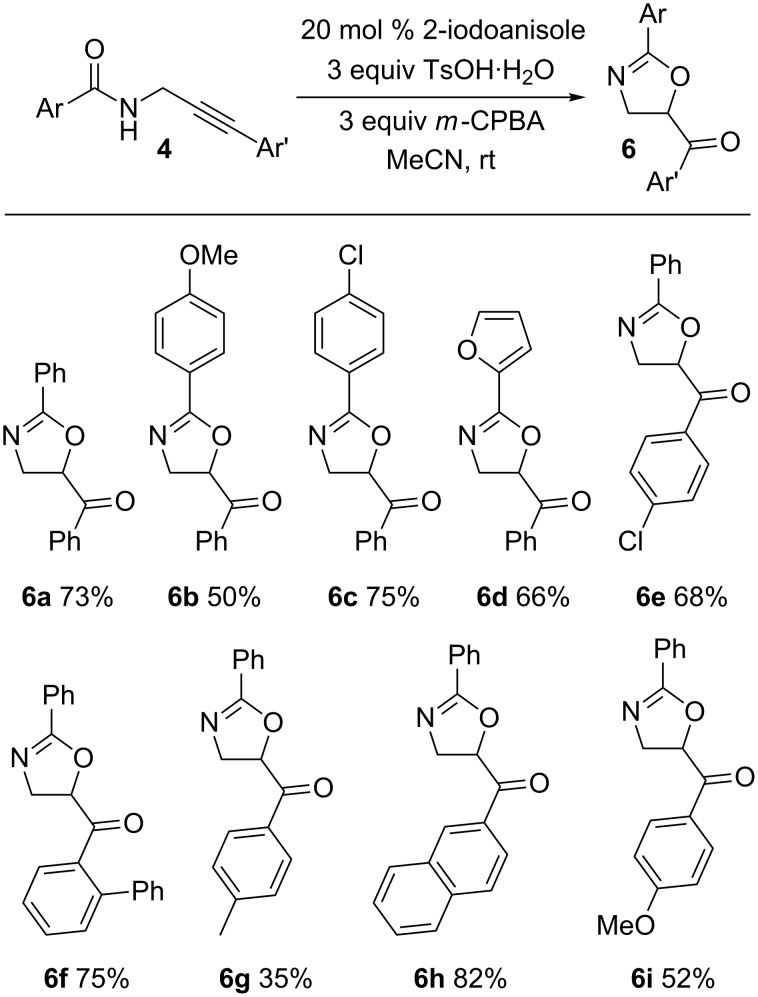
2-Iodoanisole-catalyzed cyclization of *N*-propargylamides.

With the optimal cyclization conditions in hand, the scope of this cyclization process was investigated for a range of propargylamides **4** which are readily accessible from propargylamine by amidation and Sonogoshira coupling (Scheme 2) [39]. The cyclization was successful in all cases studied with various arylamide and alkyne substituents. All functional groups were well tolerated apart from an alkyne terminated with an alkyl substituted arene which led to a diminished yield of product, i.e., **6g**.

**Scheme 3:**
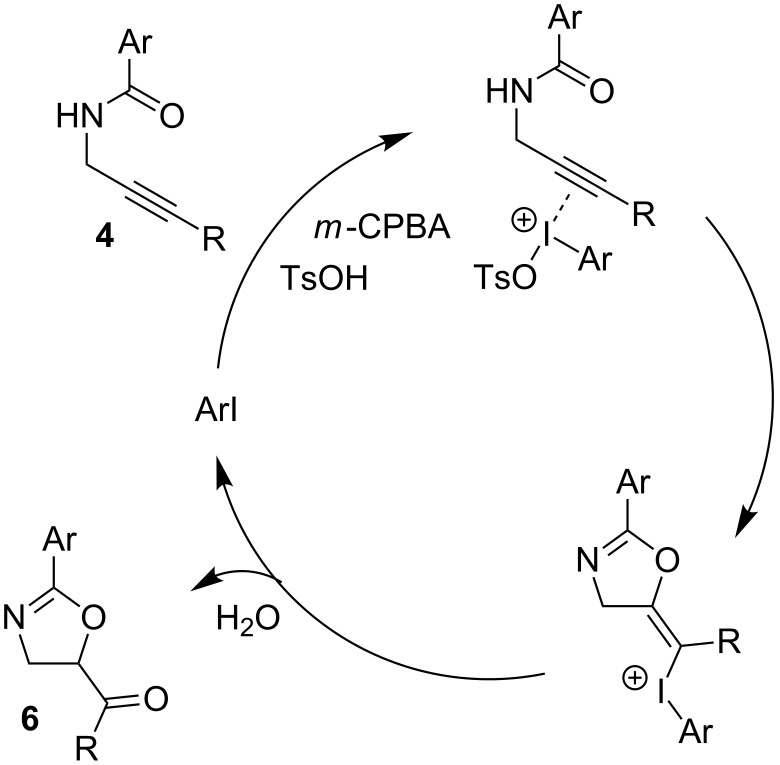
Postulated mechanism for *N*-propargylamide cyclization.

The mechanism of this cyclization is proposed to proceed though activation of the alkyne by an in situ generated iodine(III) species followed by intramolecular attack by the amide (Scheme 3). Subsequent addition of water leads to the loss of the iodoarene and tautomerization of the resulting enol generates the ketone **6**.

**Scheme 4:**
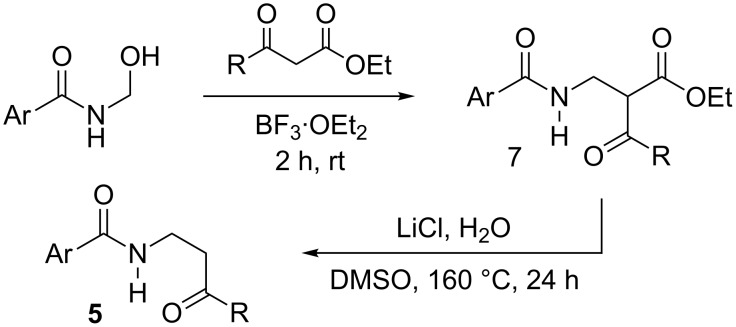
Synthesis of β-amidoketones **5**.

With these results in hand, we envisaged an alternative approach to 2-oxazoline formation through the iodoarene-catalyzed cyclization of β-amidoketones **5**. These are readily prepared by alkylation of the corresponding β-ketoester followed by decarboxylation (Scheme 4) [40–41].

**Scheme 5:**
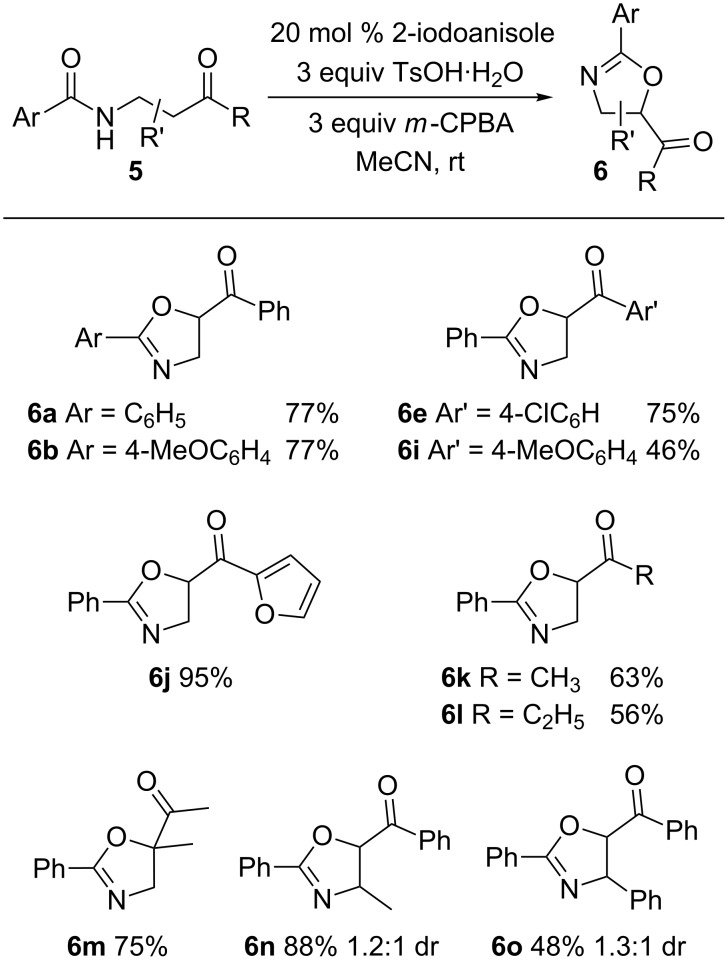
2-Iodoanisole-catalyzed cyclization of β-amidoketones **5**.

The cyclization of β-amidoketones **5** was successful with the same conditions as propargylamides **4** (Scheme 5). In line with the results for the propargylamides, iodobenzene was an inferior pre-catalyst to 2-iodoanisole and other oxidants, acids and solvents led to lower yields of **6**.

The scope of this cyclization process was explored and different aromatic amide and ketone groups were well tolerated. Alkylketone substrates were also successfully converted to 2-oxazolines. Installation of substituents on the tether led to facile formation of product **6m** containing a quaternary carbon and compounds **6n** and **6o** but without any observed diastereoselectivity. Interestingly, the selectivity for **6o** could be improved to 5:1 by substituting *p*-toluenesulfonic acid with trifluoroacetic acid albeit with a loss of yield.

**Scheme 6:**
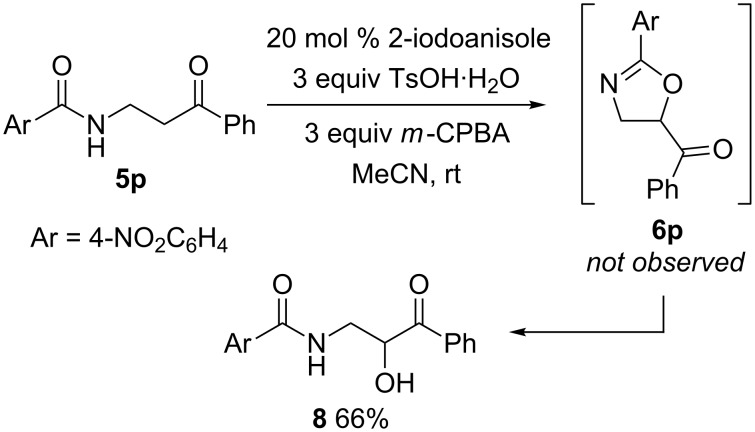
In situ oxazoline ring hydrolysis.

When the *p*-nitrophenylamide **5p** was subjected to the reaction conditions, the expected oxazoline **6p** was not observed (Scheme 6). Instead, alcohol **8** was isolated in 66% yield. Presumably, **6p** is formed under the reaction conditions but the oxazoline ring is readily hydrolysed due to the influence of the electron-withdrawing nitro group on the aromatic ring.

**Scheme 7:**
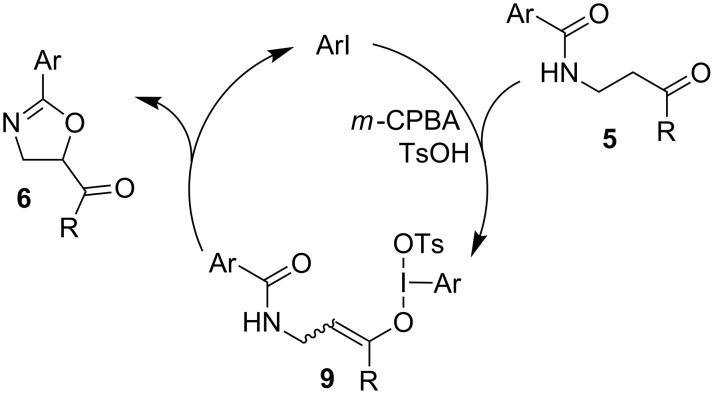
Postulated mechanism for cyclization of β-amidoketones.

The mechanism of this cyclization is proposed to proceed through the formation of iodine(III)-enolate **9** followed by intramolecular attack by the amide and release of the iodoarene (Scheme 7).

These two cyclizations are complementary, however, the reaction with β-amidoketones exhibits a superior substrate scope. In addition, the use of a chiral iodoarene should lead to enantioselective cyclizations of β-amidoketones; this is not possible with the propargylamides.

## Conclusion

Two simple and convenient iodoarene-catalyzed methods to prepare substituted 2-oxazolines are reported. One involves the cyclization of propargylamides and the second of β-amidoketones. These two complementary procedures are efficient and showcase the utility of hypervalent iodine in catalytic procedures. Studies concerning the elucidation of reaction mechanisms are ongoing and will be reported in due course.

## Experimental

**General procedure for 2-iodoanisole-catalyzed cyclization of *N*-propargylamide 4 or β-amidoketone 5:** Propargylamide **4** (1 equiv) or β-amidoketone **5** (1 equiv) was dissolved in acetonitrile (0.14 M) and 2-iodoanisole (0.2 equiv) was added, followed by *m*-CBPA (3 equiv) and *p*-TsOH·H_2_O (3 equiv). The mixture was stirred overnight at room temperature, then saturated aqueous Na_2_S_2_O_3_ solution and saturated aqueous NaHCO_3_ solution were added and the mixture extracted with CH_2_Cl_2_. The organic layers were combined and dried with MgSO_4_, filtered and concentrated under vacuum. The product was purified by flash chromatography (9:1 petroleum ether/EtOAc) to provide oxazoline **6**. See Supporting Information File 1 for full experimental details.

## Supporting Information

File 1 Full experimental details, characterization data and copies of NMR spectra.
