# Supplementary material for: Iodoarene-catalyzed cyclizations of N-propargylamides and β-amidoketones: synthesis of 2-oxazolines
Source: Beilstein J Org Chem. 2017 Aug 31;13:1823–7. doi: 10.3762/bjoc.13.177 (PMC5588552; doi:10.3762/bjoc.13.177)
Supplement: File 1 — Full experimental details, characterization data and copies of NMR spectra. [file Beilstein_J_Org_Chem-13-1823-s001.pdf]

Supporting Information

for

Iodoarene-catalyzed cyclizations of *N*-propargylamides and β-amidoketones: synthesis of 2-oxazolines

Somaia Kamouka and Wesley J. Moran*

Address: Department of Chemistry, University of Huddersfield, Queensgate, Huddersfield HD1 3DH, UK. Tel: +44-1484-473741

Email: Wesley J. Moran - w.j.moran@hud.ac.uk

*Corresponding author

Full experimental details, characterization data and copies of NMR spectra

Contents

Experimental procedures and characterization data S2

^{1}H NMR and ^{13}C NMR spectra for novel compounds S14

General: ^{1}H NMR spectra were recorded at 400 MHz in $CDCl_3$ unless otherwise stated. Chemical shifts are reported in ppm from tetramethylsilane with the solvent resonance as the internal standard ($CDCl_3$: 7.26 ppm). Data are reported as follows: chemical shift, integration, multiplicity (s = singlet, d = doublet, t = triplet, q = quartet, br = broad, m = multiplet), and coupling constants (Hz). ^{13}C NMR were recorded at 100 MHz in $CDCl_3$ unless otherwise stated with complete proton decoupling. Chemical shifts are reported in ppm from tetramethylsilane with the solvent as the internal standard ($CDCl_3$: 77.4 ppm). Mass spectrometry (*m*/*z*) was performed in ESI mode, with only molecular ions being reported. Infrared (IR) spectra ν_{max} are reported in cm^{-1}. Bands are characterized as broad (br), strong (s), medium (m) and weak (w). All purchased reagents were used as received without further purification. Petroleum ether refers to the fraction boiling at 40–60 °C. Characterization data for known compounds matched the literature values: **4a**,[1] **4b**,[1] **4d**,[2] **4e**,[3] **4g**,[3] **4h**,[2] **4i**,[3] **5a**,[4] **5i**,[3] **5k**,[5] **5n**,[6] **5o**.[7]

Preparation of 4-chloro-*N*-(3-phenylprop-2-yn-1-yl)benzamide, 4c: 4-Chlorobenzoyl chloride (0.23 mL, 1.8 mmol) was added dropwise to an ice cooled solution of 3-phenyl-2-propyn-1-amine·HCl (0.30 g, 1.8 mmol) and triethylamine (0.50 mL, 3.6 mmol) in CH_2Cl_2 (10 mL) and the mixture was stirred overnight at room temperature. The mixture was washed with water and extracted with CH_2Cl_2 (3 × 10 mL). The organic layers were dried over anhydrous $MgSO_4$ and the solvents were removed under vacuum to give **4c** as a white solid (0.48 g, 99% yield). Mp: 157-158 °C. IR: 3257 (br), 3073 (w), 1633 (s), 1545 (s), 1487 (s), 1299 (m), 1093 (m), 751 (s) cm^{-1}. ^{1}H NMR: δ 4.48 (2H, d, *J* = 5.1 Hz), 6.37 (1H, br), 7.28-7.35 (3H, m), 7.40-7.46 (4H, m), 7.76 (2H, d, *J* = 8.2 Hz). ^{13}C {^{1}H} NMR: δ 31.1, 84.3, 84.8, 122.7, 128.7 (2C), 128.9 (2C), 129.0, 129.3 (2C), 132.1 (2C), 132.6, 138.4, 166.4. HRMS (ESI-TOF) m/z: $[M+H]^+$ Cald for $C_{16}H_{13}ClNO$ 270.0680; Found 270.0680.

---

[1] Sinai, Á.; Vangel, D.; Gáti, T.; Bombicz, P.; Novák, Z. *Org. Lett.* **2015**, *17*, 4136.
[2] Hashmi, A. S. K.; Schuster, A. M.; Schmuck, M.; Rominger, F. *Eur. J. Org. Chem.* **2011**, 4595.
[3] Bukšnaitienė R.; Čikotienė, I. *Synlett* **2015**, *26*, 479.
[4] Rimoldi, I.; Cesarotti, E.; Zerla, D.; Molinari, F.; Albanese, D.; Castellano, C.; Gandolfi, R. *Tetrahedron: Asymm.* **2011**, *22*, 597.
[5] Zhao, Y.; Yim, W.-L.; Tan, C. K.; Yeung, Y.-Y. *Org. Lett.* **2011**, *13*, 4308.
[6] Lin, Y.-D.; Kao, J.-Q.; Chen, C.-T. *Org. Lett.* **2007**, *9*, 5195.
[7] Gao, W.-C.; Hu, F.; Huo, Y.-M.; Chang, H.-H.; Li, X.; Wei, W.-L. *Org. Lett.* **2015**, *17*, 3914.

Preparation of *N*-(3-([1,1'-biphenyl]-2-yl)prop-2-yn-1-yl)benzamide, 4f: *N*-(Prop-2-yn-1-yl)benzamide (0.70 g, 6.3 mmol) was added to a mixture of 2-iodobiphenyl (0.70 mL, 4.0 mmol), $PdCl_2(PPh_3)_2$ (0.62 g, 0.88 mmol) and Et_3N (1.8 mL, 13 mmol) in dry THF (10 mL) at room temperature under N_2. The mixture was left to stir for 5 min then CuI (0.08 g, 0.44 mmol) was added. After stirring overnight at room temperature the solvent was removed by rotary evaporation, and the crude product was purified by flash column chromatography (5:1 petroleum ether/EtOAc) to give **4f** as a light brown solid (0.48 g, 24% yield). Mp: 113-115 °C. IR: 3290 (br), 1630 (s), 1525(s), 1301 (m), 1077 (w), 690 (s) cm^{-1}. ^1H NMR: δ 4.35 (2H, d, *J* = 5.0 Hz), 6.10 (1H, br), 7.28-7.34 (2H, m), 7.35-7.41 (4H, m), 7.44 (2H, t, *J* = 7.5 Hz), 7.50-7.60 (4H, m), 7.72 (2H, d, *J* = 7.8 Hz). ^{13}C {^1H} NMR: δ 31.2, 84.0, 87.8, 121.2, 127.3 (2C), 127.4, 127.9, 128.3 (2C), 128.9 (2C), 129.1, 129.6 (2C), 129.8, 132.1, 133.2, 134.2, 140.9, 144.4, 167.3. HRMS (ESI-TOF) m/z: $[M+H]^+$ Cald for $C_{22}H_{18}NO$ 312.1383; Found 312.1392.

General procedure for the synthesis of 7: Preparation of ethyl 2-((4-methoxybenzamido)methyl)-3-oxo-3-phenylpropanoate, 7b. A mixture of *N*-(hydroxymethyl)-4-methoxybenzamide (2.0 g, 11 mmol, 1 equiv) and ethyl benzoylacetoacetate (1.9 mL, 11 mmol, 1 equiv) was cooled to 0 °C and $BF_3·OEt_2$ solution (2.7 mL, 22 mmol, 2 equiv) was added slowly with stirring. The reaction mixture was left to stir at room temperature for 2 h. The resulting mixture was added to a solution of sodium acetate (4 g) in water (8 mL) mixed well and allowed to separate. The aqueous layer was extracted twice with CH_2Cl_2 (10 mL). The organic layers were dried over anhydrous $MgSO_4$ and the solvents were removed under vacuum to provide **7b** as a yellow wax (3.7 g, 94%). IR: 3341 (br), 2979 (w), 2931 (w), 1731 (m), 1636 (m), 1499 (s) cm^{-1}. ^1H NMR: δ 1.15 (3H, t, *J* = 7.1 Hz), 3.80 (3H, s), 3.85-3.94 (1H, m), 4.04-4.19 (3H, m), 4.89 (1H, t, *J* = 6.7 Hz), 6.85-6.94 (3H, m), 7.48 (2H, t, *J* = 7.5 Hz), 7.58 (1H, t, *J* = 7.5 Hz), 7.71 (2H, d, *J* = 8.8 Hz),

8.09 (2H, d, *J* = 7.5 Hz). ^{13}C {^{1}H} NMR: δ 14.0, 30.9, 39.3, 53.6, 55.4, 61.8, 113.8 (2C), 126.3, 128.9 (2C), 129.0 (3C), 134.1, 135.8, 162.4, 167.6, 169.2, 194.9. HRMS (ESI-TOF) m/z: $[M+H]^+$ Cald for $C_{20}H_{22}NO_5$ 356.1492; Found 356.1498.

Ethyl 2-(benzamidomethyl)-3-(4-chlorophenyl)-3-oxopropanoate, 7e. Yield: 81% (3.1 g). Colorless oil. IR: 3342 (br), 3067 (w), 1729 (s), 1639 (s), 1525 (m), 1091 (s) cm^{-1}. ^{1}H NMR: δ 1.11 (3H, t, *J* = 7.1 Hz), 3.90 (1H, ddd, *J* = 14, 7.2, 6.4 Hz), 4.02 (1H, dd, *J* = 13, 6.6 Hz), 4.09 (2H, dq, *J* = 7.1, 2.5 Hz), 4.89 (1H, t, *J* = 6.7 Hz), 7.26-7.45 (6H, m), 7.72 (2H, t, *J* = 8.1 Hz), 7.99 (2H, d, *J* = 8.5 Hz). ^{13}C {^{1}H} NMR: δ 14.1, 39.3, 53.5, 62.0, 127.2 (2C), 128.7 (2C), 129.3 (2C), 130.5 (2C), 131.9, 133.9, 134.2, 140.7, 168.2, 168.9, 193.8. HRMS (ESI-TOF) m/z: $[M+H]^+$ Cald for $C_{19}H_{19}ClNO_4$ 360.0997; Found 360.0991.

Ethyl 2-(benzamidomethyl)-3-(furan-2-yl)-3-oxopropanoate, 7j. Yield: 46% (1.2 g). White solid. Mp: 110-112 °C. IR: 3364 (br), 3133 (w), 2979 (w), 1723 (m), 1639 (s), 1275 cm^{-1}. ^{1}H NMR δ 1.12 (3H, t, *J* = 7.1 Hz), 3.98 (1H, ddd, *J* = 14, 7.3, 6.4 Hz), 4.04 (1H, ddd, *J* = 14, 7.3, 6.4 Hz), 4.19 (2H, dq, *J* = 7.3, 1.4 Hz), 4.61 (1H, t, *J* = 6.4 Hz), 6.58 (1H, dd, *J* = 3.7, 1.6 Hz), 6.86 (1H, br), 7.38-7.46 (3H, m), 7.46-7.52 (1H, m), 7.63-7.66 (1H, m), 7.70-7.76 (2H, m). ^{13}C {^{1}H} NMR: *δ* 14.3, 38.9, 53.6, 62.2, 113.2, 120.2, 127.3 (2C), 128.9 (2C), 131.9, 134.3, 148.0, 151.9, 167.9, 168.9, 183.1. HRMS (ESI-TOF) m/z: $[M+H]^+$ Cald for $C_{17}H_{18}NO_5$ 316.1179; Found 316.1179.

Ethyl 2-(benzamidomethyl)-3-oxopentanoate, 7l. Yield: 55% (2.2 g). White wax. IR: 3363 (br), 2939 (w), 1736 (s), 1630 (m), 1524 (s), 717 (s) cm^{-1}. ^{1}H NMR: δ 0.98 (3H, t, *J* = 7.3 Hz), 1.17 (3H, t, *J* = 7.3 Hz), 2.46-2.67 (2H, m), 3.82 (2H, dt, *J* = 6.2, 2.0 Hz), 3.96 (1H, t, *J* = 6.3 Hz), 4.10 (2H, q, *J* = 7.1Hz), 7.13 (1H, br), 7.31 (2H, t, *J* = 7.6 Hz), 7.40 (1H, t, *J* = 7.3 Hz), 7.68 (2H, d, *J* = 8.1 Hz). ^{13}C {^{1}H} NMR: δ 7.7, 14.2, 36.3, 38.2, 57.4, 61.8, 127.2 (2C),

128.7 (2C), 131.7, 134.2, 167.8, 168.9, 205.6. HRMS (ESI-TOF) m/z: $[M+H]^+$ Cald for $C_{15}H_{20}NO_4$ 278.1387; Found 278.1391.

Ethyl 2-(benzamidomethyl)-2-methyl-3-oxopentanoate, 7m. Yield: 82% (3.0 g). Light yellow oil. IR: 3345 (br), 2985 (w), 1735 (s), 1645 (m), 1525 (m), 1242 (s) cm^{-1}. ^1H NMR: δ 1.06 (3H, t, *J* = 7.1 Hz), 1.28 (3H, s), 2.05 (3H, s), 3.63-3.77 (2H, m), 3.97-4.01 (2H, m), 6.99 (1H, br), 7.21 (2H, t, *J* = 6.3 Hz), 7.29 (1H, t, *J* = 7.6 Hz), 7.56 (2H, d, *J* = 7.4 Hz). ^{13}C {^1H} NMR: δ 13.8, 17.9, 26.2, 43.3, 60.3, 61.7, 126.9 (2C), 128.4 (2C), 134.2, 167.6, 171.7, 205.7. HRMS (ESI-TOF) m/z: $[M+H]^+$ Cald for $C_{15}H_{20}NO_4$ 278.1387; Found 278.1392.

Ethyl 2-((4-nitrobenzamido)methyl)-3-oxo-3-phenylpropanoate, 7p. Yield: 39% (1.33 g). White solid. Mp: 141-144 °C. IR: 3376 (m), 3064 (w), 1725 (s), 1666 (m), 1520 (s), 1198 (s) cm^{-1}. ^1H NMR: δ 1.19 (3H, t, *J* = 7.1 Hz), 3.94-4.03 (1H, m), 4.10-4.25 (3H, m), 4.83 (1H, t, *J* = 6.0), 7.05 (1H, br), 7.52 (2H, t, *J* = 8.1 Hz), 7.63 (1H, t, *J* = 8.1Hz), 7.90 (2H, d, *J* = 8.6 Hz), 8.06 (2H, d, *J* = 7.8 Hz), 8.26 (2H, d, *J* = 8.6 Hz). ^{13}C {^1H} NMR: δ 14.2, 39.4, 53.4, 62.4, 124.2 (2C), 128.6 (2C), 129.2 (2C), 129.3 (2C), 134.6, 135.8, 139.9, 150.0, 166.1, 169.2, 194.9. HRMS (ESI-TOF) m/z: $[M+H]^+$ Cald for $C_{19}H_{19}N_2O_6$ 371.1238; Found 371.1250.

General procedure for the synthesis of β-amidoketones 5: Preparation of 4-methoxy-*N*-(3-oxo-3-phenylpropyl)benzamide, 5b. A flask was charged with **7b** (1.67 g, 4.69 mmol, 1 equiv), LiCl (0.69 g, 16 mmol, 3.5 equiv), H_2O (2.6 mL) and DMSO (35 mL) at room temperature. The solution was stirred for 24 h at 160 °C. The resulting solution was cooled to room temperature and diluted with water (20 mL). The mixture was extracted with diethyl ether (20 mL × 3). The combined organic extracts were dried over $MgSO_4$, filtered and concentrated under vacuum. The residue was purified by flash chromatography (3:1 petroleum ether/EtOAc) to provide **5b** as a yellow solid (0.54 g, 41%). Mp: 110-114 °C. IR:

3395 (br), 2932 (w), 1678 (m), 1503 (m), 1176 (s), 845 (m) cm^{-1}. ^1H NMR: δ 3.34 (2H, t, *J* = 5.5 Hz), 3.83 (3H, s), 3.87 (2H, q, *J* = 5.6 Hz), 6.84-6.92 (3H, m), 7.47 (2H, t, *J* = 7.5 Hz), 7.58 (1H, t, *J* = 7.5 Hz), 7.72 (2H, d, *J* = 8.8 Hz), 7.97 (2H, d, *J* = 8.4 Hz). ^{13}C {^1H} NMR: δ 35.1, 38.5, 55.6, 113.9 (2C), 126.9, 128.3 (2C), 128.9 (2C), 129.0 (2C), 133.7, 136.7, 162.3, 167.3, 199.9. HRMS (ESI-TOF) m/z: $[M+H]^+$ Cald for $C_{17}H_{18}NO_3$ 284.1281; Found 284.1280.

***N*-(3-(4-Chlorophenyl)-3-oxopropyl)benzamide, 5e.** Yield: 29% (0.21 g). White solid. Mp: 119-122 °C. IR: 3237 (br), 2924 (w), 1673 (m), 1297 (m), 1104 (s), 692 (s) cm^{-1}. ^1H NMR: δ 3.28 (2H, t, *J* = 5.5 Hz), 3.84 (2H, q, *J* = 5.7 Hz), 7.06 (1H, br), 7.33-7.48 (5H, m), 7.74 (2H, d, *J* = 7.7 Hz), 7.86 (2H, d, *J* = 7.7 Hz). ^{13}C {^1H} NMR: δ 35.2, 38.5, 127.2 (2C), 128.8 (2C), 129.3 (2C), 129.8 (2C), 131.2, 134.6, 135.0, 140.3, 167.8, 198.7. HRMS (ESI-TOF) m/z: $[M+H]^+$ Cald for $C_{16}H_{15}ClNO_2$ 288.0786; Found 288.1025.

***N*-(3-(Furan-2-yl)-3-oxopropyl)benzamide, 5j.** Yield: 54% (0.21 g). Light yellow solid. Mp: 92-96 °C. IR: 3362 (br), 2928 (w), 1627 (s), 1522 (s), 1281 (m), 688 (s) cm^{-1}. ^1H NMR: δ 3.19 (2H, t, *J* = 5.7 Hz), 3.85 (2H, q, *J* = 5.8 Hz), 6.53 (1H, dd, *J* = 3.7, 1.6 Hz), 6.97 (1H, br), 7.22 (1H, d, *J* = 3.7 Hz), 7.39 (2H, t, *J* = 7.6 Hz), 7.47 (1H, t, *J* = 7.6 Hz), 7.59 (1H, s), 7.74 (2H, d, *J* = 8.1 Hz). ^{13}C {^1H} NMR: δ 34.9, 38.2, 112.7, 118.2, 127.3 (2C), 128.9 (2C), 131.8, 134.7, 147.2, 152.6, 167.7, 188.9. HRMS (ESI-TOF) m/z: $[M+H]^+$ Cald for $C_{14}H_{14}NO_3$ 244.0929; Found 244.0969.

***N*-(3-Oxopentyl)benzamide, 5l.** Yield: 31% (0.17g). Yellow wax. IR: 3306 (br), 2935.8 (w), 1711 (s), 1633 (s), 1537 (s), 1115 (m) cm^{-1}. ^1H NMR: δ 1.03 (3H, t, *J* = 7.4 Hz), 2.42 (2H, q, *J* = 7.6 Hz), 2.74 (2H, t, *J* = 5.8 Hz), 3.66 (2H, q, *J* = 6.2 Hz), 6.97 (1H, br), 7.38 (2H, t, *J* = 7.2 Hz), 7.45 (1H, t, *J* = 7.2 Hz), 7.73 (2H, d, *J* = 7.6 Hz). ^{13}C {^1H} NMR: δ 7.98, 34.9, 36.5,

41.8, 127.2 (2C), 128.9 (2C), 131.8, 134.7, 167.6, 211.9. HRMS (ESI-TOF) m/z: $[M+H]^+$ Cald for $C_{12}H_{16}NO_2$ 206.1176; Found 206.1177.

***N*-(2-Methyl-3-oxobutyl)benzamide, 5m.** Yield: 50% (2.68 g). Yellow oil. IR: 3359 (br), 2362 (w), 1707 (s), 1645 (m), 1358 (s), 1220 (s) cm^{-1}. ^1H NMR: δ 1.17 (3H, d, *J* = 2.5 Hz), 2.15 (3H, s), 2.86-2.96 (1H, m), 3.42-5.51 (1H, m), 3.56-3.65 (1H, m), 6.89 (1H, br), 7.37 (2H, t, *J* = 7.3 Hz), 7.44 (1H, t, *J* = 7.3 Hz), 7.72 (2H, d, *J* = 7.7 Hz). ^{13}C {^1H} NMR: δ 14.4, 28.5, 41.6, 46.7, 127.0 (2C), 128.4 (2C), 131.4, 134.3, 167.7, 211.9. HRMS (ESI-TOF) m/z: $[M+H]^+$ Cald for $C_{12}H_{16}NO_2$ 206.1176; Found 206.1178.

4-Nitro-*N*-(3-oxo-3-phenylpropyl)benzamide, 5p. Yield: 73% (0.17 g). White solid. Mp: 135-138 °C. IR: 3364 (br), 2929 (w), 1673 (m), 1518 (s), 1213 (m), 781 (s) cm^{-1}. ^1H NMR: δ 3.37 (2H, t, *J* = 5.4 Hz), 3.92 (2H, q, *J* = 5.9 Hz), 7.10 (1H, br), 7.49 (2H, t, *J* = 7.7 Hz), 7.61 (1H, t, *J* = 7.4 Hz), 7.91 (2H, d, *J* = 8.7 Hz), 7.97 (2H, d, *J* = 8.5 Hz), 8.27 (2H, d, *J* = 8.5 Hz). ^{13}C {^1H} NMR: δ 35.5, 38.2, 124.1 (2C), 128.4 (2C), 128.5 (2C), 129.2 (2C), 134.2, 136.7, 140.3, 149.9, 165.7, 200.2. HRMS (ESI-TOF) m/z: $[M+H]^+$ Cald for $C_{16}H_{15}N_2O_4$ 299.1026; Found 299.1025.

Representative procedure for 2-iodoanisole-catalyzed cyclization of *N*-propargylamide 4 or β-amidoketone 5: Propargylamide **4** (1 equiv) or β-amidoketone **5** (1 equiv) was dissolved in acetonitrile (0.14 M) and 2-iodoanisole (0.2 equiv) was added, followed by *m*-CBPA (3 equiv) and *p*-TsOH·H_2O (3 equiv). The mixture was stirred overnight at room temperature, then saturated aqueous $Na_2S_2O_3$ solution and saturated aqueous $NaHCO_3$ solution were added and the mixture extracted with CH_2Cl_2. The organic layers were combined and dried with $MgSO_4$, filtered and concentrated under vacuum. The product was purified by flash chromatography (9:1 petroleum ether/EtOAc) to provide oxazoline **6**.

Phenyl(2-phenyl-4,5-dihydrooxazol-5-yl)methanone, 6a. Yield: 77% (0.076 g). Yellow solid. Mp: 104-108 °C. IR: 2922 (br), 2355 (w), 1702 (m), 1650 (s), 1596 (w), 685 (s) cm^{-1}. ^{1}H NMR: δ 4.28 (1H, dd, *J* = 15, 7.6 Hz), 4.46 (1H, dd, *J* = 15, 11 Hz), 5.86 (1H, dd, *J* = 11, 7.7 Hz), 7.42 (2H, t, *J* = 7.5 Hz), 7.45-7.56 (3H, m), 7.63 (1H, t, *J* = 7.3 Hz) 7.99 (4H, d, *J* = 7.7 Hz). ^{13}C {^{1}H} NMR: δ 58.8, 79.9, 127.3, 128.8 (3C), 129.1 (2C), 129.3 (2C), 132.0 (2C), 134.3, 134.4, 164.5, 195.2. HRMS (ESI-TOF) m/z: $[M+H]^+$ Cald for $C_{16}H_{14}NO_2$ 252.0980; Found 252.1026.

(2-(4-Methoxyphenyl)-4,5-dihydrooxazol-5-yl)(phenyl)methanone, 6b. Yield: 77% (0.073 g). Yellow solid. Mp: 134-137 °C. IR: 2924 (br), 2849 (w), 1694 (m), 1604 (m), 1378 (m), 1301 (s), 688 (s) cm^{-1}. ^{1}H NMR: δ 3.85 (3H, s), 4.24 (1H, dd, *J* = 15, 7.7 Hz,), 4.44 (1H, dd, *J* = 15, 11 Hz), 5.82 (1H, dd, *J* = 11, 7.7 Hz), 6.92 (2H, d, *J* = 8.7 Hz), 7.52 (2H, t, *J* = 7.7 Hz), 7.64 (1H, t, *J* = 7.6 Hz), 7.93 (2H, d, *J* = 8.8 Hz), 7.99 (2H, d, *J* = 7.7 Hz). ^{13}C {^{1}H} NMR: δ 55.7, 58.9, 79.9, 114.1 (2C), 119.9, 129.1 (2C), 129.3 (2C), 130.5 (2C), 134.3, 134.5, 162.7, 164.3, 195.5. HRMS (ESI-TOF) m/z: $[M+H]^+$ Cald for $C_{17}H_{16}NO_3$ 282.1085; Found 282.1123.

(2-(4-Chlorophenyl)-4,5-dihydrooxazol-5-yl)(phenyl)methanone, 6c. Yield: 75% (0.079 g). White solid. Mp: 103-107 °C. IR: 2927 (br), 1702 (m), 1649 (m), 1488 (m), 1089 (s), 850 (s) cm^{-1}. ^{1}H NMR: δ 4.26 (1H, dd, *J* = 15, 7.5 Hz), 4.46 (1H, dd, *J* = 15, 11 Hz,), 5.87 (1H, dd, *J* = 11, 7.5 Hz), 7.39 (2H, t, *J* = 8.4 Hz), 7.52 (2H, d, *J* = 7.7 Hz), 7.64 (1H, t, *J* = 7.3 Hz), 7.92 (2H, d, *J* = 8.3 Hz), 7.98 (2H, d, *J* = 8.3 Hz). ^{13}C {^{1}H} NMR: δ 58.9, 80.0, 125.8, 129.0 (3C), 129.1 (2C), 129.3 (2C), 130.1, 134.3, 134.4, 138.3, 163.7, 195.0. HRMS (ESI-TOF) m/z: $[M+H]^+$ Cald for $C_{16}H_{13}ClNO_2$ 286.0629; Found 286.6020.

(2-(Furan-2-yl)-4,5-dihydrooxazol-5-yl)(phenyl)methanone, 6d. Yield: 66% (0.071 g). Yellow solid. Mp: 76-79 °C. IR: 3111 (br), 2924 (s), 1700 (s), 1672 (s), 1479 (m), 1097 (s)

cm^{-1}. ^{1}H NMR: δ 4.24 (1H, dd, *J* = 15, 7.7 Hz), 4.47 (1H, dd, *J* = 15, 11 Hz), 5.84 (1H, dd, *J* = 11, 7.7 Hz), 6.51 (1H, s), 7.05 (1H, d, *J* = 3.7 Hz), 7.52 (2H, t, *J* = 7.7 Hz), 7.57 (1H, s), 7.64 (1H, t, *J* = 7.7 Hz), 7.97 (2H, d, *J* = 7.9 Hz). ^{13}C {^{1}H} NMR: δ 58.9, 79.9, 111.9, 115.5, 129.1 (2C), 129.3 (2C), 134.3, 134.4, 142.6, 145.9, 156.7, 194.8. HRMS (ESI-TOF) m/z: $[M+H]^+$ Cald for $C_{14}H_{12}NO_3$ 242.0812; Found 242.0818.

(4-Chlorophenyl)(2-phenyl-4,5-dihydrooxazol-5-yl)methanone, 6e. Yield: 75% (0.079 g). Yellow solid. Mp: 106-108 °C. IR: 3063 (br), 2848 (w), 1693 (m), 1586 (m), 1362 (m), 1058 (m), 710 (s) cm^{-1}. ^{1}H NMR: δ 4.31 (1H, dd, *J* = 15, 7.5 Hz), 4.44 (1H, dd, *J* = 15, 11 Hz), 5.78 (1H, dd, *J* = 11, 7.5 Hz), 7.42 (2H, t, *J* = 7.6 Hz), 7.47-7.53 (3H, m), 7.93-7.99 (4H, m). ^{13}C {^{1}H} NMR: δ 58.6, 79.9, 127.3, 128.7 (2C), 128.8 (2C), 129.7 (2C), 130.6 (2C), 132.1, 132.9, 140.9, 164.2, 194.4. HRMS (ESI-TOF) m/z: $[M+H]^+$ Cald for $C_{16}H_{13}ClNO_2$ 286.0629; Found 286.6043.

[1,1'-Biphenyl]-2-yl(2-phenyl-4,5-dihydrooxazol-5-yl)methanone, 6f. Yield: 75% (0.078 g). Yellow wax. IR: 3055 (br), 2926 (w), 2871 (w), 1698 (m), 1646 (m), 1252 (m) cm^{-1}. ^{1}H NMR: δ 3.90 (1H, dd, *J* = 15, 11 Hz), 3.99 (1H, dd, *J* = 15, 7.4 Hz), 4.81 (1H, dd, 11, 7.4 Hz), 7.33-7.41 (4H, m), 7.42-7.50 (6H, m), 7.52-7.61 (2H, m), 7.76 (2H, d, *J* = 8.1 Hz). ^{13}C {^{1}H} NMR: δ 59.2, 81.4, 127.3, 128.0, 128.6 (5C), 129.0, 129.3 (2C), 129.4 (2C), 130.5, 131.8, 131.9, 137.8, 140.4, 141.2, 164.3, 205.9. HRMS (ESI-TOF) m/z: $[M+H]^+$ Cald for $C_{22}H_{18}NO_2$ 328.1332; Found 328.1329.

(2-Phenyl-4,5-dihydrooxazol-5-yl)(*p*-tolyl)methanone, 6g. Yield: 35% (0.037 g). Yellow solid. Mp: 119-122 °C. IR: 2924 (br), 1722 (w), 1689 (w), 1577 (w), 1247 (s), 709 (s) cm^{-1}. ^{1}H NMR: δ 2.44 (3H, m), 4.26 (1H, dd, *J* = 15, 7.7 Hz), 4.45 (1H, dd, *J* = 15, 11 Hz), 5.84 (1H, dd, *J* = 11, 7.7 Hz), 7.32 (2H, d, *J* = 8.1 Hz), 7.42 (2H, t, *J* = 7.7 Hz), 7.49 (1H, t, *J* = 7.4 Hz), 7.89 (2H, d, *J* = 8.1 Hz), 7.99 (2H, d, *J* = 8.1 Hz). ^{13}C {^{1}H} NMR: δ 22.2, 58.9, 79.9,

127.4, 128.7 (4C), 128.8 (2C), 129.2 (2C), 130.0, 132.0, 145.4, 164.5, 194.9. HRMS (ESI-TOF) m/z: $[M+H]^+$ Cald for $C_{17}H_{16}NO_2$ 266.1176; Found 266.1176.

Naphthalen-2-yl(2-phenyl-4,5-dihydrooxazol-5-yl)methanone, 6h. Yield: 82% (0.072 g). Light yellow oil. IR: 3339 (br), 2968 (m), 1694 (w), 1650 (m), 1508 (s), 950 (s) cm^{-1}. ^1H NMR: δ 4.30 (1H, dd, *J* = 15, 7.4 Hz), 4.43 (1H, dd, *J* = 15, 11 Hz), 5.93 (1H, dd, *J* = 11, 7.4 Hz), 7.41 (2H, t, *J* = 7.5 Hz), 7.49 (1H, t, *J* = 7.5 Hz), 7.52-7.65 (3H, m), 7.91 (2H, t, *J* = 8.7 Hz), 7.97 (2H, d, *J* = 7.7 Hz), 8.06 (1H, d, *J* = 8.1 Hz), 8.64 (1H, d, *J* = 8.5 Hz). $^{13}C\ \{^1H\}$ NMR: δ 59.3, 81.1, 124.6, 125.9, 127.2, 127.4, 128.7 (2C), 128.7 (2C), 128.8, 128.8, 128.9, 131.1, 132.0, 132.6, 134.1, 134.3, 164.6, 199.4. HRMS (ESI-TOF) m/z: $[M+H]^+$ Cald for $C_{20}H_{16}NO_2$ 302.1176; Found 302.1193.

(4-Methoxyphenyl)(2-phenyl-4,5-dihydrooxazol-5-yl)methanone, 6i. Yield: 52% (0.051 g). Yellowish wax. IR: 3324 (br), 2924 (w), 2836 (w), 1740 (m), 1646 (m), 1598 (s), 1169 (m), 695 (s) cm^{-1}. ^1H NMR: δ 3.89 (3H, s), 4.29 (1H, dd, *J* = 15, 7.7 Hz,), 4.45 (1H, dd, *J* = 15, 11 Hz), 5.82 (1H, dd, *J* = 11, 7.7 Hz), 6.99 (2H, d, *J* = 8.9 Hz), 7.42 (2H, t, *J* = 7.7 Hz), 7.49 (1H, t, *J* = 7.4 Hz), 7.99 (2 x 2H, d, *J* = 7.8 Hz). $^{13}C\ \{^1H\}$ NMR: δ 55.9, 58.8, 79.9, 114.5 (2C), 127.4, 127.5, 128.8 (5C), 131.5 (2C), 132.0, 164.5, 193.7. HRMS (ESI-TOF) m/z: $[M+H]^+$ Cald for $C_{17}H_{16}NO_3$ 282.1125; Found 282.1134.

Furan-2-yl(2-phenyl-4,5-dihydrooxazol-5-yl)methanone, 6j. Yield: 95% (0.095 g). Brown solid. Mp: 75-77 °C. IR: 3335 (br), 2969 (s), 1466 (w), 1378 (m), 1127 (m), 950 (s) cm^{-1}. ^1H NMR: δ 4.25 (1H, dd, *J* = 15, 7.8 Hz), 4.47 (1H, dd, *J* = 15, 11 Hz), 5.61 (1H, dd, *J* = 11, 7.8 Hz), 6.58 (1H, dd, *J* = 3.8, 1.8 Hz), 7.41 (1H, t, *J* = 3.9 Hz), 7.45 (2H, d, *J* = 7.9 Hz), 7.51 (1H, t, *J* = 7.3 Hz), 7.71 (1H, s), 8.00 (2H, d, *J* = 7.7 Hz). $^{13}C\ \{^1H\}$ NMR: δ 59.2, 80.1, 113.0, 120.1, 127.4, 128.8 (2C), 128.8 (2C), 132.1, 147.9, 150.7, 164.3, 185.0. HRMS (ESI-TOF) m/z: $[M+H]^+$ Cald for $C_{14}H_{12}NO_3$ 242.0812; Found 242.0804.

1-(2-Phenyl-4,5-dihydrooxazol-5-yl)ethan-1-one, 6k. Yield: 63% **(**0.06 g). Yellow oil. IR: 2937 (br), 1717 (s), 1651 (s), 1254 (m), 1058 (s), 778 (m) cm^{-1}. ^{1}H NMR: δ 2.28 (3H, s), 4.09 (1H, dd, *J* = 15, 7.3 Hz), 4.33 (1H, dd, *J* = 15, 11 Hz), 4.96 (1H, dd, *J* = 11, 7.3 Hz), 7.45 (2H, t, *J* = 7.6 Hz), 7.53 (1H, t, *J* = 7.6 Hz), 7.99 (2H, d, *J* = 7.3 Hz). ^{13}C {^{1}H} NMR: δ 26.3, 59.0, 82.8, 127.3, 128.6 (2C), 128.9 (2C), 132.2, 164.2, 208.1. HRMS (ESI-TOF) m/z: $[M+H]^+$ Cald for $C_{11}H_{12}NO_2$ 190.0863; Found 190.0866.

1-(2-Phenyl-4,5-dihydrooxazol-5-yl)propan-1-one, 6l. Yield: 56% (0.055 g). Colorless oil. IR: 2969 (br), 1717 (m), 1643 (w), 1451 (w), 1264 (m), 711 (s) cm^{-1}. ^{1}H NMR: δ 1.09 (3H, t, *J* = 7.3 Hz), 2.52-2.64 (1H, m), 2.67-2.79 (1H, m), 4.08 (1H, dd, *J* = 15, 7.2 Hz), 4.33 (1H, dd, *J* = 15, 11 Hz), 4.99 (1H, dd, *J* = 11, 7.2 Hz), 7.45 (2H, t, *J* = 7.4 Hz), 7.53 (1H, t, *J* = 7.4 Hz), 7.99 (2H, d, *J* = 7.8 Hz). ^{13}C {^{1}H} NMR: δ 7.2, 32.2, 59.3, 82.6, 127.4, 128.6 (2C), 128.9 (2C), 132.2, 164.3, 210.8. HRMS (ESI-TOF) m/z: $[M+NH_4]^+$ Cald for $C_{12}H_{17}N_2O_2$ 221.1285; Found 221.1284.

1-(5-Methyl-2-phenyl-4,5-dihydrooxazol-5-yl)ethanone, 6m. Yield: 75% (0.075 g). Colorless oil. IR: 3335 (br), 2969 (m), 2928 (w), 2359 (m), 1713 (m), 1646 (m), 950 (s) cm^{-1}. ^{1}H NMR: δ 1.58 (3H, s), 2.29 (3H, s), 3.85 (1H, d, *J* = 15 Hz), 4.21 (1H, d, *J* = 15 Hz), 7.44 (2H, t, *J* = 7.5 Hz), 7.52 (1H, t, *J* = 7.5 Hz), 7.99 (2H, d, *J* = 7.3 Hz). ^{13}C {^{1}H} NMR: δ 23.1, 25.5, 65.1, 89.8, 127.7, 128.5 (2C), 128.8 (2C), 132.0, 163.5, 209.9. HRMS (ESI-TOF) m/z: $[M+H]^+$ Cald for $C_{12}H_{14}NO_2$ 204.1019; Found 204.1012.

((*syn*)-4-Methyl-2-phenyl-4,5-dihydrooxazol-5-yl)(phenyl)methanone, *syn*-6n. Yield: 54% (0.053 g). Yellow wax. IR: 2925 (br), 1698 (m), 1647 (m), 1448 (m), 1218 (s), 687 (s) cm^{-1}. ^{1}H NMR: δ 1.09 (3H, d, *J* = 7.0 Hz), 4.89 (1H, dq, *J* = 10, 6.9 Hz), 6.00 (1H, d, *J* = 10 Hz), 7.44 (2H, t, *J* = 7.6 Hz), 7.49-7.56 (3H, m), 7.64 (1H, t, *J* = 7.3 Hz), 7.95 (2H, d, *J* = 8.2 Hz), 8.03 (2H, d, *J* = 8.2 Hz). ^{13}C {^{1}H} NMR: δ 17.8, 65.5, 83.6, 127.4, 128.4 (2C), 128.8

(2C), 128.9 (2C), 129.4 (2C), 132.1, 134.3, 135.8, 163.7, 194.7. HRMS (ESI-TOF) m/z: $[M+H]^+$ Cald for $C_{17}H_{16}NO_2$ 266.1176; Found 266.1167.

((*anti*)-4-Methyl-2-phenyl-4,5-dihydrooxazol-5-yl)(phenyl)methanone, *anti*-6n. Yield: 34% **(**0.034 g). Yellow solid. Mp: 71-73 °C. IR: 2925 (br), 1698 (m), 1647 (m), 1448 (m), 1218 (s), 687 (s) cm^{-1}. ^{1}H NMR: δ 1.54 (3H, d, *J* = 6.7 Hz), 4.52 (1H, pent, *J* = 6.6 Hz), 5.39 (1H, d, *J* = 6.7 Hz), 7.42 (2H, t, *J* = 7.6 Hz), 7.50 (1H, t, *J* = 7.8 Hz), 7.52 (2H, t, *J* = 7.8 Hz), 7.63 (1H, t, *J* = 7.5 Hz), 7.99 (2 x 2H, d, *J* = 7.8 Hz). ^{13}C {^{1}H} NMR: δ 22.0, 66.5, 86.4, 127.5, 128.7 (2C), 128.8 (2C), 129.1 (2C), 129.2 (2C), 132.0, 134.3, 134.8, 163.0, 195.7. HRMS (ESI-TOF) m/z: $[M+H]^+$ Cald for $C_{17}H_{16}NO_2$ 266.1176; Found 266.1167.

((*syn*)-2,4-Diphenyl-4,5-dihydrooxazol-5-yl)(phenyl)methanone, *syn*-6o. Yield: 18% (0.025 g). Yellow oil. IR: 2918 (br), 1700 (m), 1644 (s), 1447 (m), 1238 (m), 1068 (m) cm^{-1}. ^{1}H NMR: δ 5.83 (1H, d, *J* = 11 Hz), 6.29 (1H, d, *J* = 11 Hz), 6.93 (2H, dd, *J* = 7.5, 1.8 Hz), 6.98-7.05 (3H, m), 7.31 (2H, t, *J* = 7.7 Hz), 7.43-7.60 (6H, m), 8.06 (2H, d, *J* = 8.1 Hz). ^{13}C {^{1}H} NMR: δ 74.5, 84.8, 127.3, 128.1 (2C), 128.3 (3C), 128.6 (2C), 128.8 (2C), 128.9 (2C), 129.2 (2C), 132.4, 133.6, 136.2, 136.7, 165.5, 194.7. HRMS (ESI-TOF) m/z: $[M+H]^+$ Cald for $C_{22}H_{18}NO_2$ 328.1331; Found 328.1330.

((*anti*)-2,4-Diphenyl-4,5-dihydrooxazol-5-yl)(phenyl)methanone, *anti*-6o.[8] Yield: 30% (0.041 g). Yellow oil. IR: 2918 (br), 1700 (m), 1644 (s), 1447 (m), 1238 (m), 1068 (m) cm^{-1}. ^{1}H NMR: δ 5.53 (1H, d, *J* = 6.6 Hz), 5.71 (1H, d, *J* = 6.6 Hz), 7.29-7.40 (5H, m), 7.45-7.57 (5H, m), 7.63 (1H, t, *J* = 7.5 Hz), 7.94 (2H, t, *J* = 7.2 Hz), 8.09 (2H, d, *J* = 7.3 Hz). ^{13}C {^{1}H} NMR: δ 74.0, 87.1, 127.3, 127.4 (2C), 128.6, 128.8 (2C), 129.1 (3C), 129.2 (2C), 129.4 (2C),

[8] Hajra, S.; Bar, S.; Sinha, D.; Maji, B. *J. Org. Chem.* **2008**, *73*, 4320.

129.5 (2C), 132.3, 134.4, 141.4, 164.2, 194.8. HRMS (ESI-TOF) m/z: $[M+H]^+$ Cald for $C_{22}H_{18}NO_2$ 328.1331; Found 328.1330.

***N*-(2-Hydroxy-3-oxo-3-phenylpropyl)-4-nitrobenzamide, 8.** Yield: 66% (0.069 g). Light yellow solid. Mp: 75-77 °C. IR: 3405 (br), 2969 (m), 1667 (m), 1535 (m), 1368 (w), 1124 (m), 950 (s) cm^{-1}. ^1H NMR: δ 3.33 (1H, ddd, *J* = 13, 8.3, 5.5 Hz), 4.09 (1H, br), 4.24 (1H, ddd, *J* = 10, 6.8, 3.2 Hz), 5.34 (1H, dd, *J* = 7.7, 2.8 Hz), 6.87 (1H, br), 7.57 (2H, t, *J* = 7.7 Hz), 7.68 (1H, t, *J* = 7.7, Hz), 7.95 (2H, d, *J* = 8.7 Hz), 8.12 (2H, d, *J* = 8.2 Hz), 8.29 (2H, d, J = 8.7 Hz). ^{13}C {^1H} NMR: δ 45.7, 72.8, 124.2 (2C), 128.6 (2C), 129.2 (2C), 129.6 (2C), 133.3, 135.2, 139.8, 150.1, 166.3, 199.4. HRMS (ESI-TOF) m/z: $[M+H]^+$ Cald for $C_{16}H_{15}N_2O_5$ 315.0936; Found 315.0972.

^{1}H NMR and ^{13}C NMR spectra.

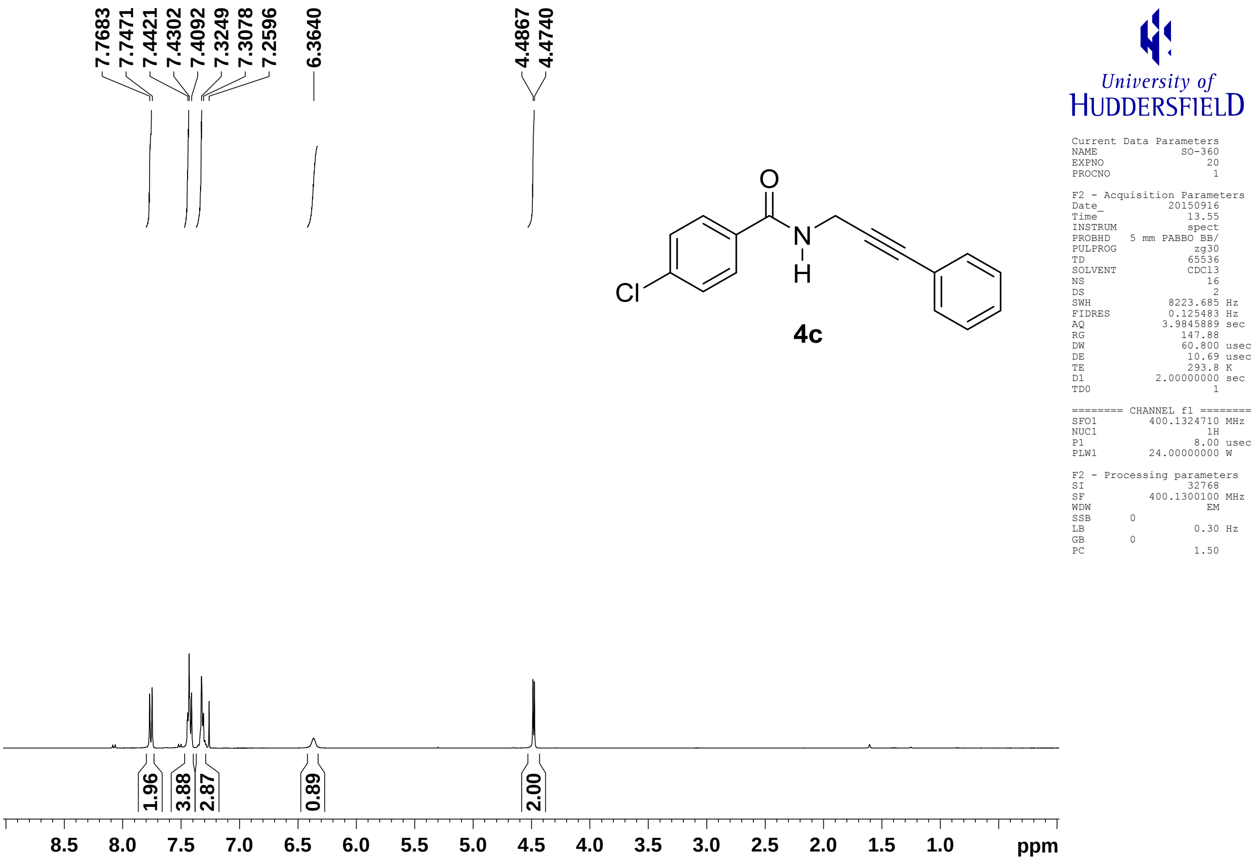

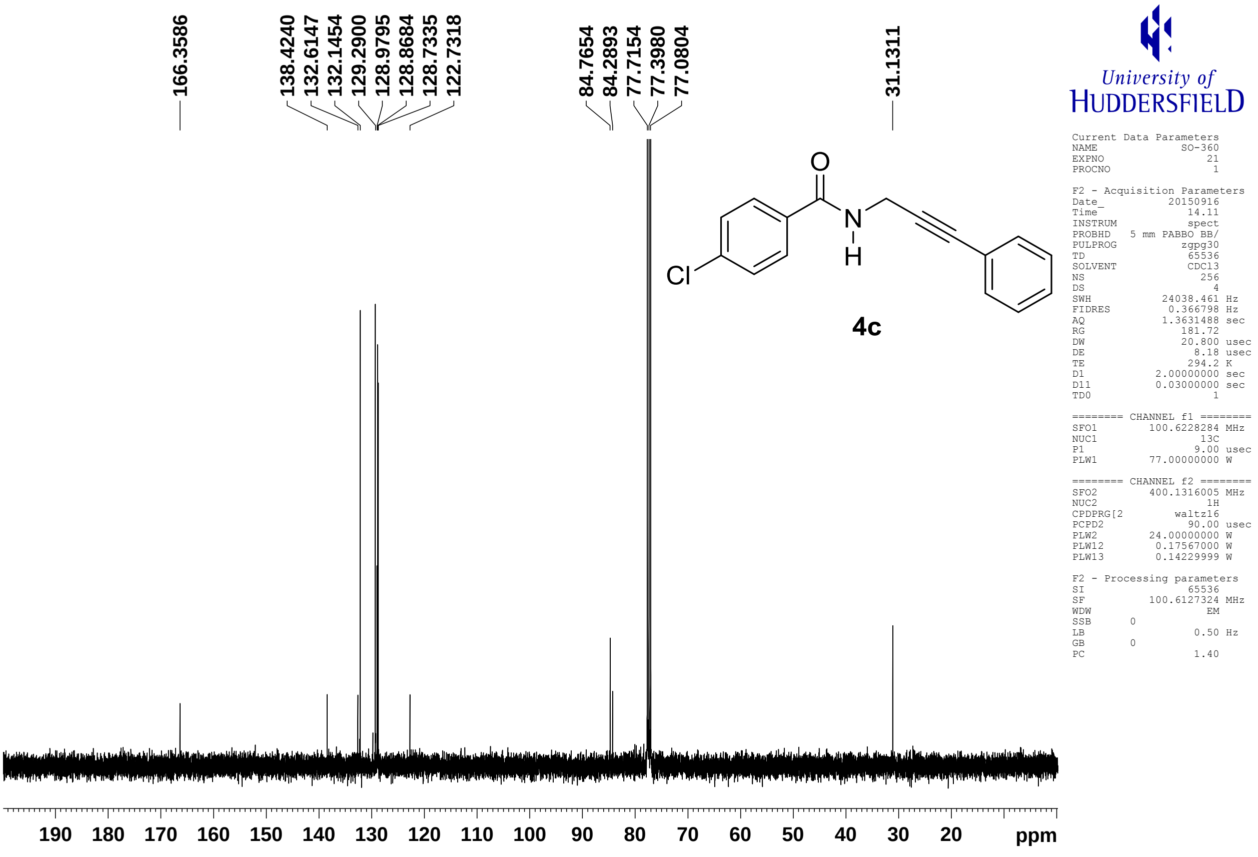

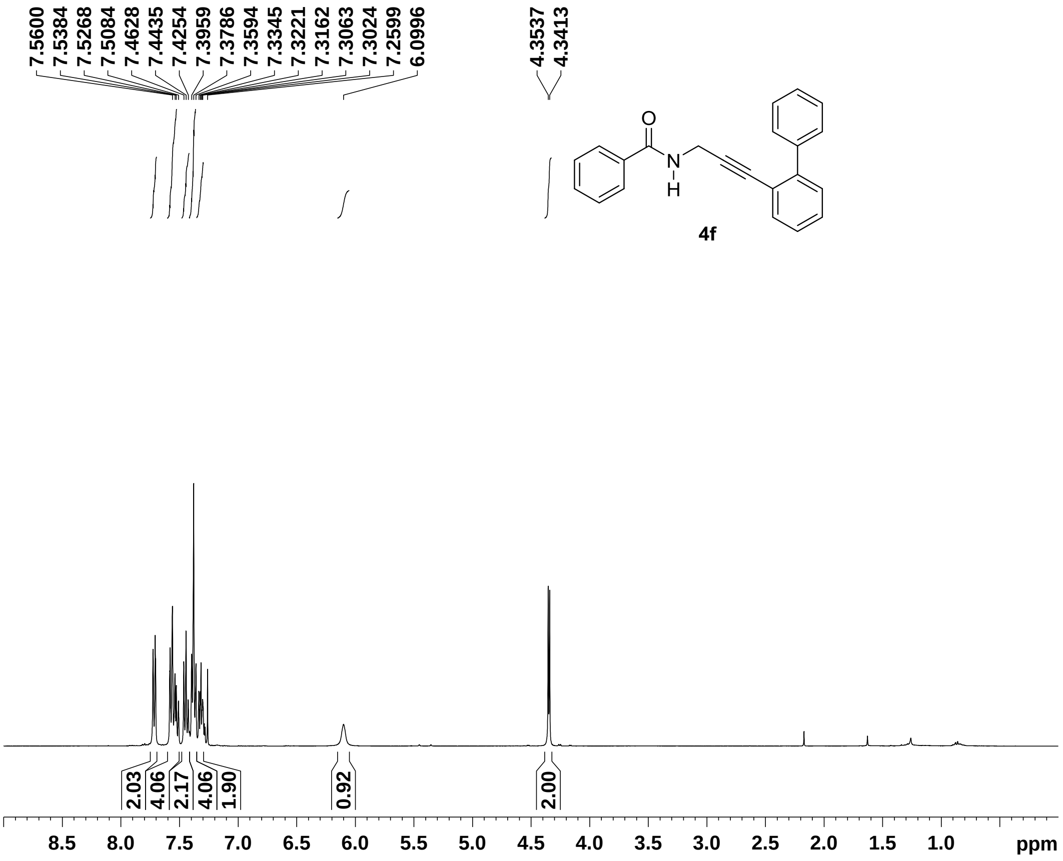

University of HUDDERSFIELD

```
Current Data Parameters
NAME                SO-395
EXPNO                   60
PROCNO                   1

F2 - Acquisition Parameters
Date_             20151020
Time                 16.14
INSTRUM              spect
PROBHD   5 mm PABBO BB/
PULPROG               zg30
TD                   65536
SOLVENT              CDCl3
NS                      16
DS                       2
SWH               8223.685 Hz
FIDRES            0.125483 Hz
AQ               3.9845889 sec
RG                  104.33
DW                  60.800 usec
DE                   10.69 usec
TE                   293.8 K
D1              2.00000000 sec
TD0                      1

======== CHANNEL f1 ========
SFO1           400.1324710 MHz
NUC1                    1H
P1                    8.00 usec
PLW1           24.00000000 W

F2 - Processing parameters
SI                   32768
SF             400.1300097 MHz
WDW                     EM
SSB      0
LB                    0.30 Hz
GB       0
PC                    1.50
```

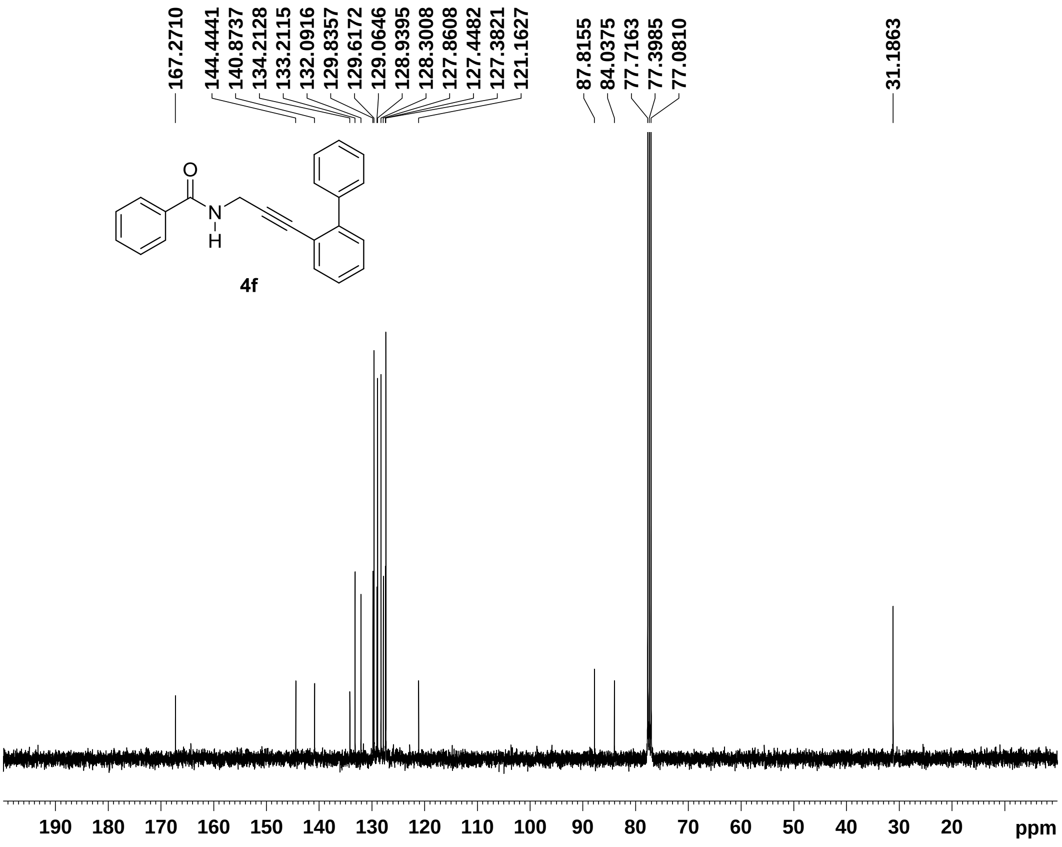

University of HUDDERSFIELD

```
Current Data Parameters
NAME                SO-395
EXPNO                   61
PROCNO                   1

F2 - Acquisition Parameters
Date_             20151020
Time                 16.30
INSTRUM              spect
PROBHD   5 mm PABBO BB/
PULPROG             zgpg30
TD                   65536
SOLVENT              CDCl3
NS                     256
DS                       4
SWH              24038.461 Hz
FIDRES            0.366798 Hz
AQ               1.3631488 sec
RG                  181.72
DW                  20.800 usec
DE                    8.18 usec
TE                   294.3 K
D1              2.00000000 sec
D11             0.03000000 sec
TD0                      1

======== CHANNEL f1 ========
SFO1           100.6228303 MHz
NUC1                   13C
P1                    9.00 usec
PLW1           77.00000000 W

======== CHANNEL f2 ========
SFO2           400.1316005 MHz
NUC2                    1H
CPDPRG[2           waltz16
PCPD2                90.00 usec
PLW2           24.00000000 W
PLW12           0.17567000 W
PLW13           0.14229999 W

F2 - Processing parameters
SI                   65536
SF             100.6127337 MHz
WDW                     EM
SSB      0
LB                    1.00 Hz
GB       0
PC                    1.40
```

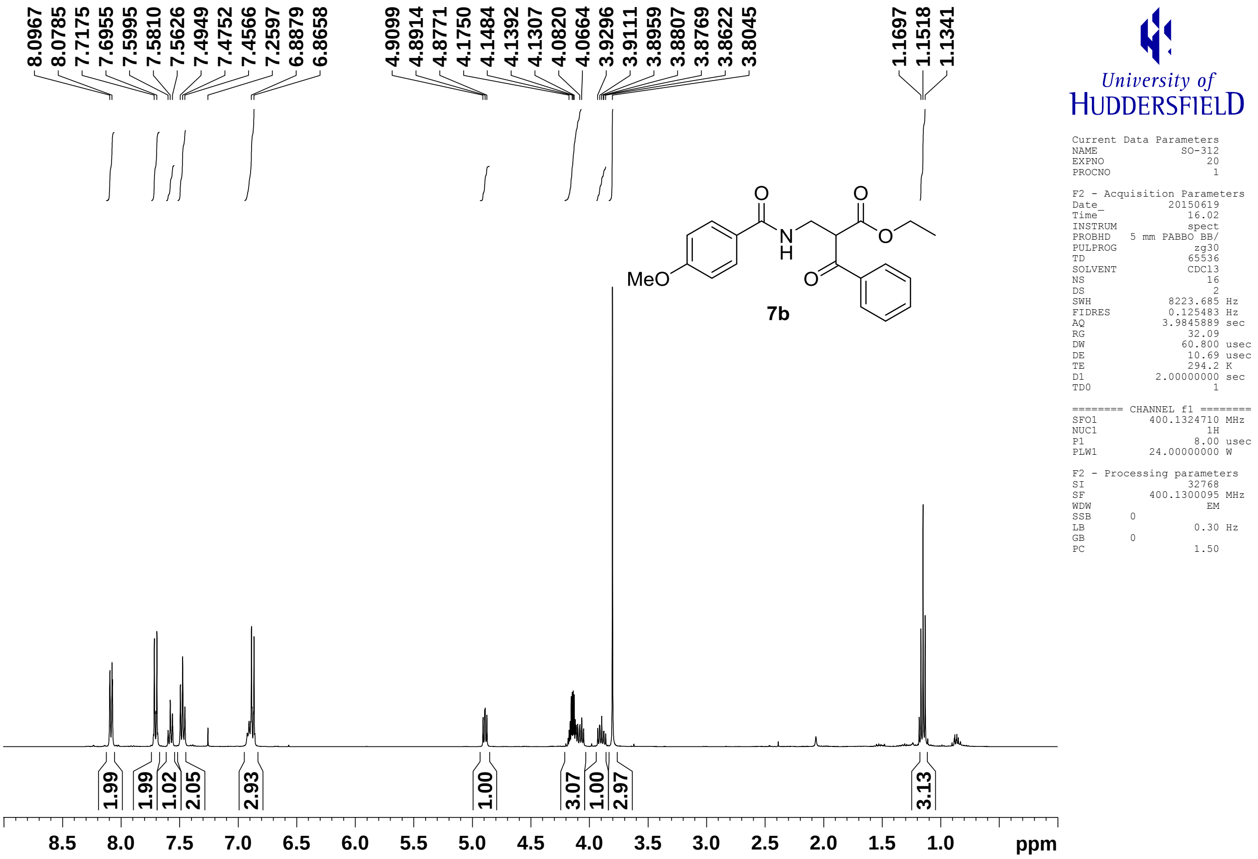
8.0967 8.0785 7.7175 7.6955 7.5995 7.5810 7.5626 7.4949 7.4752 7.4566 7.2597 6.8879 6.8658
4.9099 4.8914 4.8771 4.1750 4.1484 4.1392 4.1307 4.0820 4.0664 3.9296 3.9111 3.8959 3.8807 3.8769 3.8622 3.8045
1.1697 1.1518 1.1341
MeO
O
N
H
7b
1.99 1.99 1.02 2.05 2.93 1.00 3.07 1.00 2.97 3.13
8.5 8.0 7.5 7.0 6.5 6.0 5.5 5.0 4.5 4.0 3.5 3.0 2.5 2.0 1.5 1.0 ppm
University of HUDDERSFIELD
Current Data Parameters
NAME SO-312
EXPNO 20
PROCNO 1
F2 - Acquisition Parameters
Date_ 20150619
Time 16.02
INSTRUM spect
PROBHD 5 mm PABBO BB/
PULPROG zg30
TD 65536
SOLVENT CDCl3
NS 16
DS 2
SWH 8223.685 Hz
FIDRES 0.125483 Hz
AQ 3.9845889 sec
RG 32.09
DW 60.800 usec
DE 10.69 usec
TE 294.2 K
D1 2.00000000 sec
TD0 1
======== CHANNEL f1 ========
SFO1 400.1324710 MHz
NUC1 1H
P1 8.00 usec
PLW1 24.00000000 W
F2 - Processing parameters
SI 32768
SF 400.1300095 MHz
WDW EM
SSB 0
LB 0.30 Hz
GB 0
PC 1.50

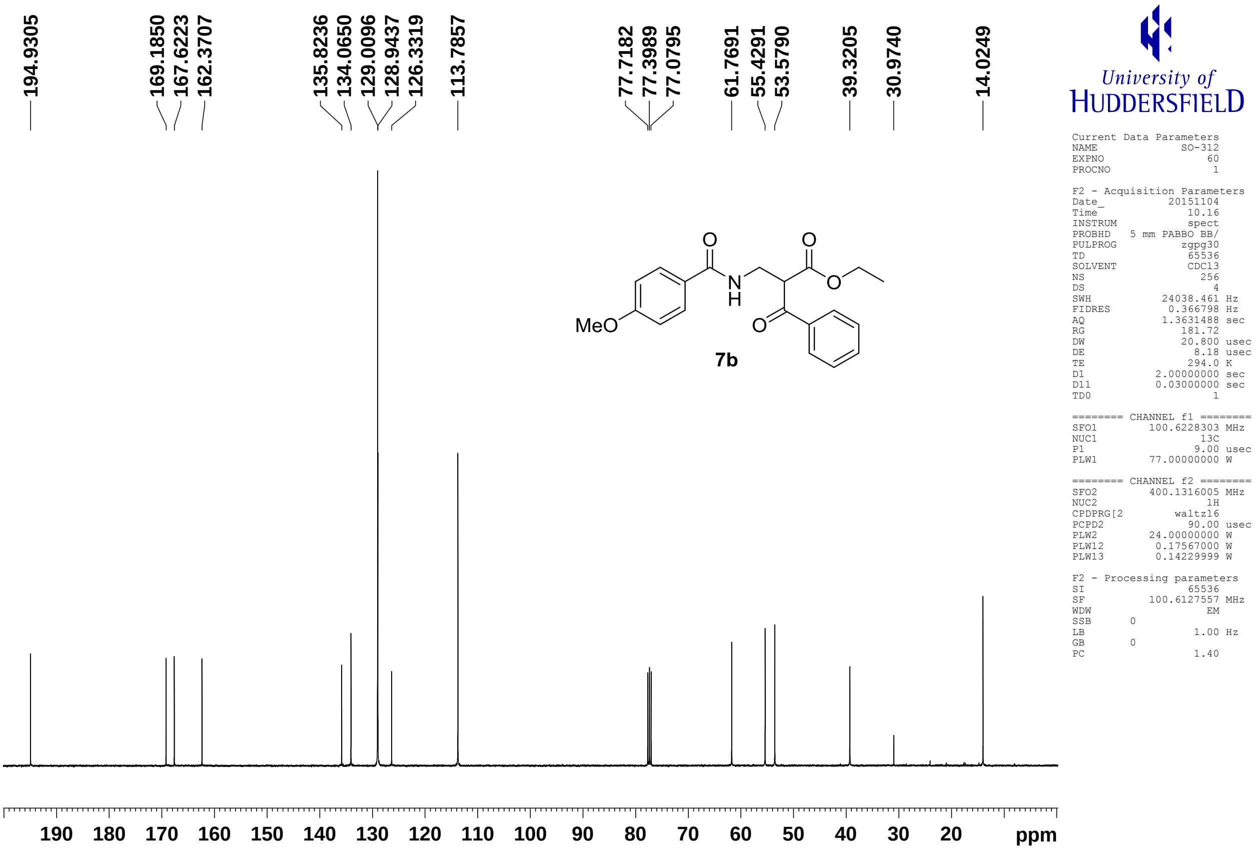
194.9305
169.1850 167.6223 162.3707
135.8236 134.0650 129.0096 128.9437 126.3319 113.7857
77.7182 77.3989 77.0795
61.7691 55.4291 53.5790
39.3205 30.9740
14.0249
MeO
O
N
H
7b
190 180 170 160 150 140 130 120 110 100 90 80 70 60 50 40 30 20 ppm
University of HUDDERSFIELD
Current Data Parameters
NAME SO-312
EXPNO 60
PROCNO 1
F2 - Acquisition Parameters
Date_ 20151104
Time 10.16
INSTRUM spect
PROBHD 5 mm PABBO BB/
PULPROG zgpg30
TD 65536
SOLVENT CDCl3
NS 256
DS 4
SWH 24038.461 Hz
FIDRES 0.366798 Hz
AQ 1.3631488 sec
RG 181.72
DW 20.800 usec
DE 8.18 usec
TE 294.0 K
D1 2.00000000 sec
D11 0.03000000 sec
TD0 1
======== CHANNEL f1 ========
SFO1 100.6228303 MHz
NUC1 13C
P1 9.00 usec
PLW1 77.00000000 W
======== CHANNEL f2 ========
SFO2 400.1316005 MHz
NUC2 1H
CPDPRG[2 waltz16
PCPD2 90.00 usec
PLW2 24.00000000 W
PLW12 0.17567000 W
PLW13 0.14229999 W
F2 - Processing parameters
SI 65536
SF 100.6127557 MHz
WDW EM
SSB 0
LB 1.00 Hz
GB 0
PC 1.40

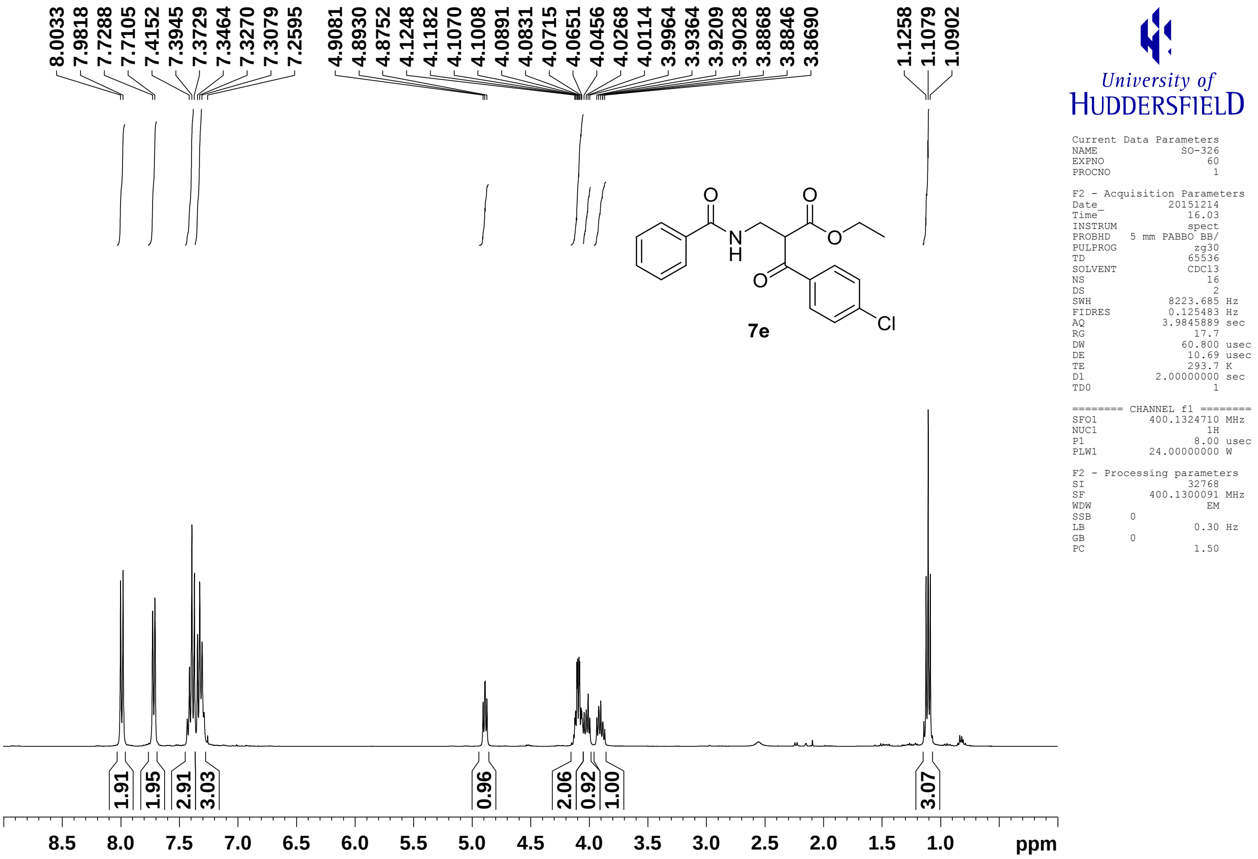
8.0033
7.9818
7.7288
7.7105
7.4152
7.3945
7.3729
7.3464
7.3270
7.3079
7.2595
4.9081
4.8930
4.8752
4.1248
4.1182
4.1070
4.1008
4.0891
4.0831
4.0715
4.0651
4.0456
4.0268
4.0114
3.9964
3.9364
3.9209
3.9028
3.8868
3.8846
3.8690
1.1258
1.1079
1.0902
7e
Cl
1.91
1.95
2.91
3.03
0.96
2.06
0.92
1.00
3.07
8.5 8.0 7.5 7.0 6.5 6.0 5.5 5.0 4.5 4.0 3.5 3.0 2.5 2.0 1.5 1.0 ppm
University of HUDDERSFIELD
Current Data Parameters
NAME SO-326
EXPNO 60
PROCNO 1
F2 - Acquisition Parameters
Date_ 20151214
Time 16.03
INSTRUM spect
PROBHD 5 mm PABBO BB/
PULPROG zg30
TD 65536
SOLVENT CDCl3
NS 16
DS 2
SWH 8223.685 Hz
FIDRES 0.125483 Hz
AQ 3.9845889 sec
RG 17.7
DW 60.800 usec
DE 10.69 usec
TE 293.7 K
D1 2.00000000 sec
TD0 1
======== CHANNEL f1 ========
SFO1 400.1324710 MHz
NUC1 1H
P1 8.00 usec
PLW1 24.00000000 W
F2 - Processing parameters
SI 32768
SF 400.1300091 MHz
WDW EM
SSB 0
LB 0.30 Hz
GB 0
PC 1.50

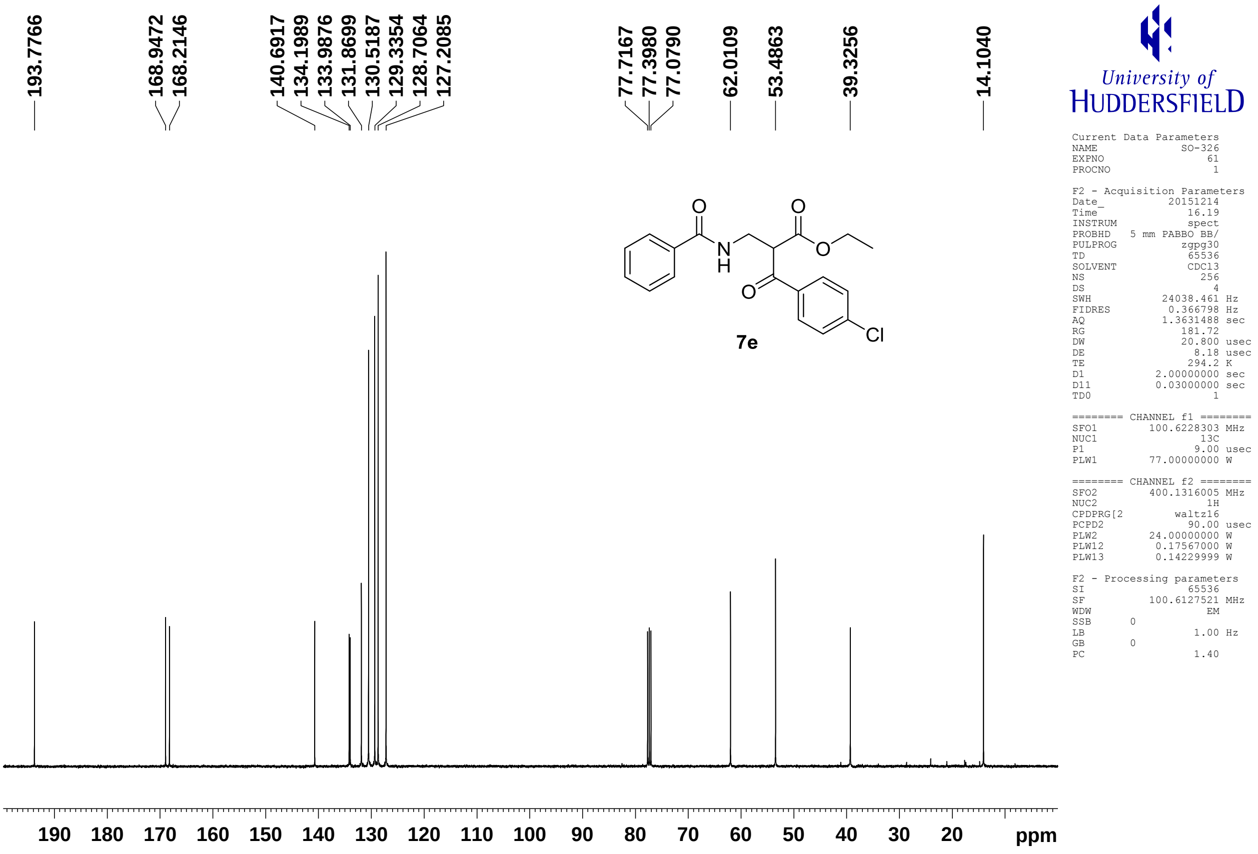
193.7766
168.9472
168.2146
140.6917
134.1989
133.9876
131.8699
130.5187
129.3354
128.7064
127.2085
77.7167
77.3980
77.0790
62.0109
53.4863
39.3256
14.1040
7e
Cl
190 180 170 160 150 140 130 120 110 100 90 80 70 60 50 40 30 20 ppm
University of HUDDERSFIELD
Current Data Parameters
NAME SO-326
EXPNO 61
PROCNO 1
F2 - Acquisition Parameters
Date_ 20151214
Time 16.19
INSTRUM spect
PROBHD 5 mm PABBO BB/
PULPROG zgpg30
TD 65536
SOLVENT CDCl3
NS 256
DS 4
SWH 24038.461 Hz
FIDRES 0.366798 Hz
AQ 1.3631488 sec
RG 181.72
DW 20.800 usec
DE 8.18 usec
TE 294.2 K
D1 2.00000000 sec
D11 0.03000000 sec
TD0 1
======== CHANNEL f1 ========
SFO1 100.6228303 MHz
NUC1 13C
P1 9.00 usec
PLW1 77.00000000 W
======== CHANNEL f2 ========
SFO2 400.1316005 MHz
NUC2 1H
CPDPRG[2 waltz16
PCPD2 90.00 usec
PLW2 24.00000000 W
PLW12 0.17567000 W
PLW13 0.14229999 W
F2 - Processing parameters
SI 65536
SF 100.6127521 MHz
WDW EM
SSB 0
LB 1.00 Hz
GB 0
PC 1.40

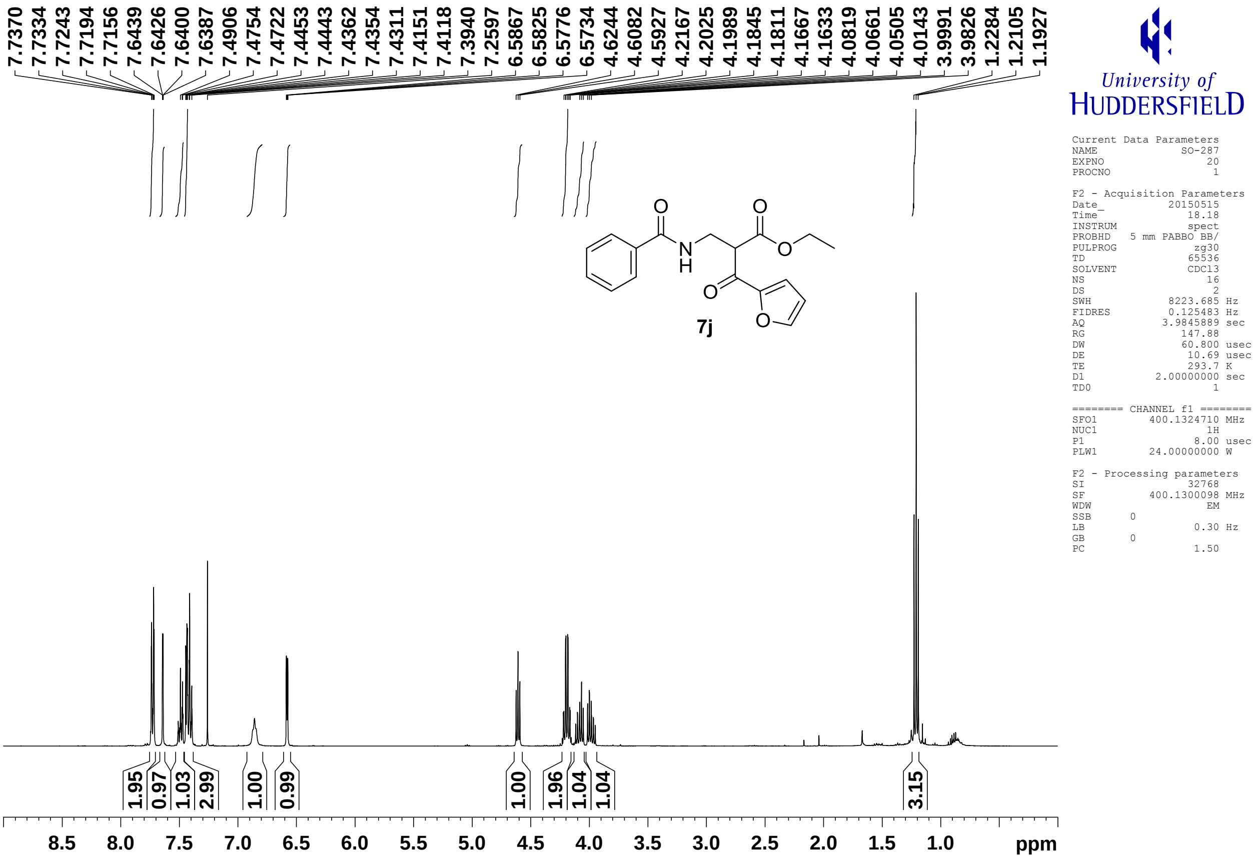
7.7370 7.7334 7.7243 7.7194 7.7156 7.6439 7.6426 7.6400 7.6387 7.4906 7.4754 7.4722 7.4453 7.4443 7.4362 7.4354 7.4311 7.4151 7.4118 7.3940 7.2597 6.5867 6.5825 6.5776 6.5734 4.6244 4.6082 4.5927 4.2167 4.2025 4.1989 4.1845 4.1811 4.1667 4.1633 4.0819 4.0661 4.0505 4.0143 3.9991 3.9826 1.2284 1.2105 1.1927
University of HUDDERSFIELD
Current Data Parameters
NAME SO-287
EXPNO 20
PROCNO 1
F2 - Acquisition Parameters
Date_ 20150515
Time 18.18
INSTRUM spect
PROBHD 5 mm PABBO BB/
PULPROG zg30
TD 65536
SOLVENT CDCl3
NS 16
DS 2
SWH 8223.685 Hz
FIDRES 0.125483 Hz
AQ 3.9845889 sec
RG 147.88
DW 60.800 usec
DE 10.69 usec
TE 293.7 K
D1 2.00000000 sec
TD0 1
======== CHANNEL f1 ========
SFO1 400.1324710 MHz
NUC1 1H
P1 8.00 usec
PLW1 24.00000000 W
F2 - Processing parameters
SI 32768
SF 400.1300098 MHz
WDW EM
SSB 0
LB 0.30 Hz
GB 0
PC 1.50
O
O
N
H
O
O
O
7j
1.95 0.97 1.03 2.99 1.00 0.99 1.00 1.96 1.04 1.04 3.15
8.5 8.0 7.5 7.0 6.5 6.0 5.5 5.0 4.5 4.0 3.5 3.0 2.5 2.0 1.5 1.0 ppm

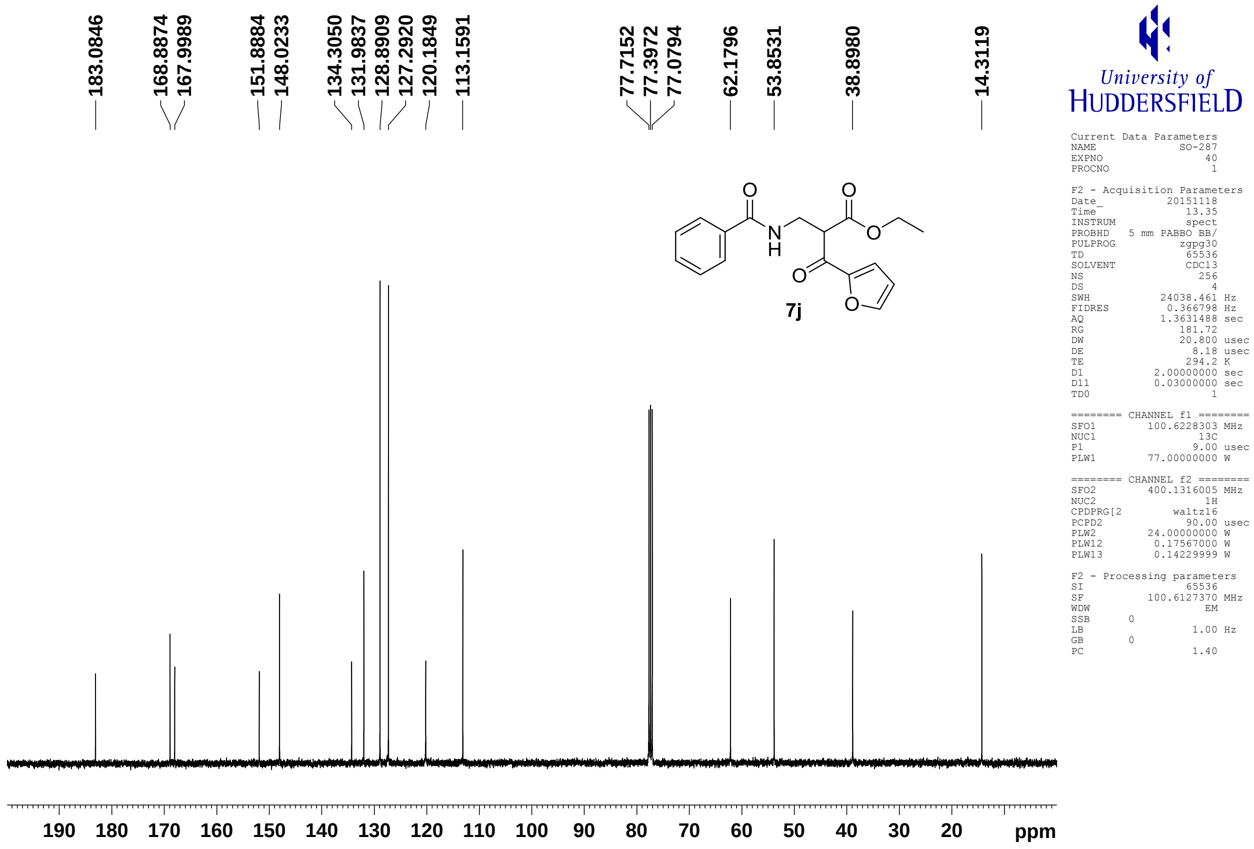
183.0846 168.8874 167.9989 151.8884 148.0233 134.3050 131.9837 128.8909 127.2920 120.1849 113.1591 77.7152 77.3972 77.0794 62.1796 53.8531 38.8980 14.3119
University of HUDDERSFIELD
Current Data Parameters
NAME SO-287
EXPNO 40
PROCNO 1
F2 - Acquisition Parameters
Date_ 20151118
Time 13.35
INSTRUM spect
PROBHD 5 mm PABBO BB/
PULPROG zgpg30
TD 65536
SOLVENT CDCl3
NS 256
DS 4
SWH 24038.461 Hz
FIDRES 0.366798 Hz
AQ 1.3631488 sec
RG 181.72
DW 20.800 usec
DE 8.18 usec
TE 294.2 K
D1 2.00000000 sec
D11 0.03000000 sec
TD0 1
======== CHANNEL f1 ========
SFO1 100.6228303 MHz
NUC1 13C
P1 9.00 usec
PLW1 77.00000000 W
======== CHANNEL f2 ========
SFO2 400.1316005 MHz
NUC2 1H
CPDPRG[2 waltz16
PCPD2 90.00 usec
PLW2 24.00000000 W
PLW12 0.17567000 W
PLW13 0.14229999 W
F2 - Processing parameters
SI 65536
SF 100.6127370 MHz
WDW EM
SSB 0
LB 1.00 Hz
GB 0
PC 1.40
O
O
N
H
O
O
O
7j
190 180 170 160 150 140 130 120 110 100 90 80 70 60 50 40 30 20 ppm

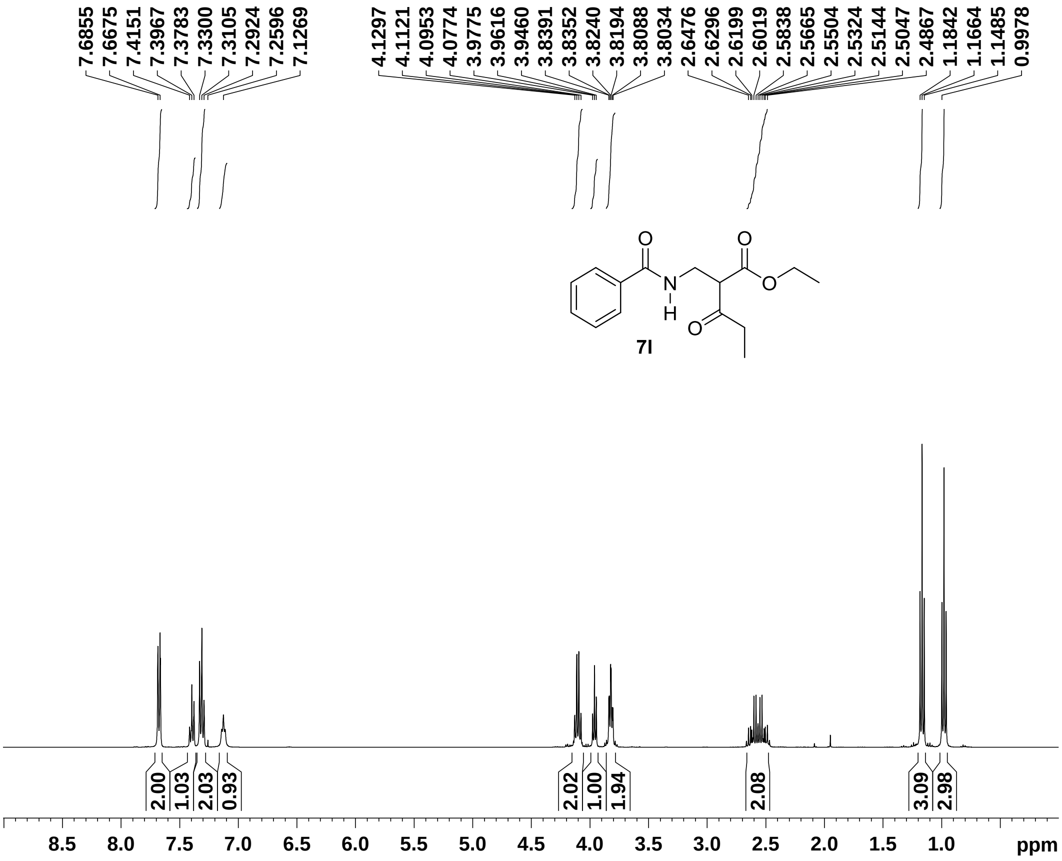

University of HUDDERSFIELD

```
Current Data Parameters
NAME              SO-440
EXPNO                 20
PROCNO                 1

F2 - Acquisition Parameters
Date_           20160112
Time               13.32
INSTRUM            spect
PROBHD   5 mm PABBO BB/
PULPROG             zg30
TD                 65536
SOLVENT            CDCl3
NS                    16
DS                     2
SWH             8223.685 Hz
FIDRES          0.125483 Hz
AQ             3.9845889 sec
RG                  15.2
DW                60.800 usec
DE                 10.69 usec
TE                 293.6 K
D1            2.00000000 sec
TD0                    1

======== CHANNEL f1 ========
SFO1         400.1324710 MHz
NUC1                  1H
P1                  8.00 usec
PLW1         24.00000000 W

F2 - Processing parameters
SI                 32768
SF           400.1300092 MHz
WDW                   EM
SSB      0
LB                  0.30 Hz
GB       0
PC                  1.50
```

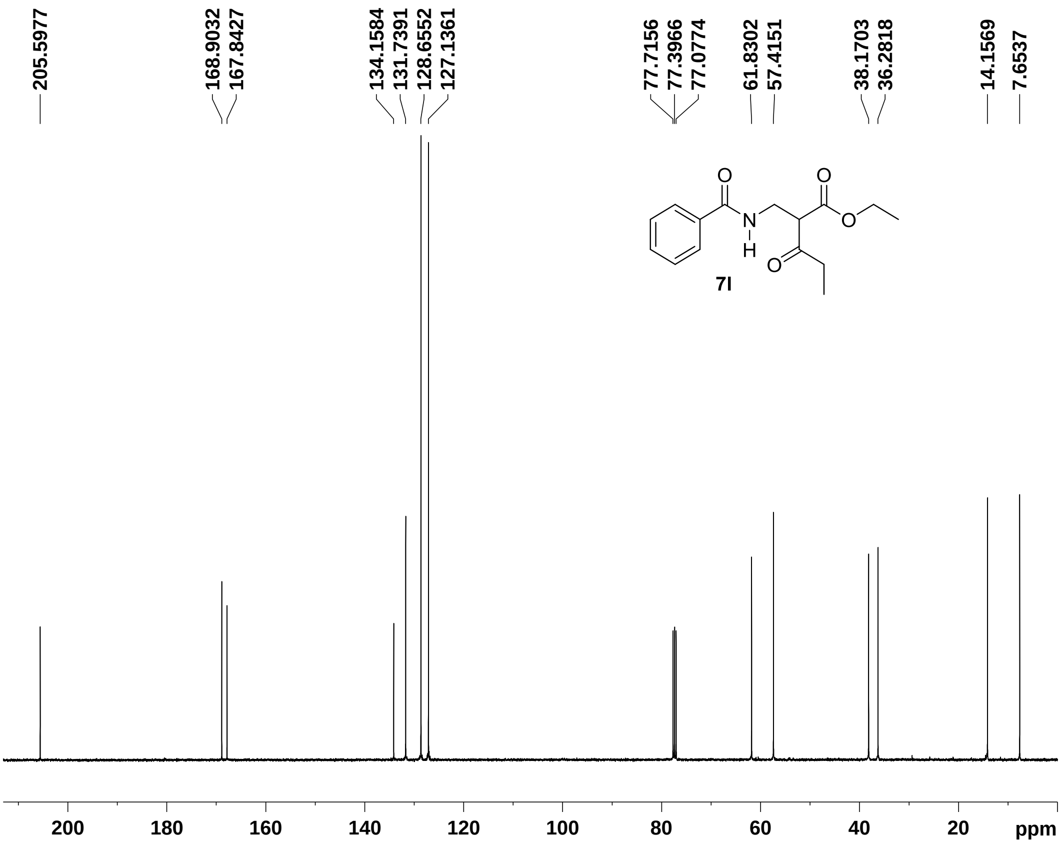

University of HUDDERSFIELD

```
Current Data Parameters
NAME              SO-440
EXPNO                 21
PROCNO                 1

F2 - Acquisition Parameters
Date_           20160112
Time               13.48
INSTRUM            spect
PROBHD   5 mm PABBO BB/
PULPROG          zgpg30
TD                 65536
SOLVENT            CDCl3
NS                   256
DS                     4
SWH            24038.461 Hz
FIDRES          0.366798 Hz
AQ             1.3631488 sec
RG                181.72
DW                20.800 usec
DE                  8.18 usec
TE                 294.0 K
D1            2.00000000 sec
D11           0.03000000 sec
TD0                    1

======== CHANNEL f1 ========
SFO1         100.6228303 MHz
NUC1                 13C
P1                  9.00 usec
PLW1         77.00000000 W

======== CHANNEL f2 ========
SFO2         400.1316005 MHz
NUC2                  1H
CPDPRG[2           waltz16
PCPD2              90.00 usec
PLW2         24.00000000 W
PLW12         0.17567000 W
PLW13         0.14229999 W

F2 - Processing parameters
SI                 65536
SF           100.6127512 MHz
WDW                   EM
SSB      0
LB                  1.00 Hz
GB       0
PC                  1.40
```

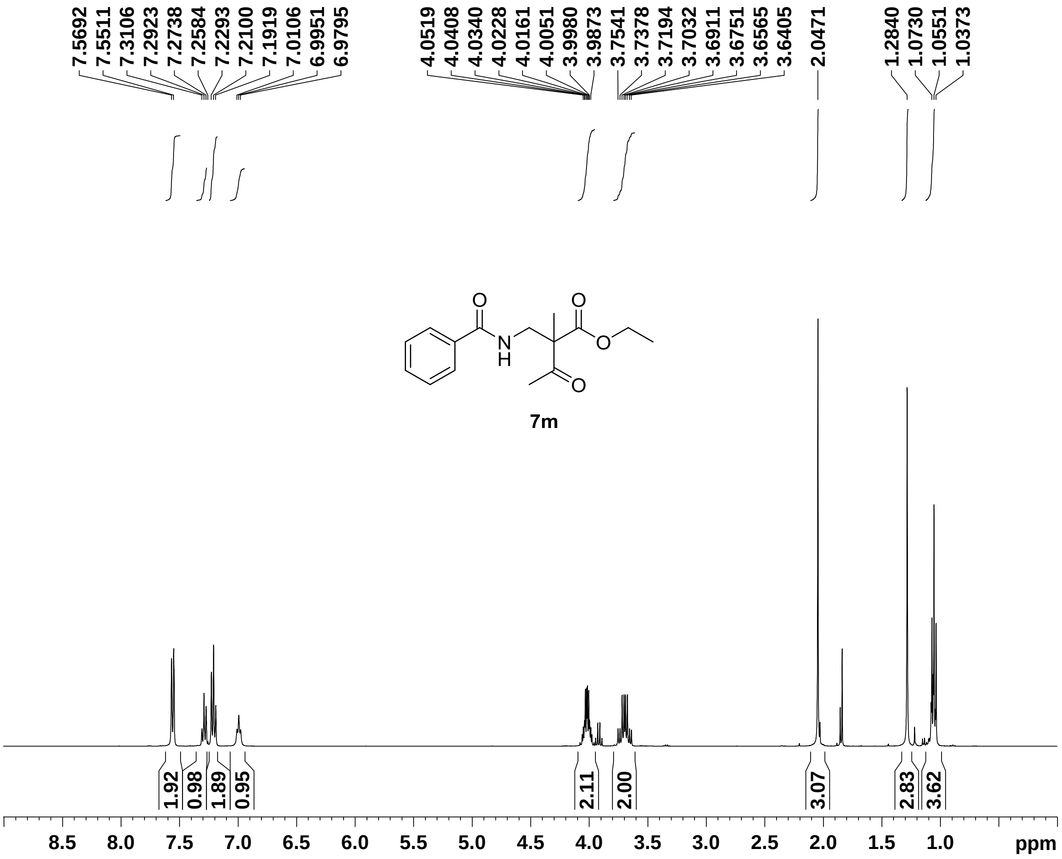

University of HUDDERSFIELD

Current Data Parameters
NAME SO-404
EXPNO 20
PROCNO 1

F2 - Acquisition Parameters
Date_ 20151031
Time 17.04
INSTRUM spect
PROBHD 5 mm PABBO BB/
PULPROG zg30
TD 65536
SOLVENT CDCl3
NS 16
DS 2
SWH 8223.685 Hz
FIDRES 0.125483 Hz
AQ 3.9845889 sec
RG 8.63
DW 60.800 usec
DE 10.69 usec
TE 294.1 K
D1 2.00000000 sec
TD0 1

======== CHANNEL f1 ========
SFO1 400.1324710 MHz
NUC1 1H
P1 8.00 usec
PLW1 24.00000000 W

F2 - Processing parameters
SI 32768
SF 400.1300090 MHz
WDW EM
SSB 0
LB 0.30 Hz
GB 0
PC 1.50

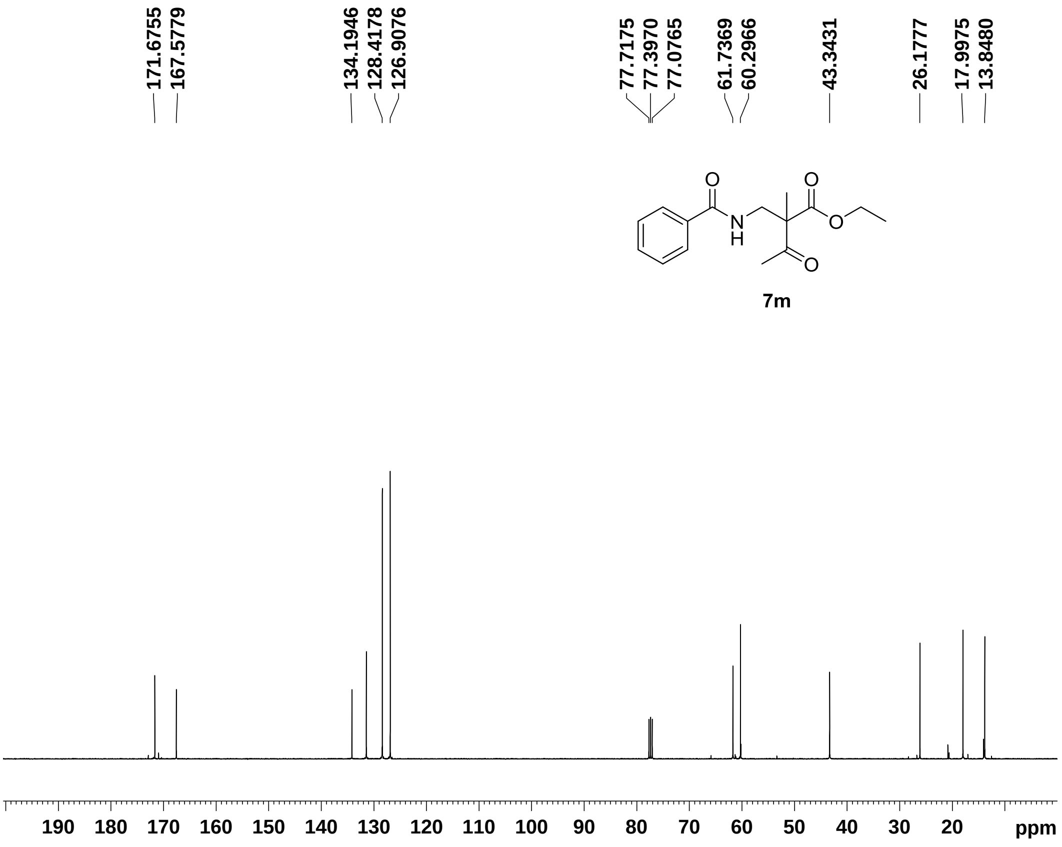

University of HUDDERSFIELD

Current Data Parameters
NAME SO-404
EXPNO 21
PROCNO 1

F2 - Acquisition Parameters
Date_ 20151031
Time 17.20
INSTRUM spect
PROBHD 5 mm PABBO BB/
PULPROG zgpg30
TD 65536
SOLVENT CDCl3
NS 256
DS 4
SWH 24038.461 Hz
FIDRES 0.366798 Hz
AQ 1.3631488 sec
RG 181.72
DW 20.800 usec
DE 8.18 usec
TE 294.4 K
D1 2.00000000 sec
D11 0.03000000 sec
TD0 1

======== CHANNEL f1 ========
SFO1 100.6228303 MHz
NUC1 13C
P1 9.00 usec
PLW1 77.00000000 W

======== CHANNEL f2 ========
SFO2 400.1316005 MHz
NUC2 1H
CPDPRG[2 waltz16
PCPD2 90.00 usec
PLW2 24.00000000 W
PLW12 0.17567000 W
PLW13 0.14229999 W

F2 - Processing parameters
SI 65536
SF 100.6127642 MHz
WDW EM
SSB 0
LB 1.00 Hz
GB 0
PC 1.40

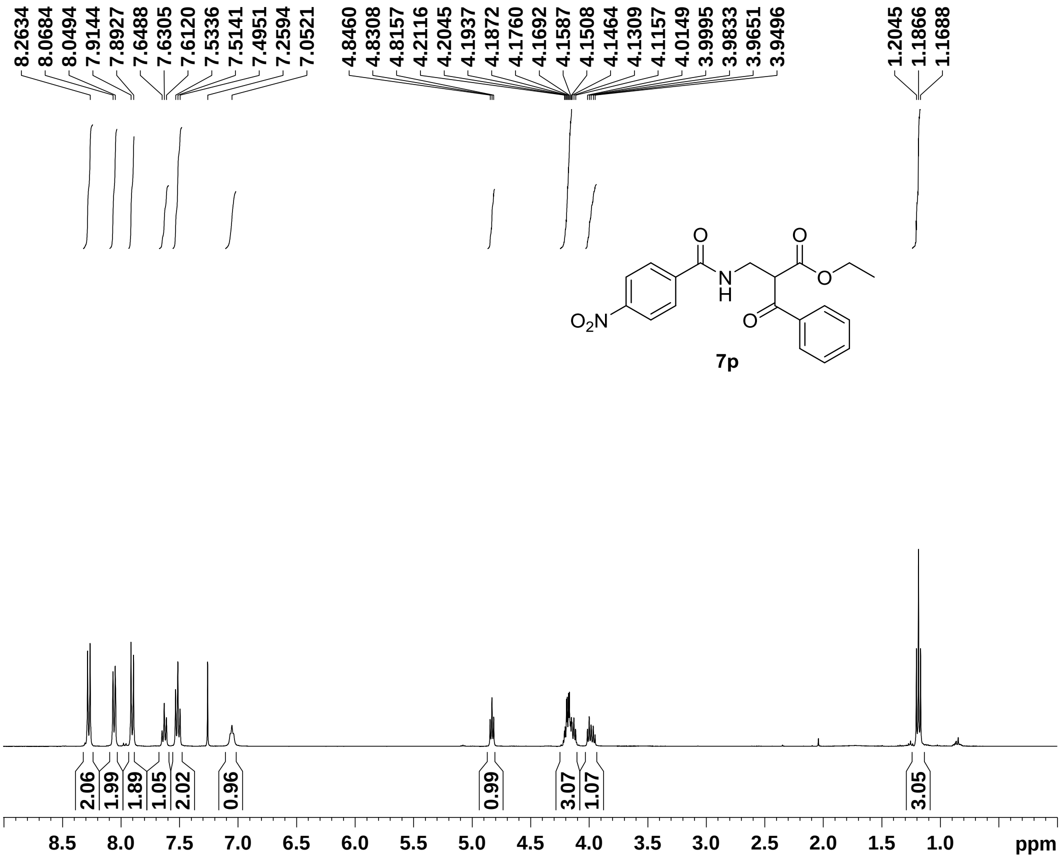

University of
HUDDERSFIELD

Current Data Parameters
NAME SO-310
EXPNO 20
PROCNO 1

F2 - Acquisition Parameters
Date_ 20150616
Time 13.25
INSTRUM spect
PROBHD 5 mm PABBO BB/
PULPROG zg30
TD 65536
SOLVENT CDCl3
NS 16
DS 2
SWH 8223.685 Hz
FIDRES 0.125483 Hz
AQ 3.9845889 sec
RG 147.88
DW 60.800 usec
DE 10.69 usec
TE 293.9 K
D1 2.00000000 sec
TD0 1

======== CHANNEL f1 ========
SFO1 400.1324710 MHz
NUC1 1H
P1 8.00 usec
PLW1 24.00000000 W

F2 - Processing parameters
SI 32768
SF 400.1300098 MHz
WDW EM
SSB 0
LB 0.30 Hz
GB 0
PC 1.50

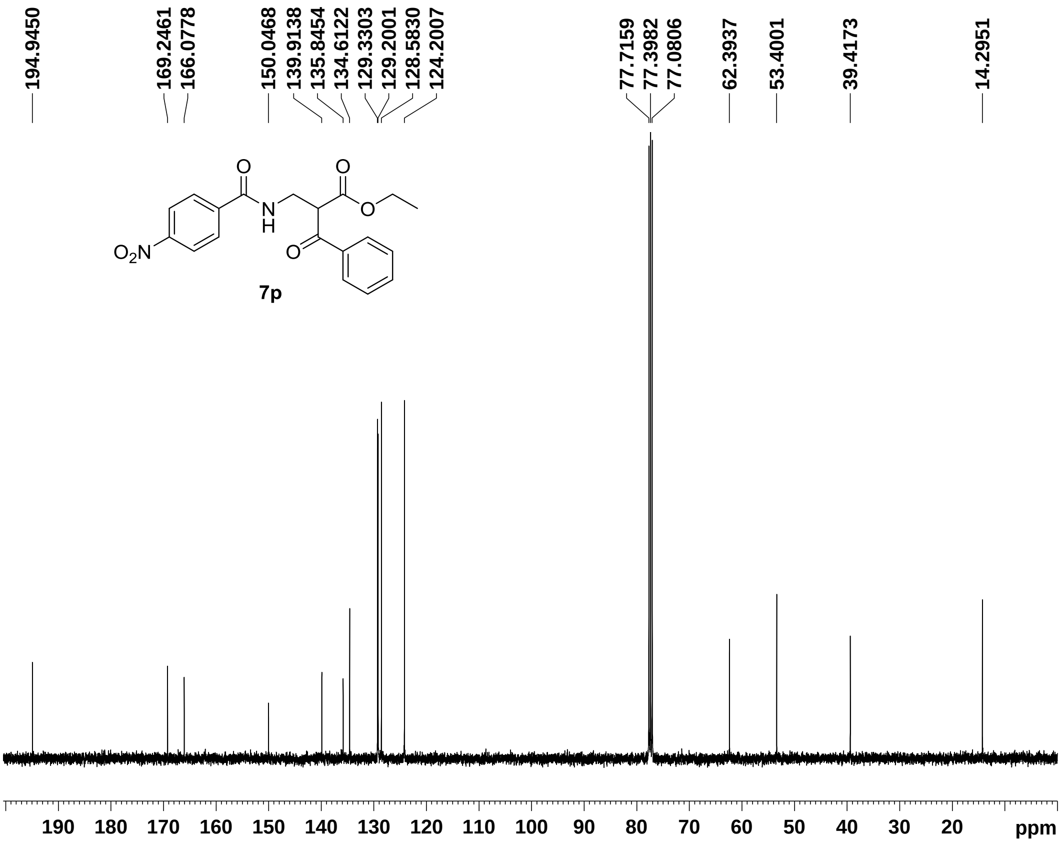

University of
HUDDERSFIELD

Current Data Parameters
NAME SO-310
EXPNO 40
PROCNO 1

F2 - Acquisition Parameters
Date_ 20151104
Time 6.53
INSTRUM spect
PROBHD 5 mm PABBO BB/
PULPROG zgpg30
TD 65536
SOLVENT CDCl3
NS 256
DS 4
SWH 24038.461 Hz
FIDRES 0.366798 Hz
AQ 1.3631488 sec
RG 181.72
DW 20.800 usec
DE 8.18 usec
TE 294.3 K
D1 2.00000000 sec
D11 0.03000000 sec
TD0 1

======== CHANNEL f1 ========
SFO1 100.6228303 MHz
NUC1 13C
P1 9.00 usec
PLW1 77.00000000 W

======== CHANNEL f2 ========
SFO2 400.1316005 MHz
NUC2 1H
CPDPRG[2 waltz16
PCPD2 90.00 usec
PLW2 24.00000000 W
PLW12 0.17567000 W
PLW13 0.14229999 W

F2 - Processing parameters
SI 65536
SF 100.6127339 MHz
WDW EM
SSB 0
LB 1.00 Hz
GB 0
PC 1.40

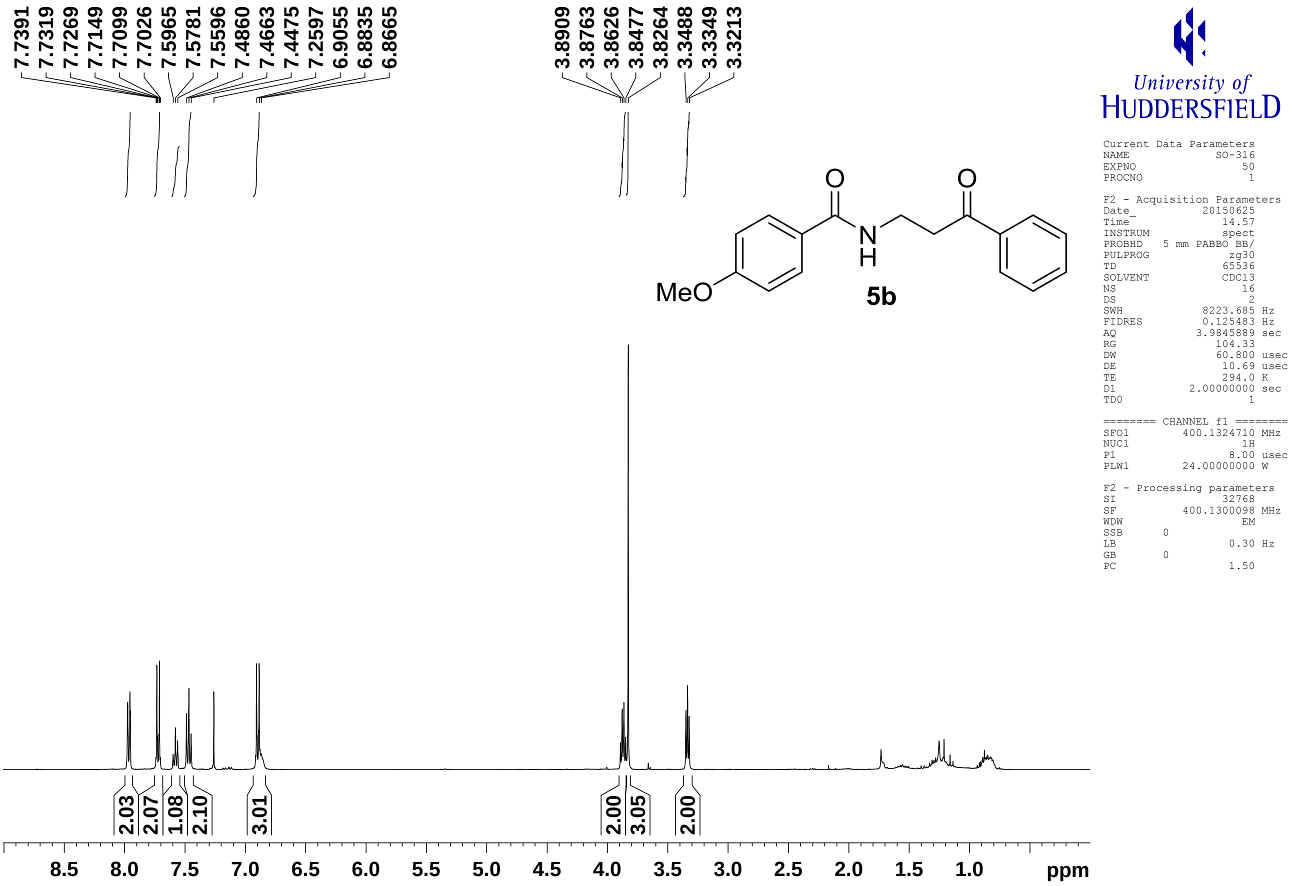
7.7391 7.7319 7.7269 7.7149 7.7099 7.7026 7.5965 7.5781 7.5596 7.4860 7.4663 7.4475 7.2597 6.9055 6.8835 6.8665
3.8909 3.8763 3.8626 3.8477 3.8264 3.3488 3.3349 3.3213
University of HUDDERSFIELD
Current Data Parameters
NAME SO-316
EXPNO 50
PROCNO 1
F2 - Acquisition Parameters
Date_ 20150625
Time 14.57
INSTRUM spect
PROBHD 5 mm PABBO BB/
PULPROG zg30
TD 65536
SOLVENT CDCl3
NS 16
DS 2
SWH 8223.685 Hz
FIDRES 0.125483 Hz
AQ 3.9845889 sec
RG 104.33
DW 60.800 usec
DE 10.69 usec
TE 294.0 K
D1 2.00000000 sec
TD0 1
======== CHANNEL f1 ========
SFO1 400.1324710 MHz
NUC1 1H
P1 8.00 usec
PLW1 24.00000000 W
F2 - Processing parameters
SI 32768
SF 400.1300098 MHz
WDW EM
SSB 0
LB 0.30 Hz
GB 0
PC 1.50
MeO
O
N
H
O
5b
2.03 2.07 1.08 2.10 3.01 2.00 3.05 2.00
8.5 8.0 7.5 7.0 6.5 6.0 5.5 5.0 4.5 4.0 3.5 3.0 2.5 2.0 1.5 1.0 ppm

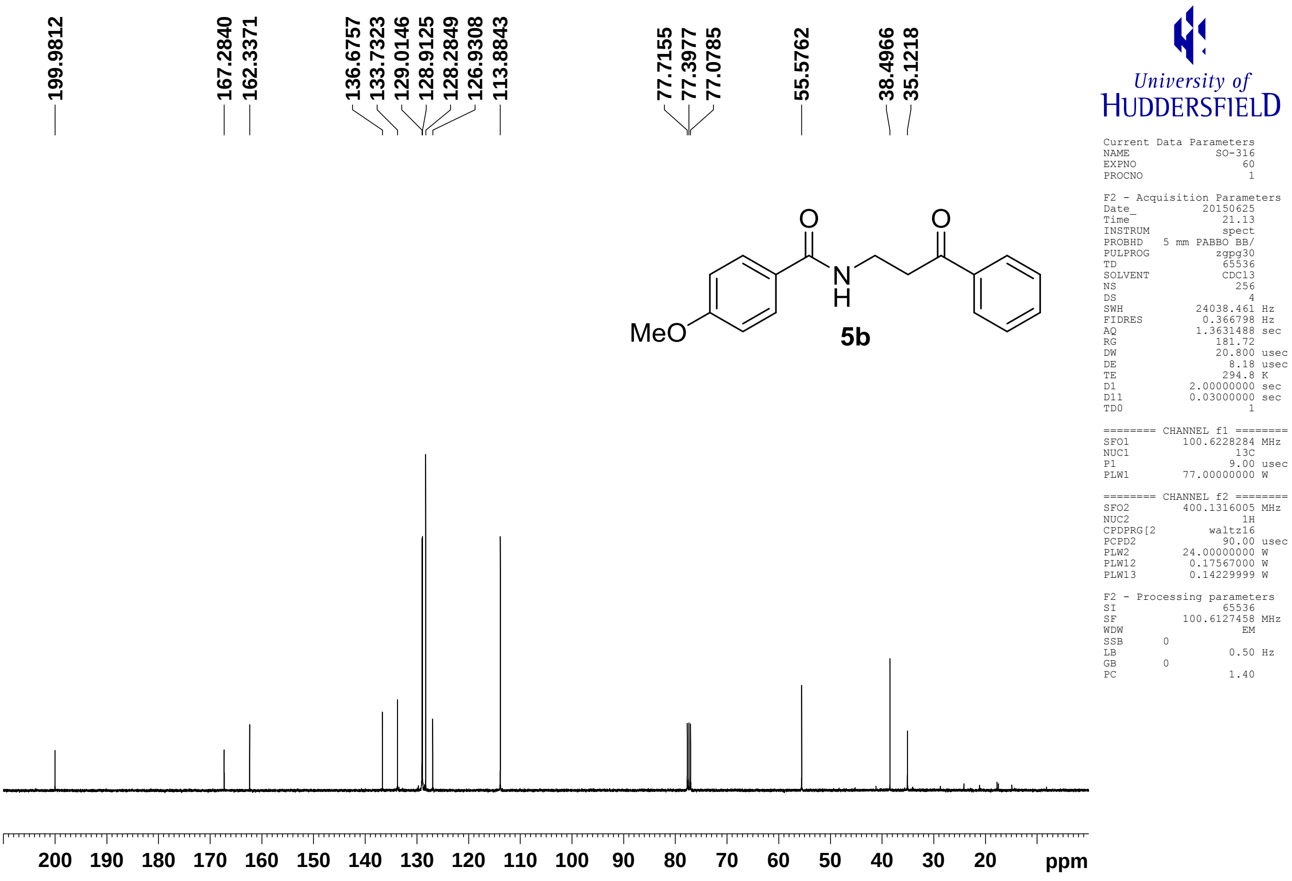
199.9812
167.2840 162.3371
136.6757 133.7323 129.0146 128.9125 128.2849 126.9308 113.8843
77.7155 77.3977 77.0785
55.5762
38.4966 35.1218
University of HUDDERSFIELD
Current Data Parameters
NAME SO-316
EXPNO 60
PROCNO 1
F2 - Acquisition Parameters
Date_ 20150625
Time 21.13
INSTRUM spect
PROBHD 5 mm PABBO BB/
PULPROG zgpg30
TD 65536
SOLVENT CDCl3
NS 256
DS 4
SWH 24038.461 Hz
FIDRES 0.366798 Hz
AQ 1.3631488 sec
RG 181.72
DW 20.800 usec
DE 8.18 usec
TE 294.8 K
D1 2.00000000 sec
D11 0.03000000 sec
TD0 1
======== CHANNEL f1 ========
SFO1 100.6228284 MHz
NUC1 13C
P1 9.00 usec
PLW1 77.00000000 W
======== CHANNEL f2 ========
SFO2 400.1316005 MHz
NUC2 1H
CPDPRG[2 waltz16
PCPD2 90.00 usec
PLW2 24.00000000 W
PLW12 0.17567000 W
PLW13 0.14229999 W
F2 - Processing parameters
SI 65536
SF 100.6127458 MHz
WDW EM
SSB 0
LB 0.50 Hz
GB 0
PC 1.40
MeO
O
N
H
O
5b
200 190 180 170 160 150 140 130 120 110 100 90 80 70 60 50 40 30 20 ppm

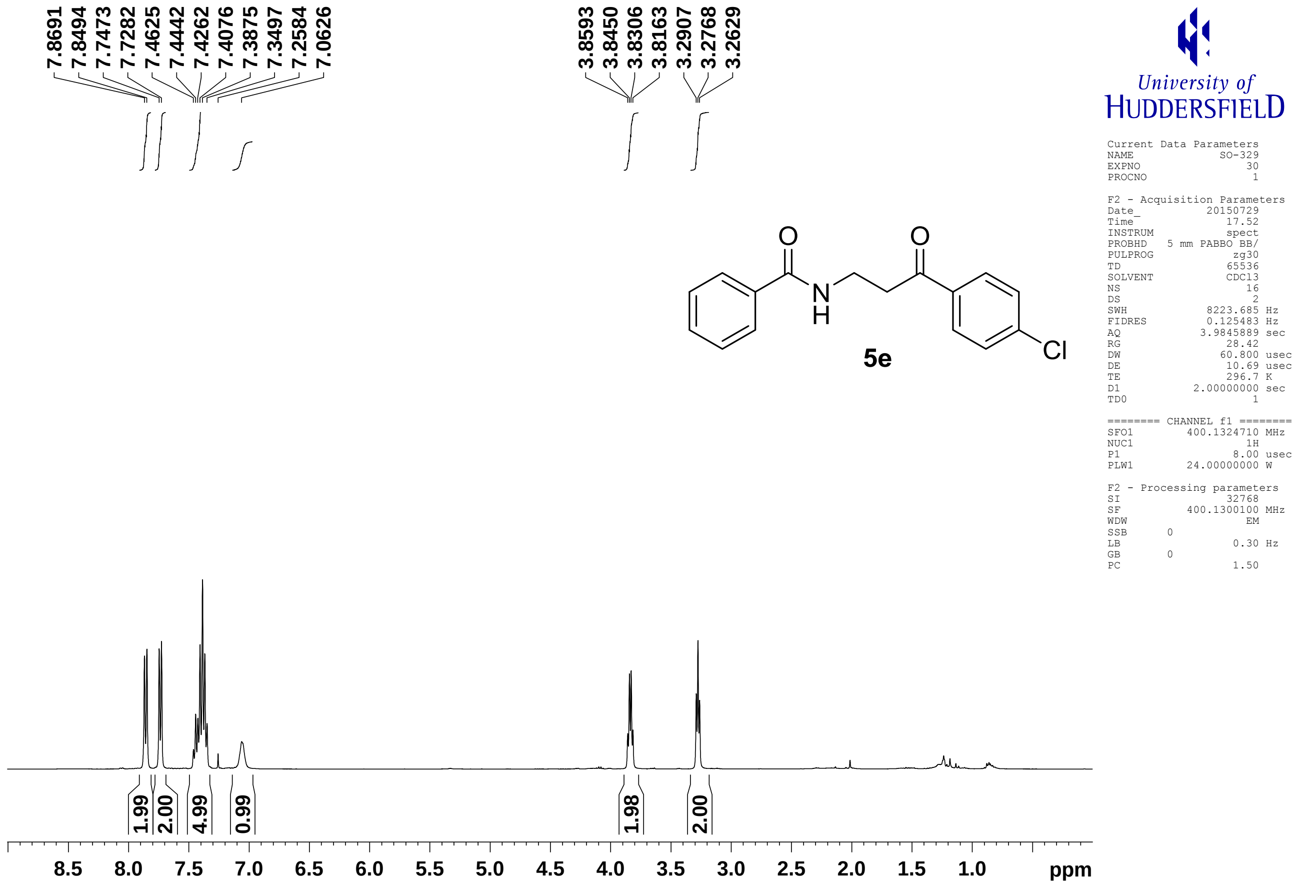
7.8691
7.8494
7.7473
7.7282
7.4625
7.4442
7.4262
7.4076
7.3875
7.3497
7.2584
7.0626
3.8593
3.8450
3.8306
3.8163
3.2907
3.2768
3.2629
University of HUDDERSFIELD
Current Data Parameters
NAME SO-329
EXPNO 30
PROCNO 1
F2 - Acquisition Parameters
Date_ 20150729
Time 17.52
INSTRUM spect
PROBHD 5 mm PABBO BB/
PULPROG zg30
TD 65536
SOLVENT CDCl3
NS 16
DS 2
SWH 8223.685 Hz
FIDRES 0.125483 Hz
AQ 3.9845889 sec
RG 28.42
DW 60.800 usec
DE 10.69 usec
TE 296.7 K
D1 2.00000000 sec
TD0 1
======== CHANNEL f1 ========
SFO1 400.1324710 MHz
NUC1 1H
P1 8.00 usec
PLW1 24.00000000 W
F2 - Processing parameters
SI 32768
SF 400.1300100 MHz
WDW EM
SSB 0
LB 0.30 Hz
GB 0
PC 1.50
O
O
N
H
Cl
5e
1.99
2.00
4.99
0.99
1.98
2.00
8.5
8.0
7.5
7.0
6.5
6.0
5.5
5.0
4.5
4.0
3.5
3.0
2.5
2.0
1.5
1.0
ppm

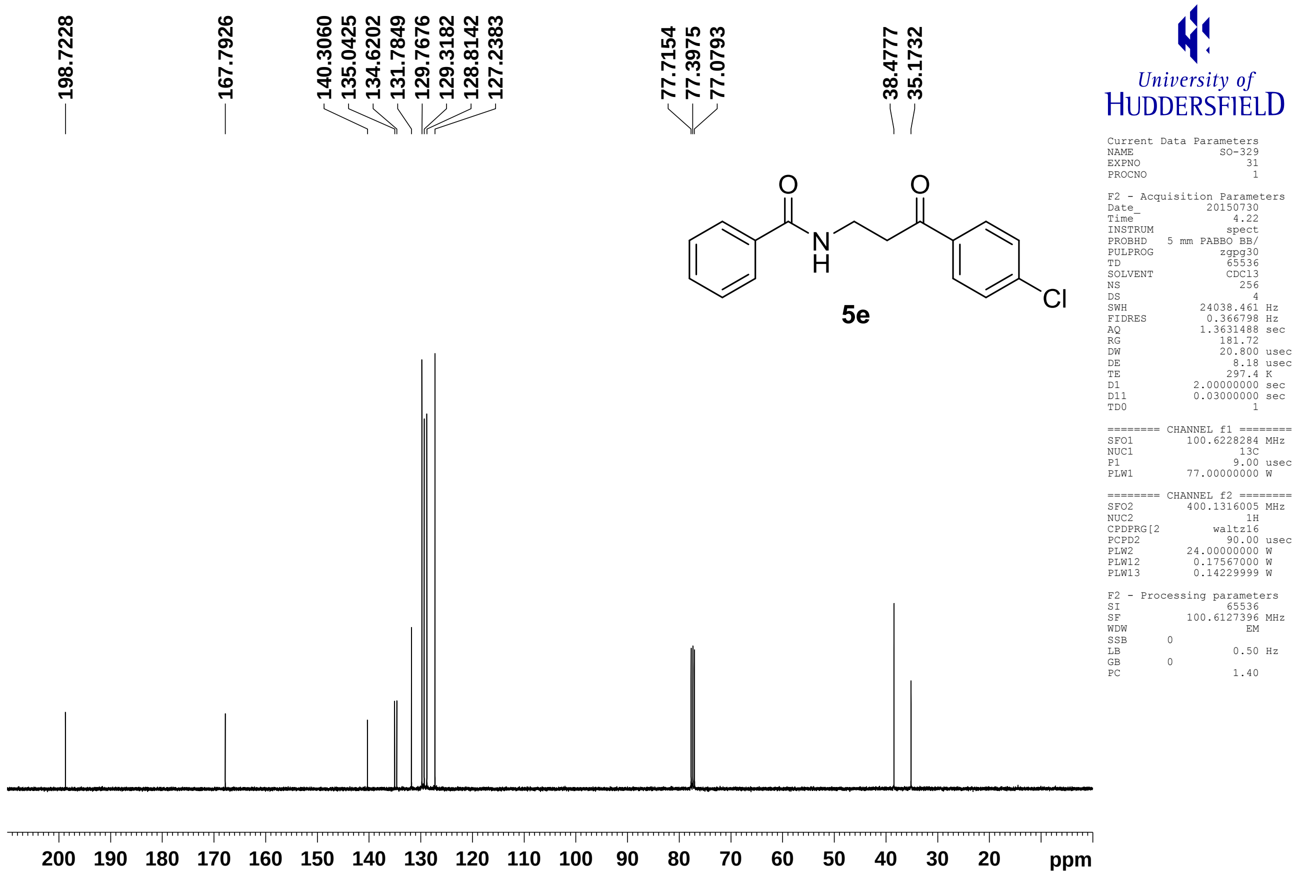
198.7228
167.7926
140.3060
135.0425
134.6202
131.7849
129.7676
129.3182
128.8142
127.2383
77.7154
77.3975
77.0793
38.4777
35.1732
University of HUDDERSFIELD
Current Data Parameters
NAME SO-329
EXPNO 31
PROCNO 1
F2 - Acquisition Parameters
Date_ 20150730
Time 4.22
INSTRUM spect
PROBHD 5 mm PABBO BB/
PULPROG zgpg30
TD 65536
SOLVENT CDCl3
NS 256
DS 4
SWH 24038.461 Hz
FIDRES 0.366798 Hz
AQ 1.3631488 sec
RG 181.72
DW 20.800 usec
DE 8.18 usec
TE 297.4 K
D1 2.00000000 sec
D11 0.03000000 sec
TD0 1
======== CHANNEL f1 ========
SFO1 100.6228284 MHz
NUC1 13C
P1 9.00 usec
PLW1 77.00000000 W
======== CHANNEL f2 ========
SFO2 400.1316005 MHz
NUC2 1H
CPDPRG[2 waltz16
PCPD2 90.00 usec
PLW2 24.00000000 W
PLW12 0.17567000 W
PLW13 0.14229999 W
F2 - Processing parameters
SI 65536
SF 100.6127396 MHz
WDW EM
SSB 0
LB 0.50 Hz
GB 0
PC 1.40
O
O
N
H
Cl
5e
200
190
180
170
160
150
140
130
120
110
100
90
80
70
60
50
40
30
20
ppm

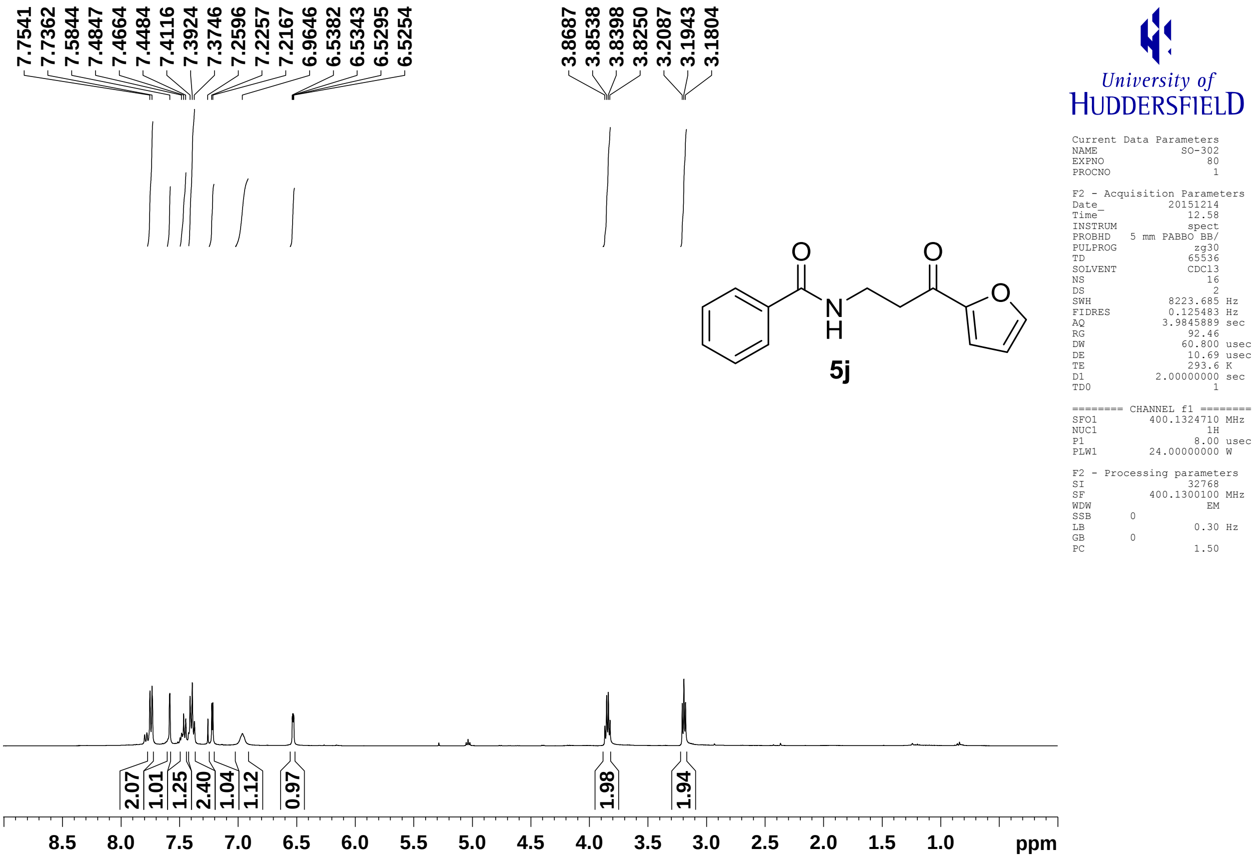
7.7541 7.7362 7.5844 7.4847 7.4664 7.4484 7.4116 7.3924 7.3746 7.2596 7.2257 7.2167 6.9646 6.5382 6.5343 6.5295 6.5254
3.8687 3.8538 3.8398 3.8250 3.2087 3.1943 3.1804
O
N
H
O
O
5j
2.07 1.01 1.25 2.40 1.04 1.12 0.97 1.98 1.94
8.5 8.0 7.5 7.0 6.5 6.0 5.5 5.0 4.5 4.0 3.5 3.0 2.5 2.0 1.5 1.0 ppm
University of HUDDERSFIELD
Current Data Parameters
NAME SO-302
EXPNO 80
PROCNO 1
F2 - Acquisition Parameters
Date_ 20151214
Time 12.58
INSTRUM spect
PROBHD 5 mm PABBO BB/
PULPROG zg30
TD 65536
SOLVENT CDCl3
NS 16
DS 2
SWH 8223.685 Hz
FIDRES 0.125483 Hz
AQ 3.9845889 sec
RG 92.46
DW 60.800 usec
DE 10.69 usec
TE 293.6 K
D1 2.00000000 sec
TD0 1
======== CHANNEL f1 ========
SFO1 400.1324710 MHz
NUC1 1H
P1 8.00 usec
PLW1 24.00000000 W
F2 - Processing parameters
SI 32768
SF 400.1300100 MHz
WDW EM
SSB 0
LB 0.30 Hz
GB 0
PC 1.50

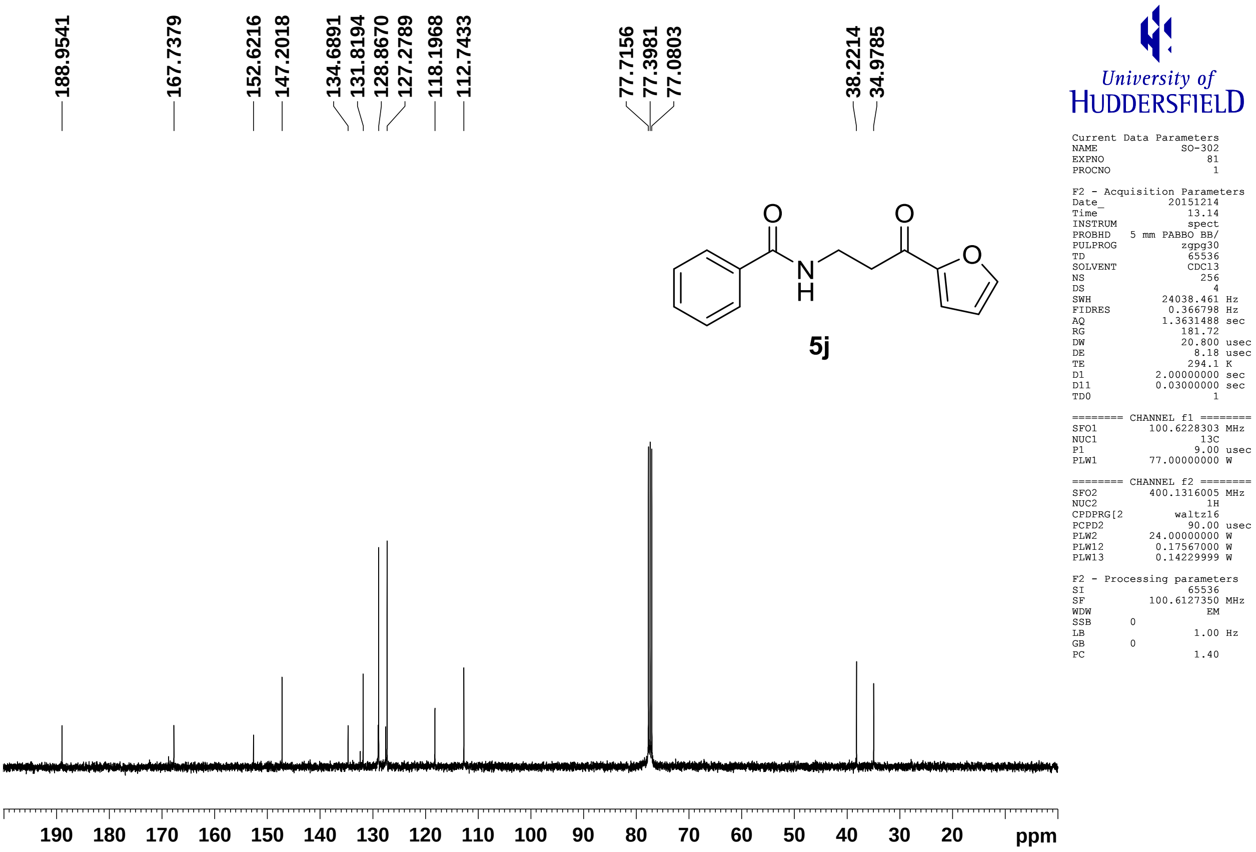
188.9541 167.7379 152.6216 147.2018 134.6891 131.8194 128.8670 127.2789 118.1968 112.7433
77.7156 77.3981 77.0803 38.2214 34.9785
O
N
H
O
O
5j
190 180 170 160 150 140 130 120 110 100 90 80 70 60 50 40 30 20 ppm
University of HUDDERSFIELD
Current Data Parameters
NAME SO-302
EXPNO 81
PROCNO 1
F2 - Acquisition Parameters
Date_ 20151214
Time 13.14
INSTRUM spect
PROBHD 5 mm PABBO BB/
PULPROG zgpg30
TD 65536
SOLVENT CDCl3
NS 256
DS 4
SWH 24038.461 Hz
FIDRES 0.366798 Hz
AQ 1.3631488 sec
RG 181.72
DW 20.800 usec
DE 8.18 usec
TE 294.1 K
D1 2.00000000 sec
D11 0.03000000 sec
TD0 1
======== CHANNEL f1 ========
SFO1 100.6228303 MHz
NUC1 13C
P1 9.00 usec
PLW1 77.00000000 W
======== CHANNEL f2 ========
SFO2 400.1316005 MHz
NUC2 1H
CPDPRG[2 waltz16
PCPD2 90.00 usec
PLW2 24.00000000 W
PLW12 0.17567000 W
PLW13 0.14229999 W
F2 - Processing parameters
SI 65536
SF 100.6127350 MHz
WDW EM
SSB 0
LB 1.00 Hz
GB 0
PC 1.40

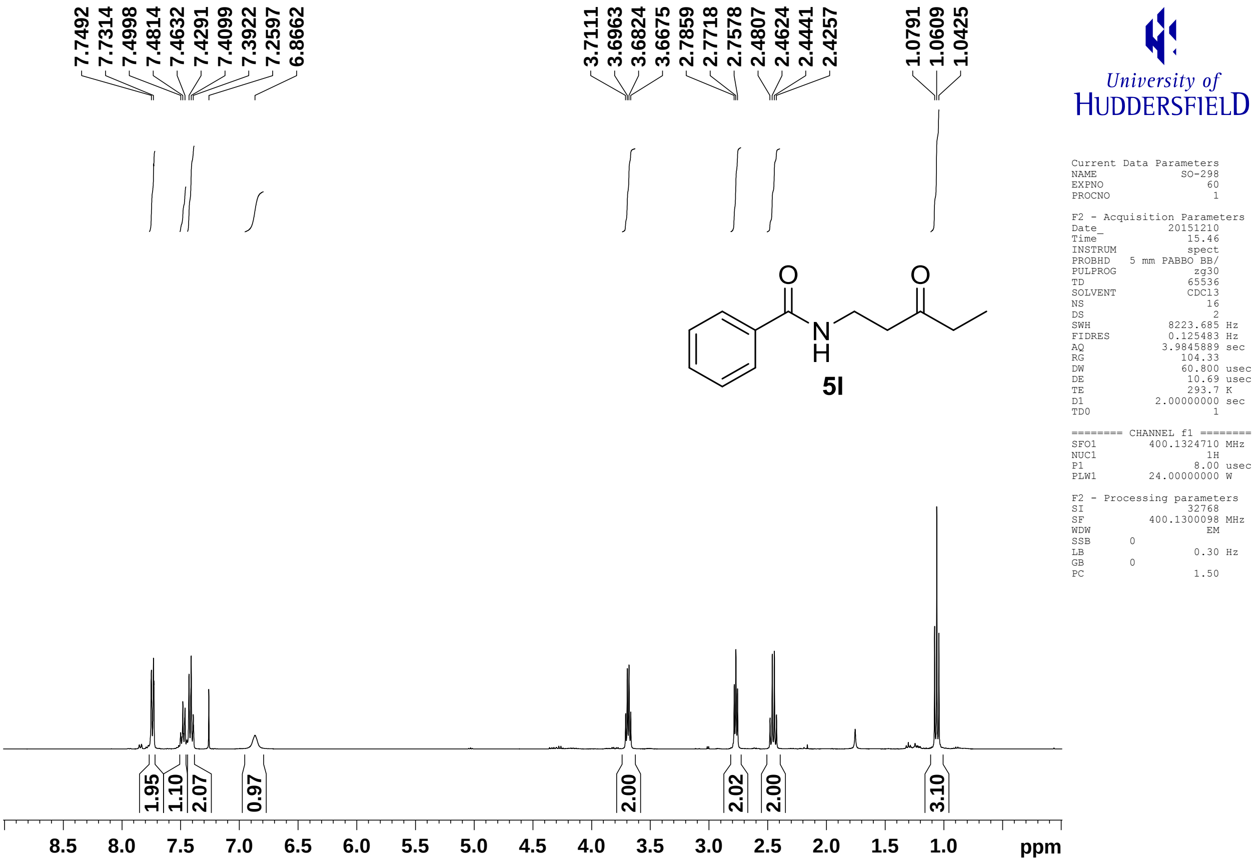
7.7492
7.7314
7.4998
7.4814
7.4632
7.4291
7.4099
7.3922
7.2597
6.8662
3.7111
3.6963
3.6824
3.6675
2.7859
2.7718
2.7578
2.4807
2.4624
2.4441
2.4257
1.0791
1.0609
1.0425
University of HUDDERSFIELD
Current Data Parameters
NAME SO-298
EXPNO 60
PROCNO 1
F2 - Acquisition Parameters
Date_ 20151210
Time 15.46
INSTRUM spect
PROBHD 5 mm PABBO BB/
PULPROG zg30
TD 65536
SOLVENT CDCl3
NS 16
DS 2
SWH 8223.685 Hz
FIDRES 0.125483 Hz
AQ 3.9845889 sec
RG 104.33
DW 60.800 usec
DE 10.69 usec
TE 293.7 K
D1 2.00000000 sec
TD0 1
======== CHANNEL f1 ========
SFO1 400.1324710 MHz
NUC1 1H
P1 8.00 usec
PLW1 24.00000000 W
F2 - Processing parameters
SI 32768
SF 400.1300098 MHz
WDW EM
SSB 0
LB 0.30 Hz
GB 0
PC 1.50
O
N
H
O
5l
1.95
1.10
2.07
0.97
2.00
2.02
2.00
3.10
8.5
8.0
7.5
7.0
6.5
6.0
5.5
5.0
4.5
4.0
3.5
3.0
2.5
2.0
1.5
1.0
ppm

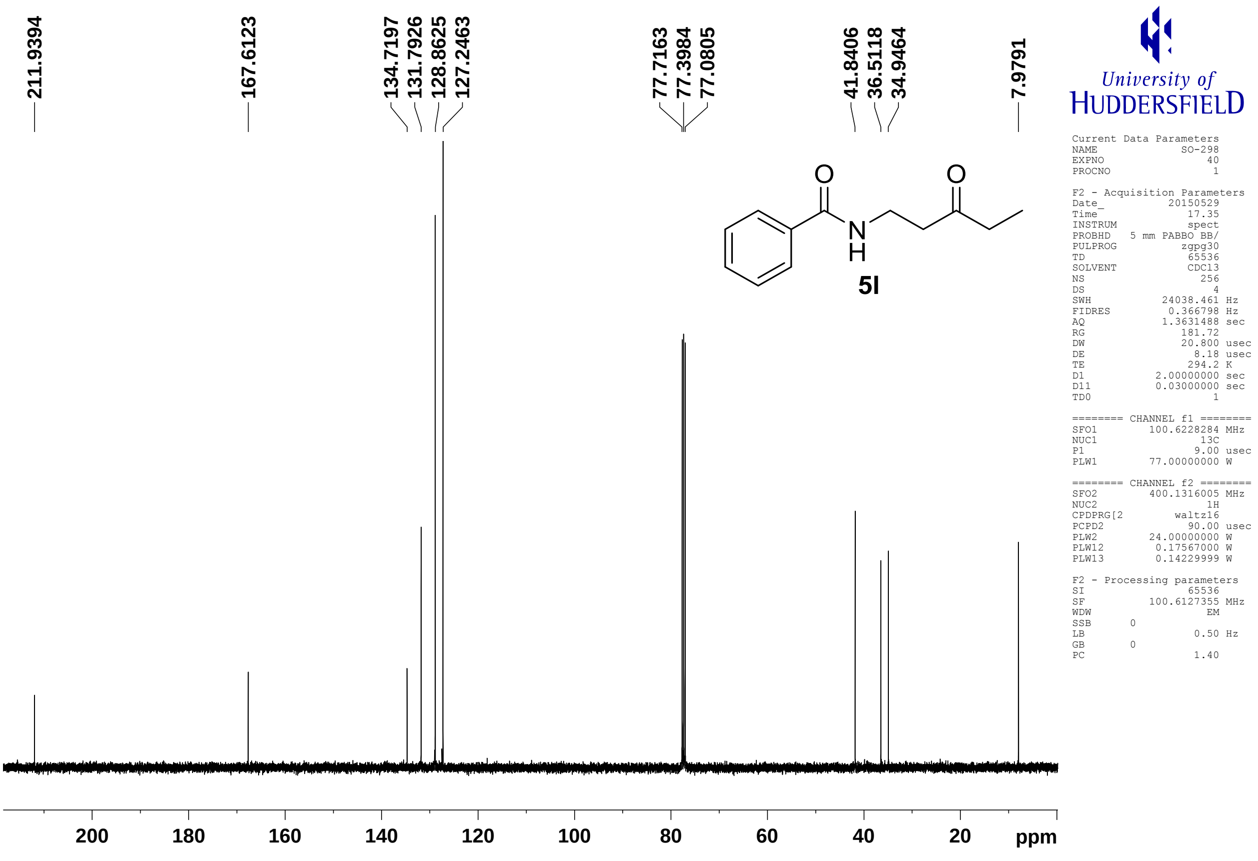
211.9394
167.6123
134.7197
131.7926
128.8625
127.2463
77.7163
77.3984
77.0805
41.8406
36.5118
34.9464
7.9791
University of HUDDERSFIELD
Current Data Parameters
NAME SO-298
EXPNO 40
PROCNO 1
F2 - Acquisition Parameters
Date_ 20150529
Time 17.35
INSTRUM spect
PROBHD 5 mm PABBO BB/
PULPROG zgpg30
TD 65536
SOLVENT CDCl3
NS 256
DS 4
SWH 24038.461 Hz
FIDRES 0.366798 Hz
AQ 1.3631488 sec
RG 181.72
DW 20.800 usec
DE 8.18 usec
TE 294.2 K
D1 2.00000000 sec
D11 0.03000000 sec
TD0 1
======== CHANNEL f1 ========
SFO1 100.6228284 MHz
NUC1 13C
P1 9.00 usec
PLW1 77.00000000 W
======== CHANNEL f2 ========
SFO2 400.1316005 MHz
NUC2 1H
CPDPRG[2 waltz16
PCPD2 90.00 usec
PLW2 24.00000000 W
PLW12 0.17567000 W
PLW13 0.14229999 W
F2 - Processing parameters
SI 65536
SF 100.6127355 MHz
WDW EM
SSB 0
LB 0.50 Hz
GB 0
PC 1.40
O
N
H
O
5l
200
180
160
140
120
100
80
60
40
20
ppm

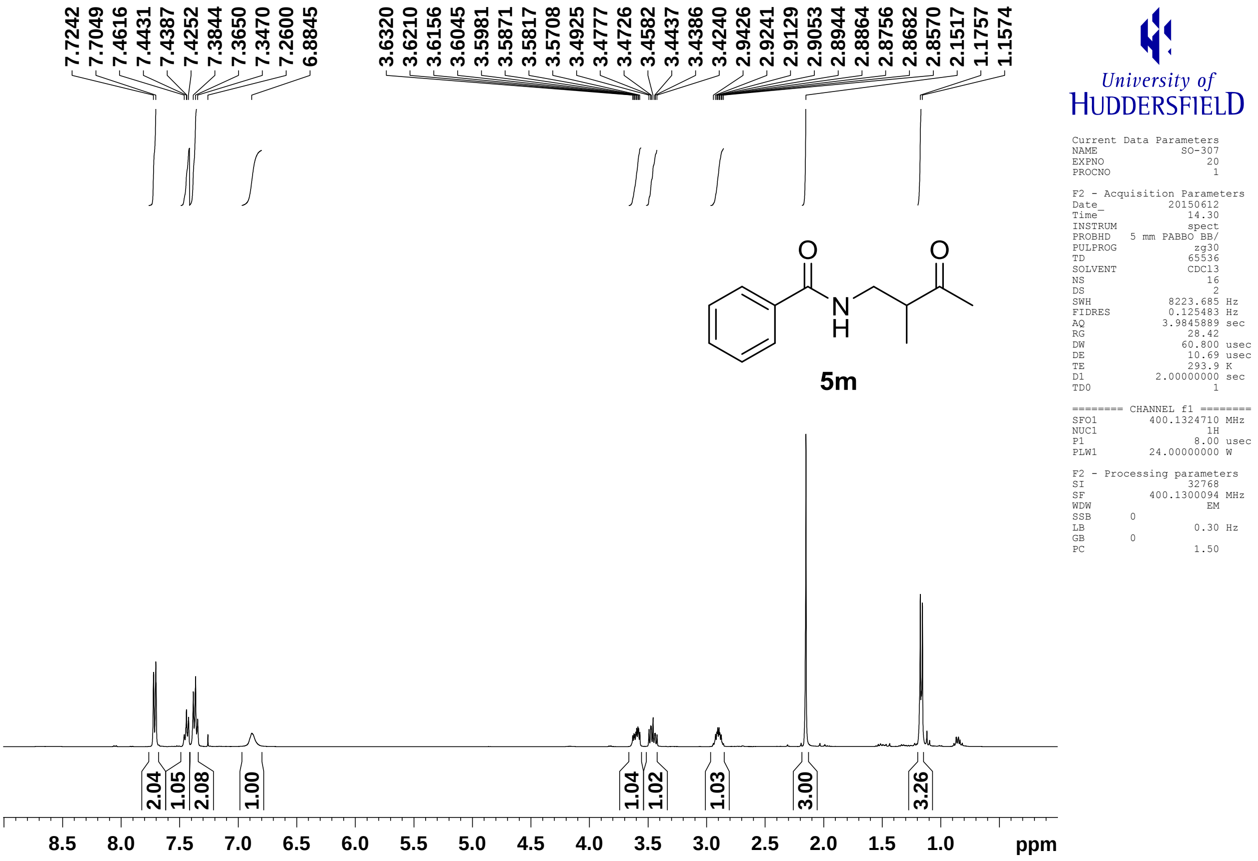
7.7242 7.7049 7.4616 7.4431 7.4387 7.4252 7.3844 7.3650 7.3470 7.2600 6.8845
3.6320 3.6210 3.6156 3.6045 3.5981 3.5871 3.5817 3.5708 3.4925 3.4777 3.4726 3.4582 3.4437 3.4386 3.4240 2.9426 2.9241 2.9129 2.9053 2.8944 2.8864 2.8756 2.8682 2.8570 2.1517 1.1757 1.1574
2.04 1.05 2.08 1.00 1.04 1.02 1.03 3.00 3.26
8.5 8.0 7.5 7.0 6.5 6.0 5.5 5.0 4.5 4.0 3.5 3.0 2.5 2.0 1.5 1.0 ppm
5m
University of HUDDERSFIELD
Current Data Parameters
NAME SO-307
EXPNO 20
PROCNO 1
F2 - Acquisition Parameters
Date_ 20150612
Time 14.30
INSTRUM spect
PROBHD 5 mm PABBO BB/
PULPROG zg30
TD 65536
SOLVENT CDCl3
NS 16
DS 2
SWH 8223.685 Hz
FIDRES 0.125483 Hz
AQ 3.9845889 sec
RG 28.42
DW 60.800 usec
DE 10.69 usec
TE 293.9 K
D1 2.00000000 sec
TD0 1
======== CHANNEL f1 ========
SFO1 400.1324710 MHz
NUC1 1H
P1 8.00 usec
PLW1 24.00000000 W
F2 - Processing parameters
SI 32768
SF 400.1300094 MHz
WDW EM
SSB 0
LB 0.30 Hz
GB 0
PC 1.50

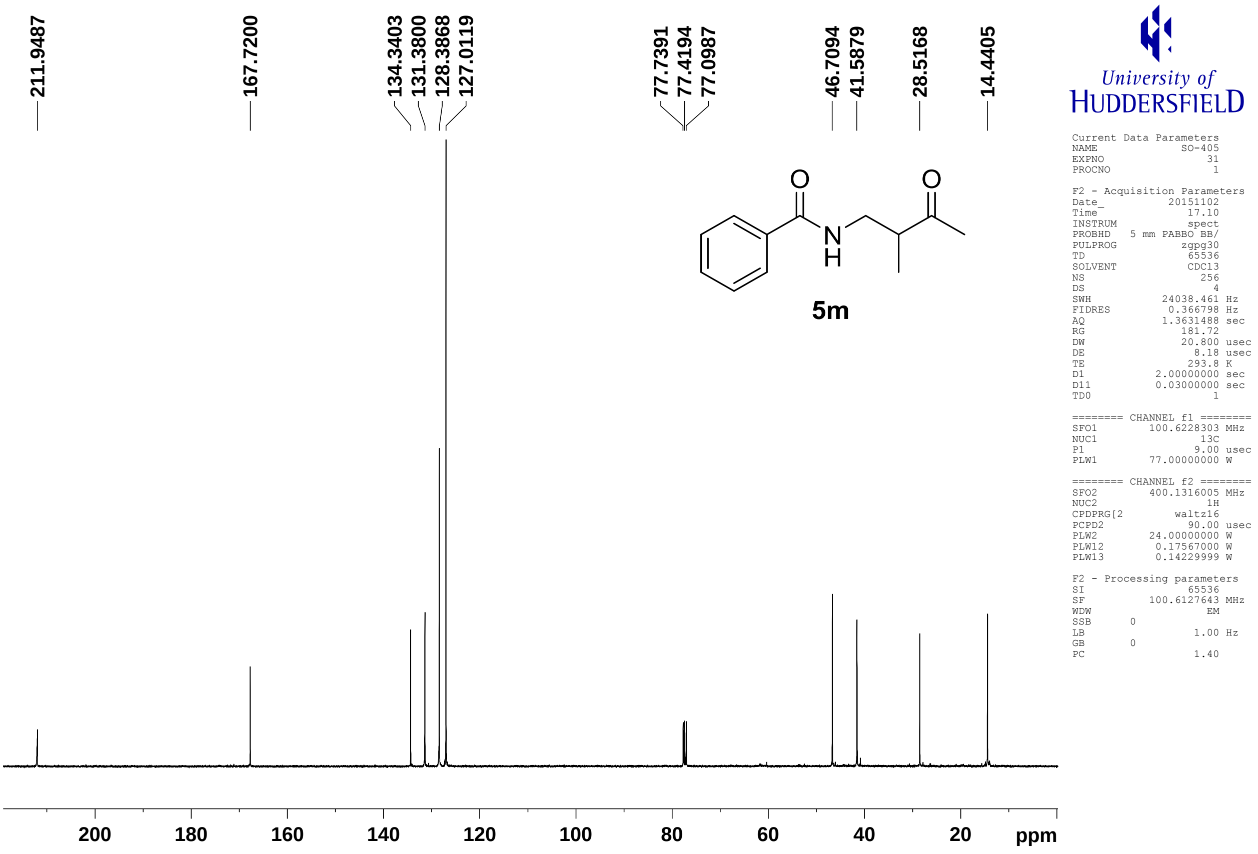
211.9487
167.7200
134.3403 131.3800 128.3868 127.0119
77.7391 77.4194 77.0987
46.7094 41.5879
28.5168
14.4405
200 180 160 140 120 100 80 60 40 20 ppm
5m
University of HUDDERSFIELD
Current Data Parameters
NAME SO-405
EXPNO 31
PROCNO 1
F2 - Acquisition Parameters
Date_ 20151102
Time 17.10
INSTRUM spect
PROBHD 5 mm PABBO BB/
PULPROG zgpg30
TD 65536
SOLVENT CDCl3
NS 256
DS 4
SWH 24038.461 Hz
FIDRES 0.366798 Hz
AQ 1.3631488 sec
RG 181.72
DW 20.800 usec
DE 8.18 usec
TE 293.8 K
D1 2.00000000 sec
D11 0.03000000 sec
TD0 1
======== CHANNEL f1 ========
SFO1 100.6228303 MHz
NUC1 13C
P1 9.00 usec
PLW1 77.00000000 W
======== CHANNEL f2 ========
SFO2 400.1316005 MHz
NUC2 1H
CPDPRG[2 waltz16
PCPD2 90.00 usec
PLW2 24.00000000 W
PLW12 0.17567000 W
PLW13 0.14229999 W
F2 - Processing parameters
SI 65536
SF 100.6127643 MHz
WDW EM
SSB 0
LB 1.00 Hz
GB 0
PC 1.40

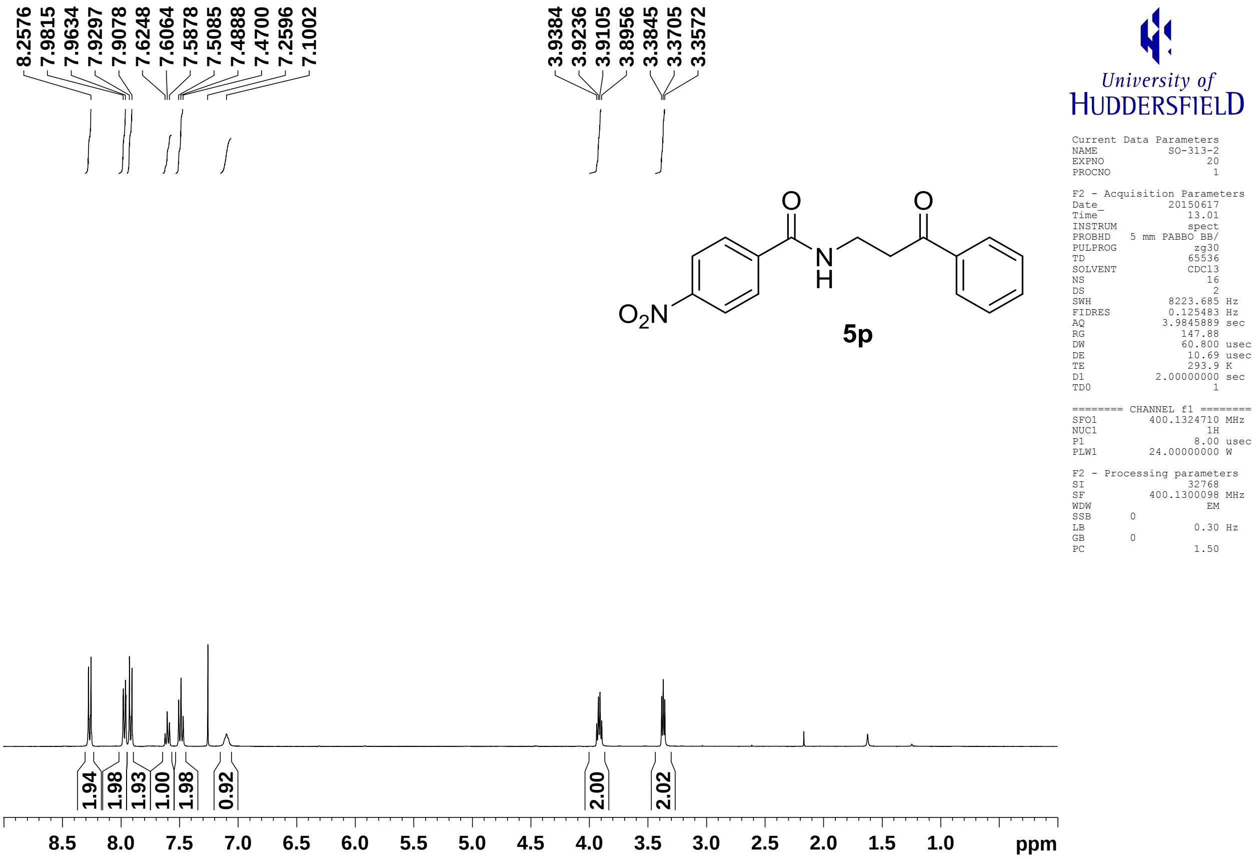
8.2576
7.9815
7.9634
7.9297
7.9078
7.6248
7.6064
7.5878
7.5085
7.4888
7.4700
7.2596
7.1002
3.9384
3.9236
3.9105
3.8956
3.3845
3.3705
3.3572
University of HUDDERSFIELD
Current Data Parameters
NAME SO-313-2
EXPNO 20
PROCNO 1
F2 - Acquisition Parameters
Date_ 20150617
Time 13.01
INSTRUM spect
PROBHD 5 mm PABBO BB/
PULPROG zg30
TD 65536
SOLVENT CDCl3
NS 16
DS 2
SWH 8223.685 Hz
FIDRES 0.125483 Hz
AQ 3.9845889 sec
RG 147.88
DW 60.800 usec
DE 10.69 usec
TE 293.9 K
D1 2.00000000 sec
TD0 1
======== CHANNEL f1 ========
SFO1 400.1324710 MHz
NUC1 1H
P1 8.00 usec
PLW1 24.00000000 W
F2 - Processing parameters
SI 32768
SF 400.1300098 MHz
WDW EM
SSB 0
LB 0.30 Hz
GB 0
PC 1.50
O
O
N
H
O_2N
5p
1.94
1.98
1.93
1.00
1.98
0.92
2.00
2.02
8.5
8.0
7.5
7.0
6.5
6.0
5.5
5.0
4.5
4.0
3.5
3.0
2.5
2.0
1.5
1.0
ppm

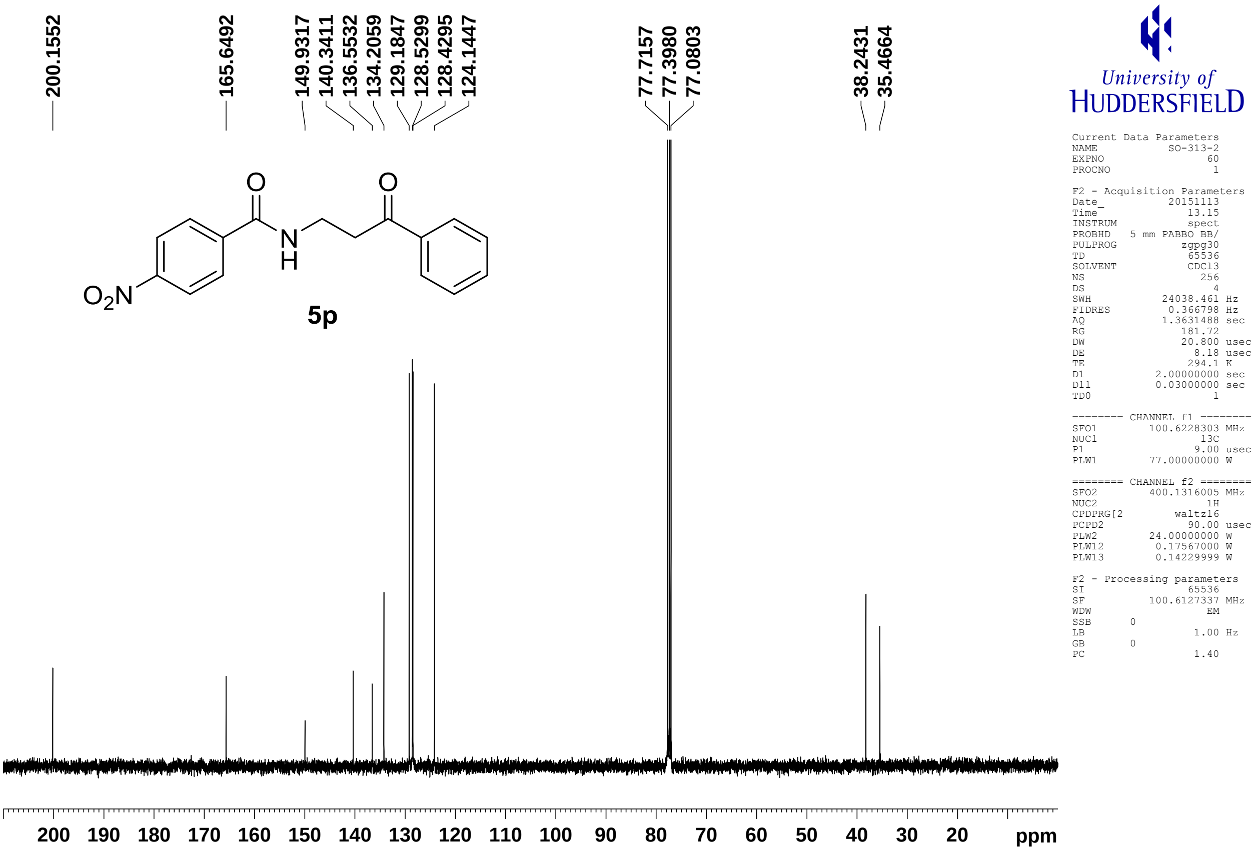
200.1552
165.6492
149.9317
140.3411
136.5532
134.2059
129.1847
128.5299
128.4295
124.1447
77.7157
77.3980
77.0803
38.2431
35.4664
University of HUDDERSFIELD
Current Data Parameters
NAME SO-313-2
EXPNO 60
PROCNO 1
F2 - Acquisition Parameters
Date_ 20151113
Time 13.15
INSTRUM spect
PROBHD 5 mm PABBO BB/
PULPROG zgpg30
TD 65536
SOLVENT CDCl3
NS 256
DS 4
SWH 24038.461 Hz
FIDRES 0.366798 Hz
AQ 1.3631488 sec
RG 181.72
DW 20.800 usec
DE 8.18 usec
TE 294.1 K
D1 2.00000000 sec
D11 0.03000000 sec
TD0 1
======== CHANNEL f1 ========
SFO1 100.6228303 MHz
NUC1 13C
P1 9.00 usec
PLW1 77.00000000 W
======== CHANNEL f2 ========
SFO2 400.1316005 MHz
NUC2 1H
CPDPRG[2 waltz16
PCPD2 90.00 usec
PLW2 24.00000000 W
PLW12 0.17567000 W
PLW13 0.14229999 W
F2 - Processing parameters
SI 65536
SF 100.6127337 MHz
WDW EM
SSB 0
LB 1.00 Hz
GB 0
PC 1.40
O
O
N
H
O_2N
5p
200
190
180
170
160
150
140
130
120
110
100
90
80
70
60
50
40
30
20
ppm

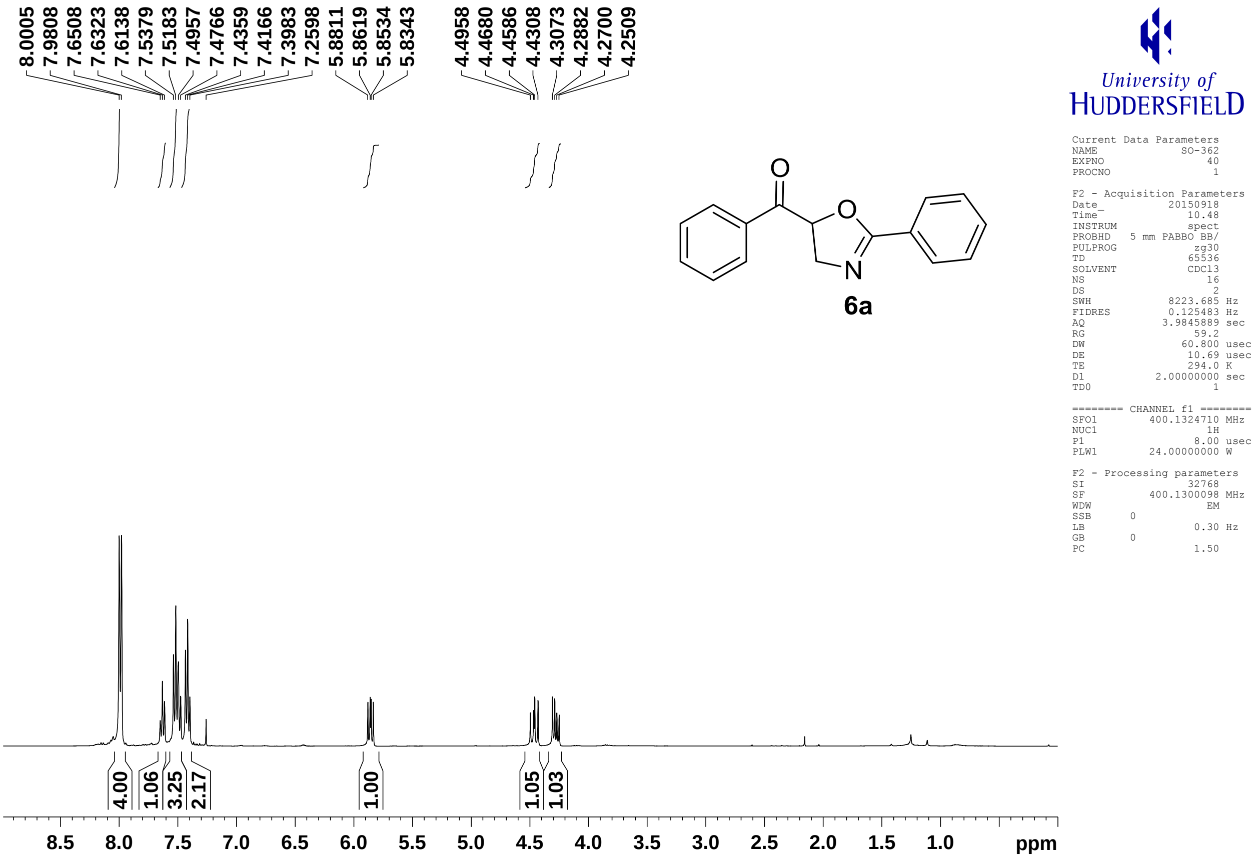

8.0005
7.9808
7.6508
7.6323
7.6138
7.5379
7.5183
7.4957
7.4766
7.4359
7.4166
7.3983
7.2598
5.8811
5.8619
5.8534
5.8343
4.4958
4.4680
4.4586
4.4308
4.3073
4.2882
4.2700
4.2509
O
O
N
6a
4.00
1.06
3.25
2.17
1.00
1.05
1.03
8.5 8.0 7.5 7.0 6.5 6.0 5.5 5.0 4.5 4.0 3.5 3.0 2.5 2.0 1.5 1.0 ppm
University of HUDDERSFIELD
Current Data Parameters
NAME SO-362
EXPNO 40
PROCNO 1
F2 - Acquisition Parameters
Date_ 20150918
Time 10.48
INSTRUM spect
PROBHD 5 mm PABBO BB/
PULPROG zg30
TD 65536
SOLVENT CDCl3
NS 16
DS 2
SWH 8223.685 Hz
FIDRES 0.125483 Hz
AQ 3.9845889 sec
RG 59.2
DW 60.800 usec
DE 10.69 usec
TE 294.0 K
D1 2.00000000 sec
TD0 1
======== CHANNEL f1 ========
SFO1 400.1324710 MHz
NUC1 1H
P1 8.00 usec
PLW1 24.00000000 W
F2 - Processing parameters
SI 32768
SF 400.1300098 MHz
WDW EM
SSB 0
LB 0.30 Hz
GB 0
PC 1.50

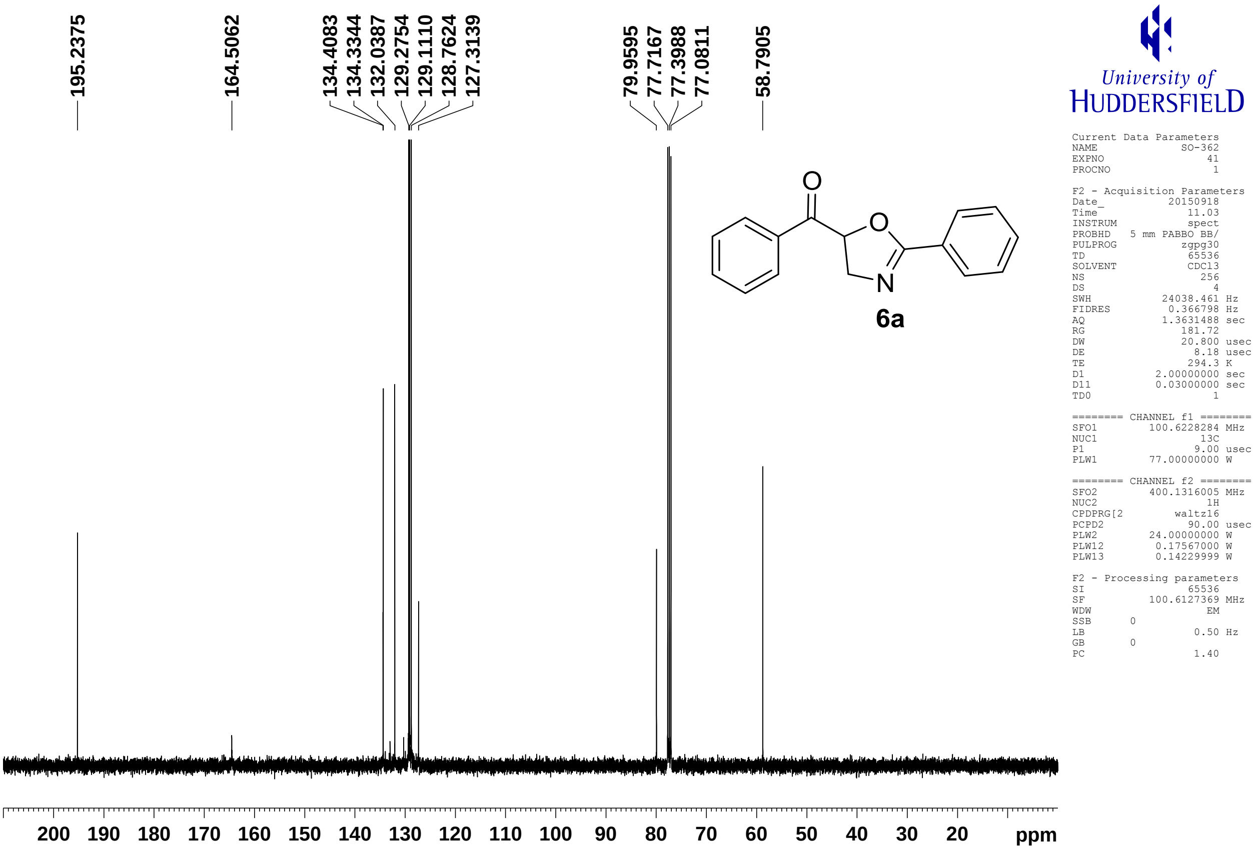

195.2375
164.5062
134.4083
134.3344
132.0387
129.2754
129.1110
128.7624
127.3139
79.9595
77.7167
77.3988
77.0811
58.7905
O
O
N
6a
200 190 180 170 160 150 140 130 120 110 100 90 80 70 60 50 40 30 20 ppm
University of HUDDERSFIELD
Current Data Parameters
NAME SO-362
EXPNO 41
PROCNO 1
F2 - Acquisition Parameters
Date_ 20150918
Time 11.03
INSTRUM spect
PROBHD 5 mm PABBO BB/
PULPROG zgpg30
TD 65536
SOLVENT CDCl3
NS 256
DS 4
SWH 24038.461 Hz
FIDRES 0.366798 Hz
AQ 1.3631488 sec
RG 181.72
DW 20.800 usec
DE 8.18 usec
TE 294.3 K
D1 2.00000000 sec
D11 0.03000000 sec
TD0 1
======== CHANNEL f1 ========
SFO1 100.6228284 MHz
NUC1 13C
P1 9.00 usec
PLW1 77.00000000 W
======== CHANNEL f2 ========
SFO2 400.1316005 MHz
NUC2 1H
CPDPRG[2 waltz16
PCPD2 90.00 usec
PLW2 24.00000000 W
PLW12 0.17567000 W
PLW13 0.14229999 W
F2 - Processing parameters
SI 65536
SF 100.6127369 MHz
WDW EM
SSB 0
LB 0.50 Hz
GB 0
PC 1.40

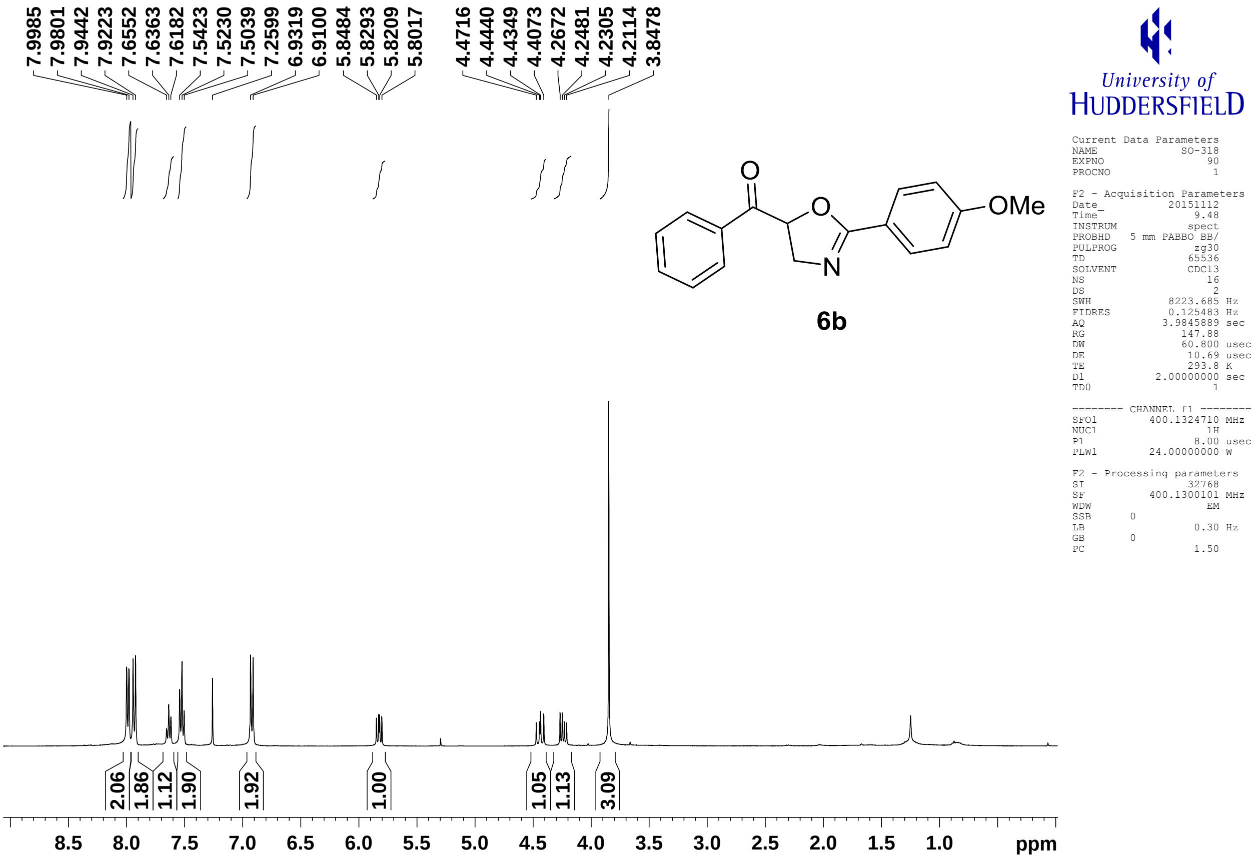
7.9985 7.9801 7.9442 7.9223 7.6552 7.6363 7.6182 7.5423 7.5230 7.5039 7.2599 6.9319 6.9100 5.8484 5.8293 5.8209 5.8017 4.4716 4.4440 4.4349 4.4073 4.2672 4.2481 4.2305 4.2114 3.8478
OMe
O
N
6b
2.06 1.86 1.12 1.90 1.92 1.00 1.05 1.13 3.09
8.5 8.0 7.5 7.0 6.5 6.0 5.5 5.0 4.5 4.0 3.5 3.0 2.5 2.0 1.5 1.0 ppm
University of HUDDERSFIELD
Current Data Parameters
NAME SO-318
EXPNO 90
PROCNO 1
F2 - Acquisition Parameters
Date_ 20151112
Time 9.48
INSTRUM spect
PROBHD 5 mm PABBO BB/
PULPROG zg30
TD 65536
SOLVENT CDCl3
NS 16
DS 2
SWH 8223.685 Hz
FIDRES 0.125483 Hz
AQ 3.9845889 sec
RG 147.88
DW 60.800 usec
DE 10.69 usec
TE 293.8 K
D1 2.00000000 sec
TD0 1
======== CHANNEL f1 ========
SFO1 400.1324710 MHz
NUC1 1H
P1 8.00 usec
PLW1 24.00000000 W
F2 - Processing parameters
SI 32768
SF 400.1300101 MHz
WDW EM
SSB 0
LB 0.30 Hz
GB 0
PC 1.50

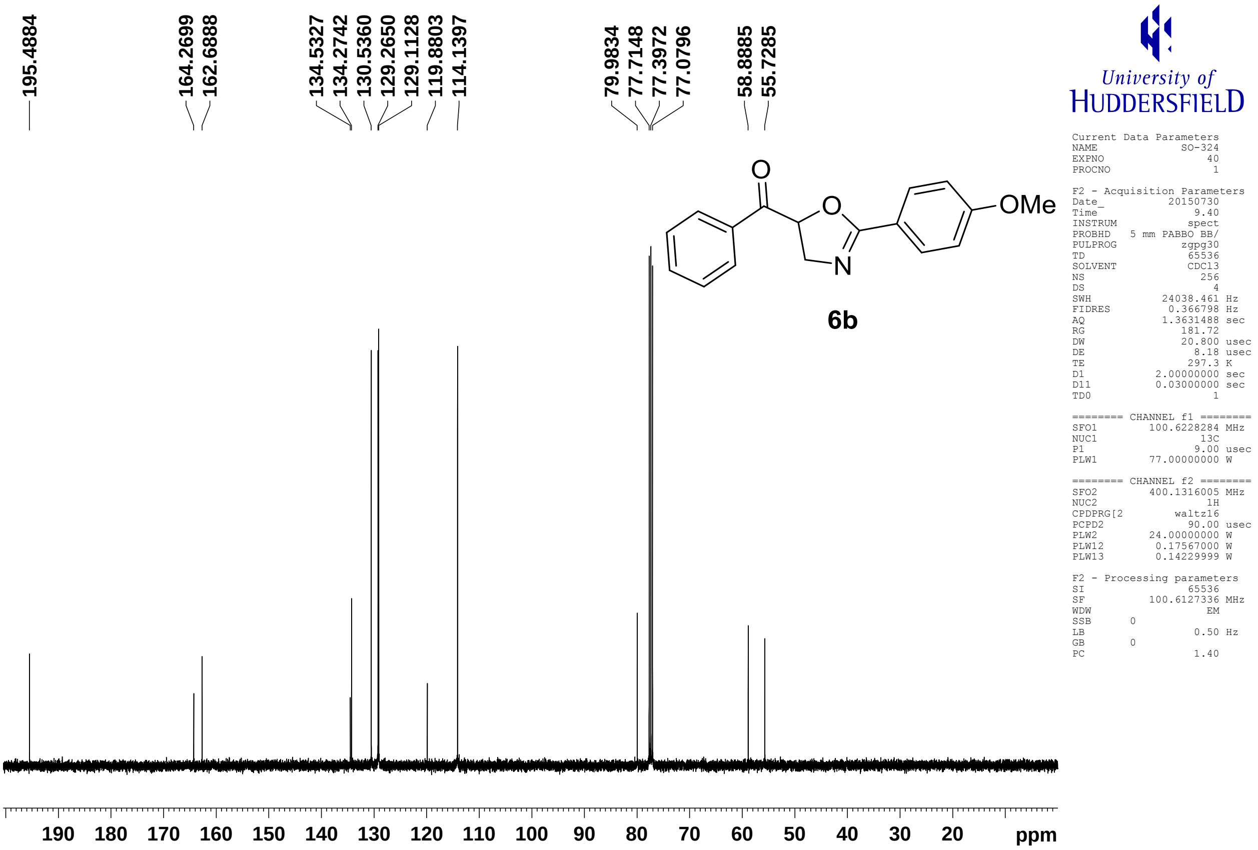
195.4884 164.2699 162.6888 134.5327 134.2742 130.5360 129.2650 129.1128 119.8803 114.1397 79.9834 77.7148 77.3972 77.0796 58.8885 55.7285
OMe
O
N
6b
190 180 170 160 150 140 130 120 110 100 90 80 70 60 50 40 30 20 ppm
University of HUDDERSFIELD
Current Data Parameters
NAME SO-324
EXPNO 40
PROCNO 1
F2 - Acquisition Parameters
Date_ 20150730
Time 9.40
INSTRUM spect
PROBHD 5 mm PABBO BB/
PULPROG zgpg30
TD 65536
SOLVENT CDCl3
NS 256
DS 4
SWH 24038.461 Hz
FIDRES 0.366798 Hz
AQ 1.3631488 sec
RG 181.72
DW 20.800 usec
DE 8.18 usec
TE 297.3 K
D1 2.00000000 sec
D11 0.03000000 sec
TD0 1
======== CHANNEL f1 ========
SFO1 100.6228284 MHz
NUC1 13C
P1 9.00 usec
PLW1 77.00000000 W
======== CHANNEL f2 ========
SFO2 400.1316005 MHz
NUC2 1H
CPDPRG[2 waltz16
PCPD2 90.00 usec
PLW2 24.00000000 W
PLW12 0.17567000 W
PLW13 0.14229999 W
F2 - Processing parameters
SI 65536
SF 100.6127336 MHz
WDW EM
SSB 0
LB 0.50 Hz
GB 0
PC 1.40

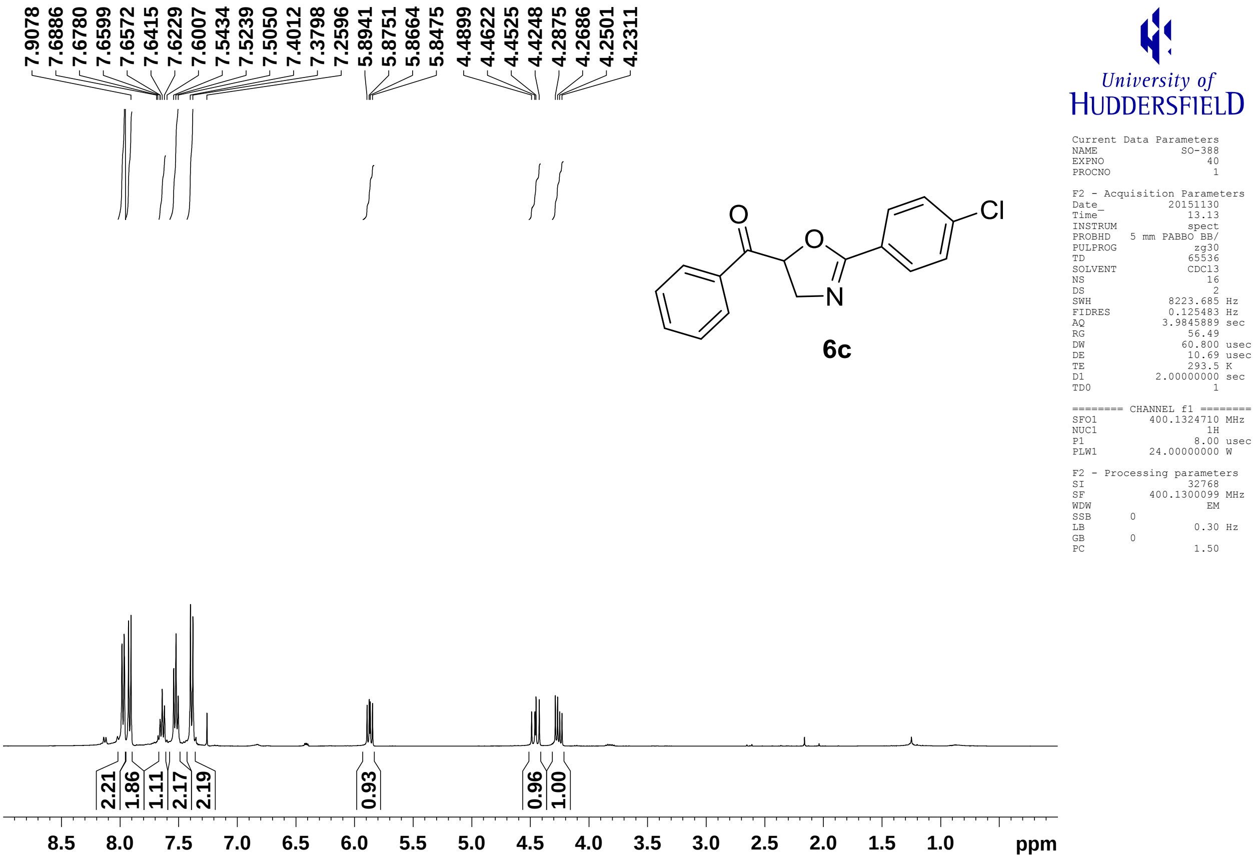
7.9078
7.6886
7.6780
7.6599
7.6572
7.6415
7.6229
7.6007
7.5434
7.5239
7.5050
7.4012
7.3798
7.2596
5.8941
5.8751
5.8664
5.8475
4.4899
4.4622
4.4525
4.4248
4.2875
4.2686
4.2501
4.2311
O
O
N
Cl
6c
University of HUDDERSFIELD
Current Data Parameters
NAME SO-388
EXPNO 40
PROCNO 1
F2 - Acquisition Parameters
Date_ 20151130
Time 13.13
INSTRUM spect
PROBHD 5 mm PABBO BB/
PULPROG zg30
TD 65536
SOLVENT CDCl3
NS 16
DS 2
SWH 8223.685 Hz
FIDRES 0.125483 Hz
AQ 3.9845889 sec
RG 56.49
DW 60.800 usec
DE 10.69 usec
TE 293.5 K
D1 2.00000000 sec
TD0 1
======== CHANNEL f1 ========
SFO1 400.1324710 MHz
NUC1 1H
P1 8.00 usec
PLW1 24.00000000 W
F2 - Processing parameters
SI 32768
SF 400.1300099 MHz
WDW EM
SSB 0
LB 0.30 Hz
GB 0
PC 1.50
2.21
1.86
1.11
2.17
2.19
0.93
0.96
1.00
8.5
8.0
7.5
7.0
6.5
6.0
5.5
5.0
4.5
4.0
3.5
3.0
2.5
2.0
1.5
1.0
ppm

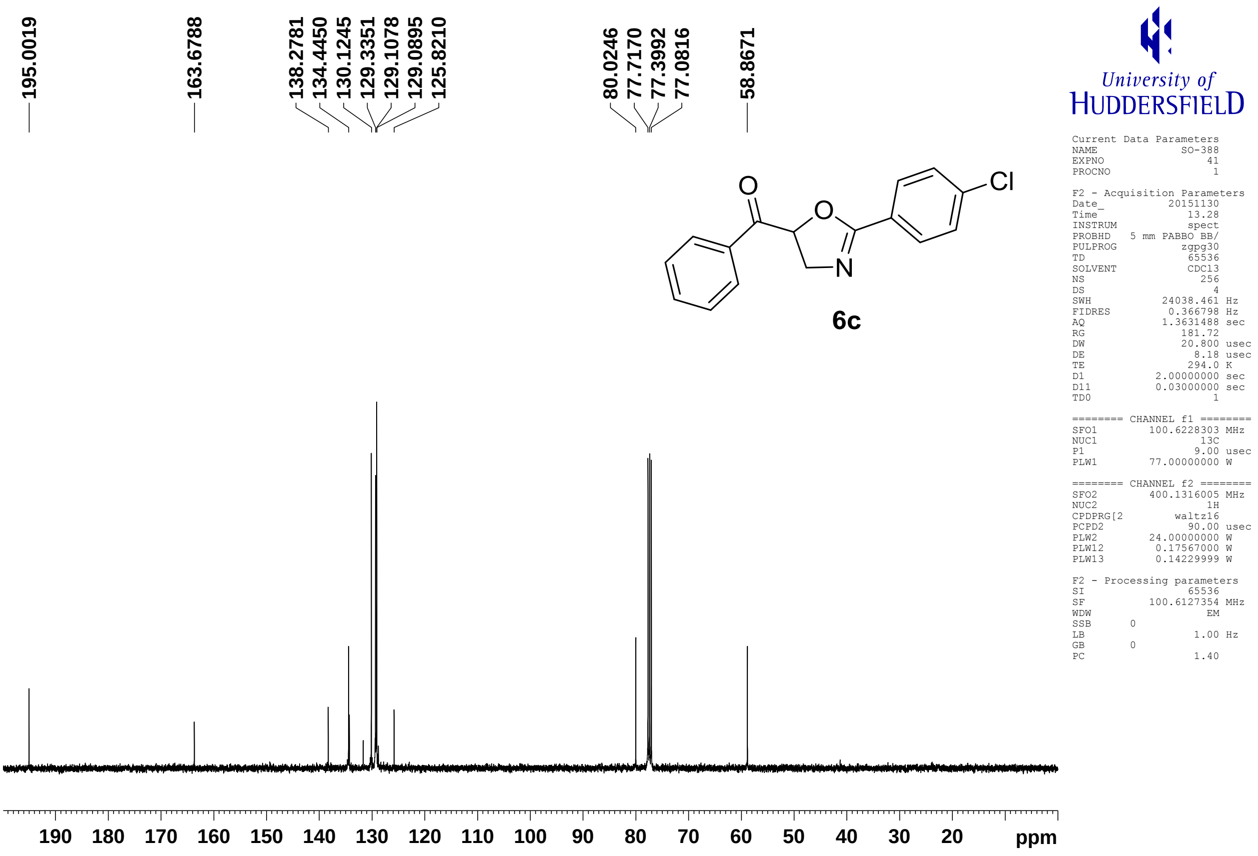
195.0019
163.6788
138.2781
134.4450
130.1245
129.3351
129.1078
129.0895
125.8210
80.0246
77.7170
77.3992
77.0816
58.8671
O
O
N
Cl
6c
University of HUDDERSFIELD
Current Data Parameters
NAME SO-388
EXPNO 41
PROCNO 1
F2 - Acquisition Parameters
Date_ 20151130
Time 13.28
INSTRUM spect
PROBHD 5 mm PABBO BB/
PULPROG zgpg30
TD 65536
SOLVENT CDCl3
NS 256
DS 4
SWH 24038.461 Hz
FIDRES 0.366798 Hz
AQ 1.3631488 sec
RG 181.72
DW 20.800 usec
DE 8.18 usec
TE 294.0 K
D1 2.00000000 sec
D11 0.03000000 sec
TD0 1
======== CHANNEL f1 ========
SFO1 100.6228303 MHz
NUC1 13C
P1 9.00 usec
PLW1 77.00000000 W
======== CHANNEL f2 ========
SFO2 400.1316005 MHz
NUC2 1H
CPDPRG[2 waltz16
PCPD2 90.00 usec
PLW2 24.00000000 W
PLW12 0.17567000 W
PLW13 0.14229999 W
F2 - Processing parameters
SI 65536
SF 100.6127354 MHz
WDW EM
SSB 0
LB 1.00 Hz
GB 0
PC 1.40
190
180
170
160
150
140
130
120
110
100
90
80
70
60
50
40
30
20
ppm

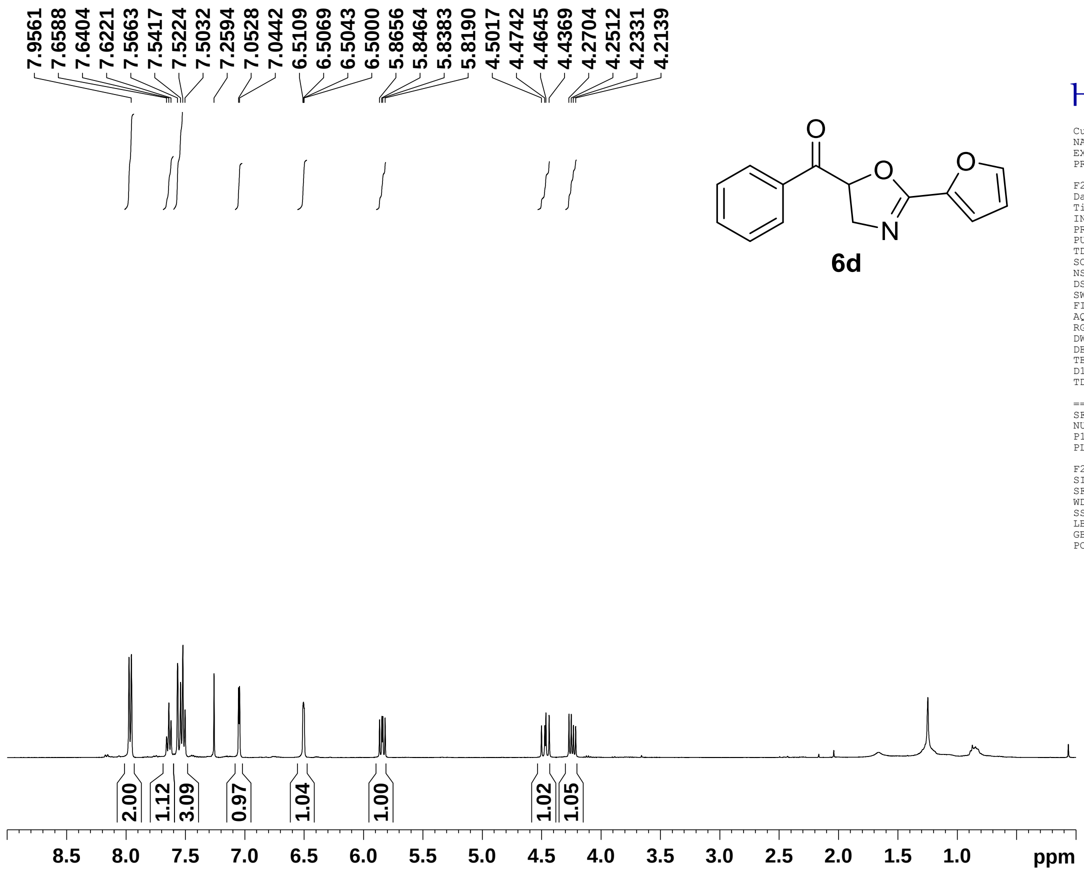

6d

University of HUDDERSFIELD

```
Current Data Parameters
NAME              SO-365
EXPNO                 40
PROCNO                 1

F2 - Acquisition Parameters
Date_           20150921
Time               10.58
INSTRUM            spect
PROBHD   5 mm PABBO BB/
PULPROG             zg30
TD                 65536
SOLVENT            CDCl3
NS                    16
DS                     2
SWH             8223.685 Hz
FIDRES          0.125483 Hz
AQ             3.9845889 sec
RG                147.88
DW                60.800 usec
DE                 10.69 usec
TE                 293.7 K
D1            2.00000000 sec
TD0                    1

======== CHANNEL f1 ========
SFO1         400.1324710 MHz
NUC1                  1H
P1                  8.00 usec
PLW1         24.00000000 W

F2 - Processing parameters
SI                 32768
SF           400.1300101 MHz
WDW                   EM
SSB      0
LB                  0.30 Hz
GB       0
PC                  1.50
```

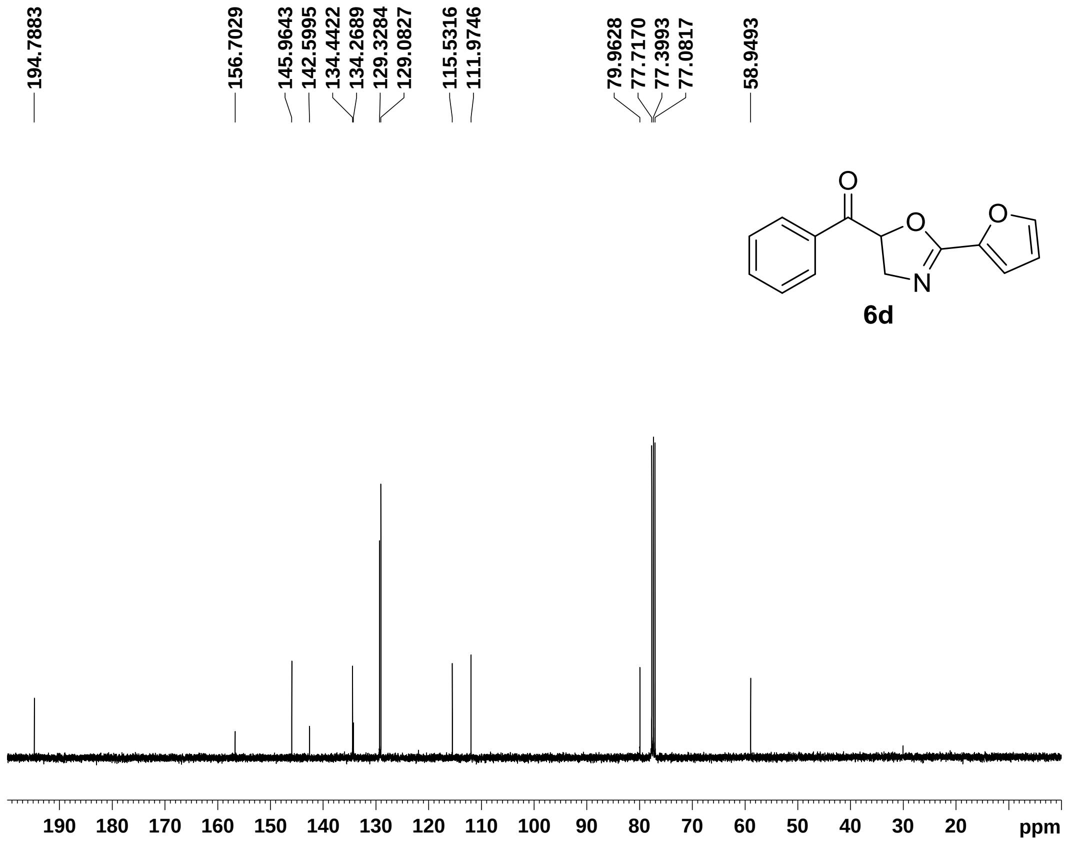

6d

University of HUDDERSFIELD

```
Current Data Parameters
NAME              SO-365
EXPNO                 71
PROCNO                 1

F2 - Acquisition Parameters
Date_           20150921
Time               13.36
INSTRUM            spect
PROBHD   5 mm PABBO BB/
PULPROG           zgpg30
TD                 65536
SOLVENT            CDCl3
NS                   256
DS                     4
SWH            24038.461 Hz
FIDRES          0.366798 Hz
AQ             1.3631488 sec
RG                181.72
DW                20.800 usec
DE                  8.18 usec
TE                 294.1 K
D1            2.00000000 sec
D11           0.03000000 sec
TD0                    1

======== CHANNEL f1 ========
SFO1         100.6228284 MHz
NUC1                 13C
P1                  9.00 usec
PLW1         77.00000000 W

======== CHANNEL f2 ========
SFO2         400.1316005 MHz
NUC2                  1H
CPDPRG[2         waltz16
PCPD2              90.00 usec
PLW2         24.00000000 W
PLW12         0.17567000 W
PLW13         0.14229999 W

F2 - Processing parameters
SI                 65536
SF           100.6127341 MHz
WDW                   EM
SSB      0
LB                  0.50 Hz
GB       0
PC                  1.40
```

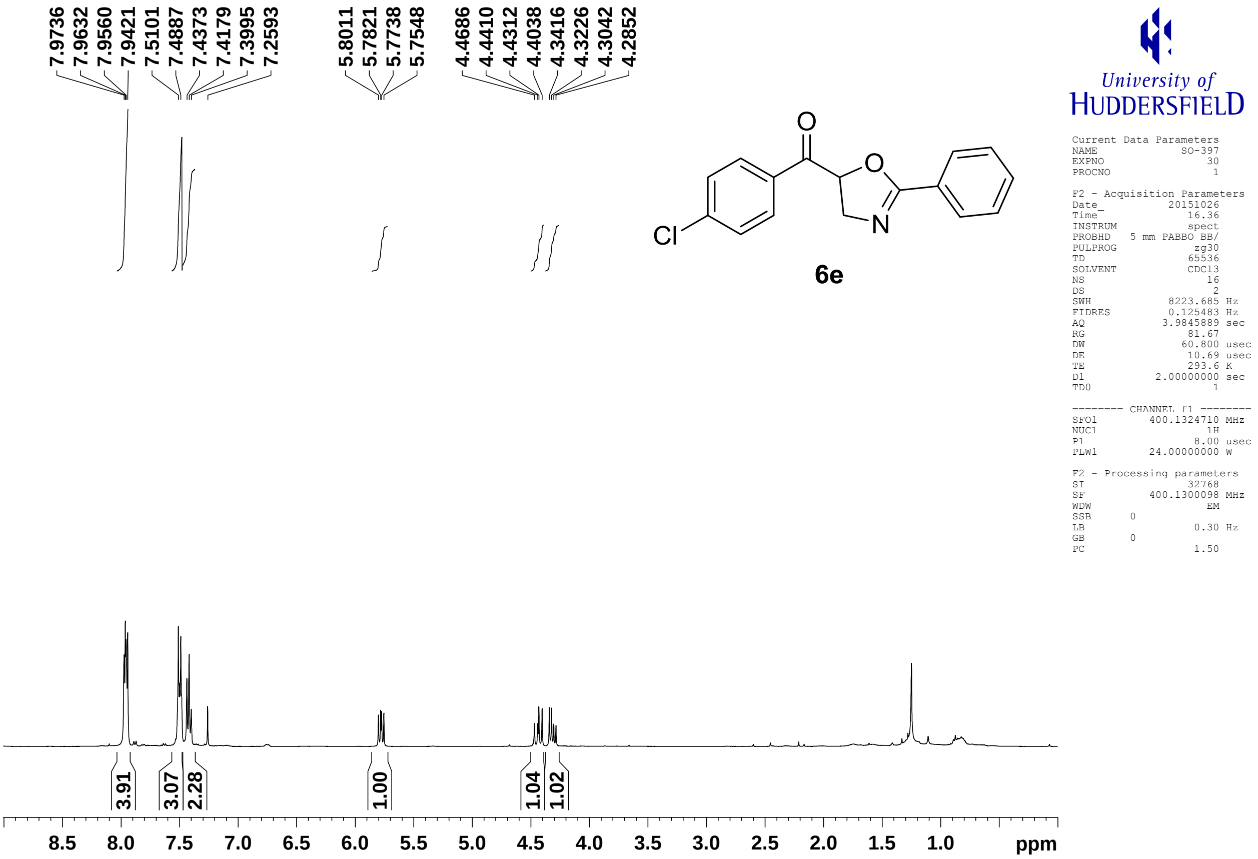
7.9736
7.9632
7.9560
7.9421
7.5101
7.4887
7.4373
7.4179
7.3995
7.2593
5.8011
5.7821
5.7738
5.7548
4.4686
4.4410
4.4312
4.4038
4.3416
4.3226
4.3042
4.2852
O
O
N
Cl
6e
3.91
3.07
2.28
1.00
1.04
1.02
8.5 8.0 7.5 7.0 6.5 6.0 5.5 5.0 4.5 4.0 3.5 3.0 2.5 2.0 1.5 1.0 ppm
University of HUDDERSFIELD
Current Data Parameters
NAME SO-397
EXPNO 30
PROCNO 1
F2 - Acquisition Parameters
Date_ 20151026
Time 16.36
INSTRUM spect
PROBHD 5 mm PABBO BB/
PULPROG zg30
TD 65536
SOLVENT CDCl3
NS 16
DS 2
SWH 8223.685 Hz
FIDRES 0.125483 Hz
AQ 3.9845889 sec
RG 81.67
DW 60.800 usec
DE 10.69 usec
TE 293.6 K
D1 2.00000000 sec
TD0 1
======== CHANNEL f1 ========
SFO1 400.1324710 MHz
NUC1 1H
P1 8.00 usec
PLW1 24.00000000 W
F2 - Processing parameters
SI 32768
SF 400.1300098 MHz
WDW EM
SSB 0
LB 0.30 Hz
GB 0
PC 1.50

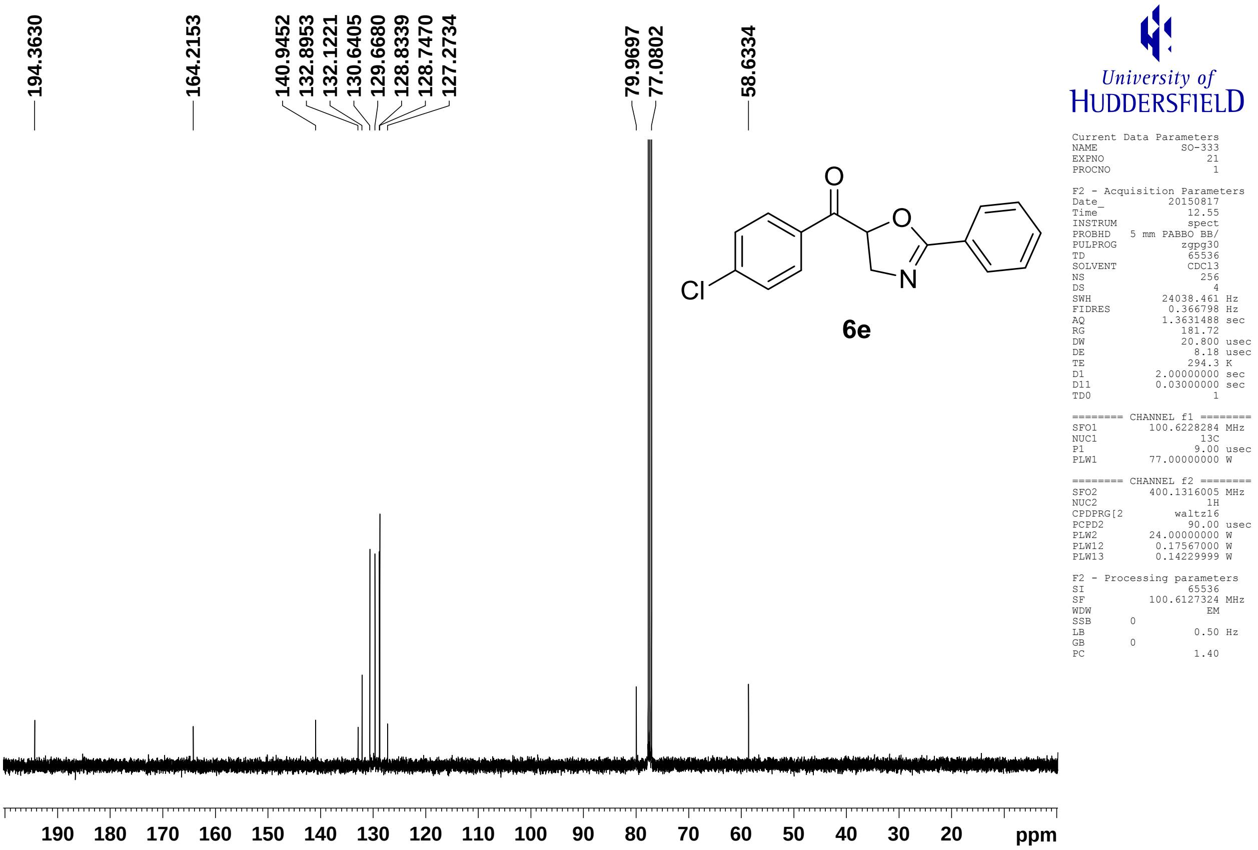
194.3630
164.2153
140.9452
132.8953
132.1221
130.6405
129.6680
128.8339
128.7470
127.2734
79.9697
77.0802
58.6334
O
O
N
Cl
6e
190 180 170 160 150 140 130 120 110 100 90 80 70 60 50 40 30 20 ppm
University of HUDDERSFIELD
Current Data Parameters
NAME SO-333
EXPNO 21
PROCNO 1
F2 - Acquisition Parameters
Date_ 20150817
Time 12.55
INSTRUM spect
PROBHD 5 mm PABBO BB/
PULPROG zgpg30
TD 65536
SOLVENT CDCl3
NS 256
DS 4
SWH 24038.461 Hz
FIDRES 0.366798 Hz
AQ 1.3631488 sec
RG 181.72
DW 20.800 usec
DE 8.18 usec
TE 294.3 K
D1 2.00000000 sec
D11 0.03000000 sec
TD0 1
======== CHANNEL f1 ========
SFO1 100.6228284 MHz
NUC1 13C
P1 9.00 usec
PLW1 77.00000000 W
======== CHANNEL f2 ========
SFO2 400.1316005 MHz
NUC2 1H
CPDPRG[2 waltz16
PCPD2 90.00 usec
PLW2 24.00000000 W
PLW12 0.17567000 W
PLW13 0.14229999 W
F2 - Processing parameters
SI 65536
SF 100.6127324 MHz
WDW EM
SSB 0
LB 0.50 Hz
GB 0
PC 1.40

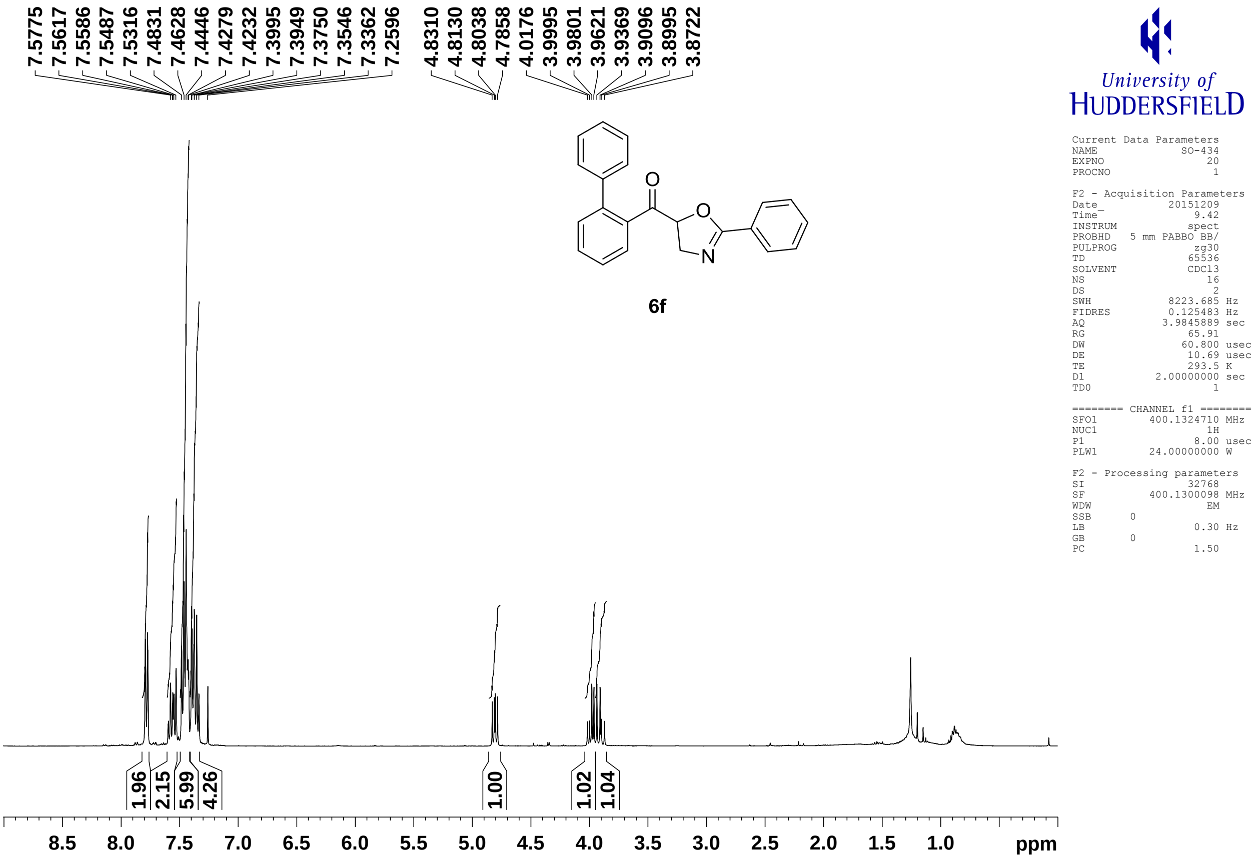
7.5775
7.5617
7.5586
7.5487
7.5316
7.4831
7.4628
7.4446
7.4279
7.4232
7.3995
7.3949
7.3750
7.3546
7.3362
7.2596
4.8310
4.8130
4.8038
4.7858
4.0176
3.9995
3.9801
3.9621
3.9369
3.9096
3.8995
3.8722
O
O
N
6f
1.96
2.15
5.99
4.26
1.00
1.02
1.04
8.5 8.0 7.5 7.0 6.5 6.0 5.5 5.0 4.5 4.0 3.5 3.0 2.5 2.0 1.5 1.0 ppm
University of HUDDERSFIELD
Current Data Parameters
NAME SO-434
EXPNO 20
PROCNO 1
F2 - Acquisition Parameters
Date_ 20151209
Time 9.42
INSTRUM spect
PROBHD 5 mm PABBO BB/
PULPROG zg30
TD 65536
SOLVENT CDCl3
NS 16
DS 2
SWH 8223.685 Hz
FIDRES 0.125483 Hz
AQ 3.9845889 sec
RG 65.91
DW 60.800 usec
DE 10.69 usec
TE 293.5 K
D1 2.00000000 sec
TD0 1
======== CHANNEL f1 ========
SFO1 400.1324710 MHz
NUC1 1H
P1 8.00 usec
PLW1 24.00000000 W
F2 - Processing parameters
SI 32768
SF 400.1300098 MHz
WDW EM
SSB 0
LB 0.30 Hz
GB 0
PC 1.50

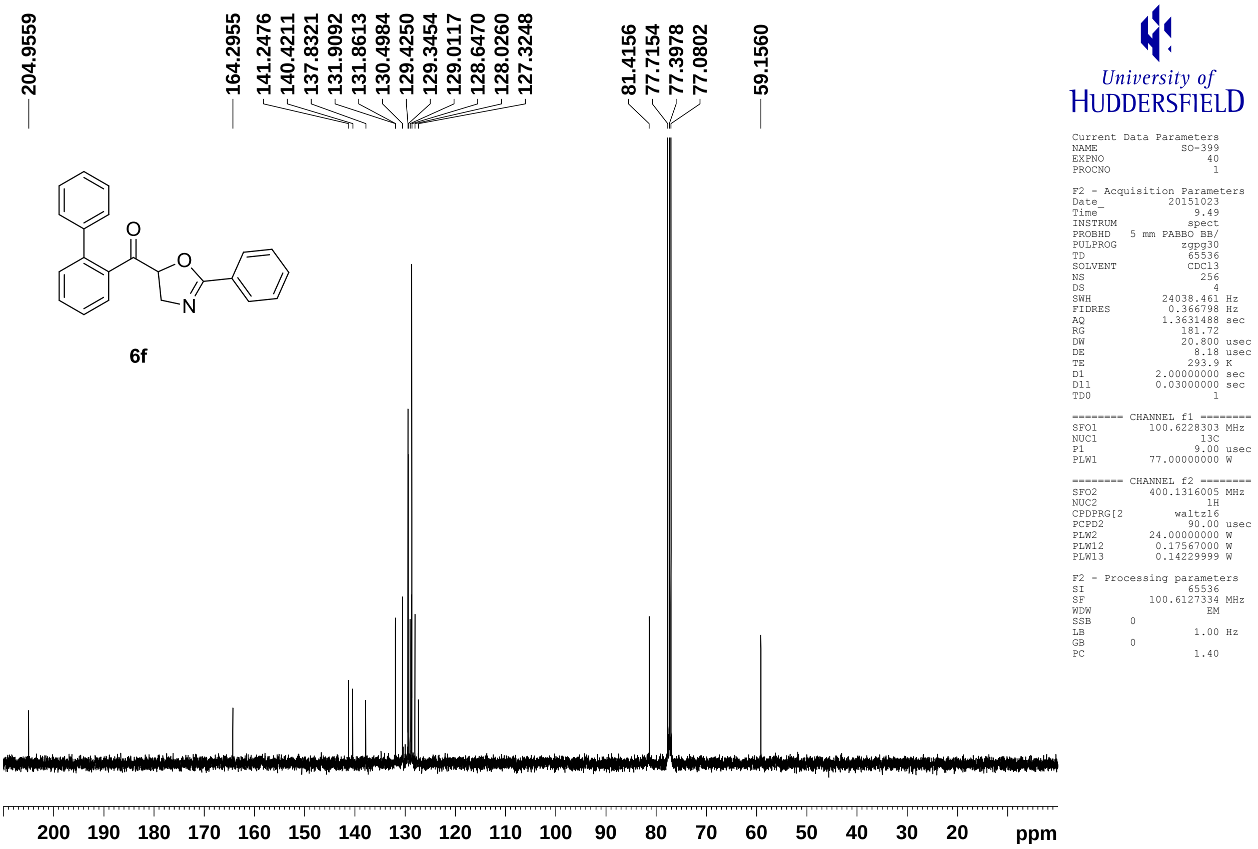
204.9559
164.2955
141.2476
140.4211
137.8321
131.9092
131.8613
130.4984
129.4250
129.3454
129.0117
128.6470
128.0260
127.3248
81.4156
77.7154
77.3978
77.0802
59.1560
O
O
N
6f
200 190 180 170 160 150 140 130 120 110 100 90 80 70 60 50 40 30 20 ppm
University of HUDDERSFIELD
Current Data Parameters
NAME SO-399
EXPNO 40
PROCNO 1
F2 - Acquisition Parameters
Date_ 20151023
Time 9.49
INSTRUM spect
PROBHD 5 mm PABBO BB/
PULPROG zgpg30
TD 65536
SOLVENT CDCl3
NS 256
DS 4
SWH 24038.461 Hz
FIDRES 0.366798 Hz
AQ 1.3631488 sec
RG 181.72
DW 20.800 usec
DE 8.18 usec
TE 293.9 K
D1 2.00000000 sec
D11 0.03000000 sec
TD0 1
======== CHANNEL f1 ========
SFO1 100.6228303 MHz
NUC1 13C
P1 9.00 usec
PLW1 77.00000000 W
======== CHANNEL f2 ========
SFO2 400.1316005 MHz
NUC2 1H
CPDPRG[2 waltz16
PCPD2 90.00 usec
PLW2 24.00000000 W
PLW12 0.17567000 W
PLW13 0.14229999 W
F2 - Processing parameters
SI 65536
SF 100.6127334 MHz
WDW EM
SSB 0
LB 1.00 Hz
GB 0
PC 1.40

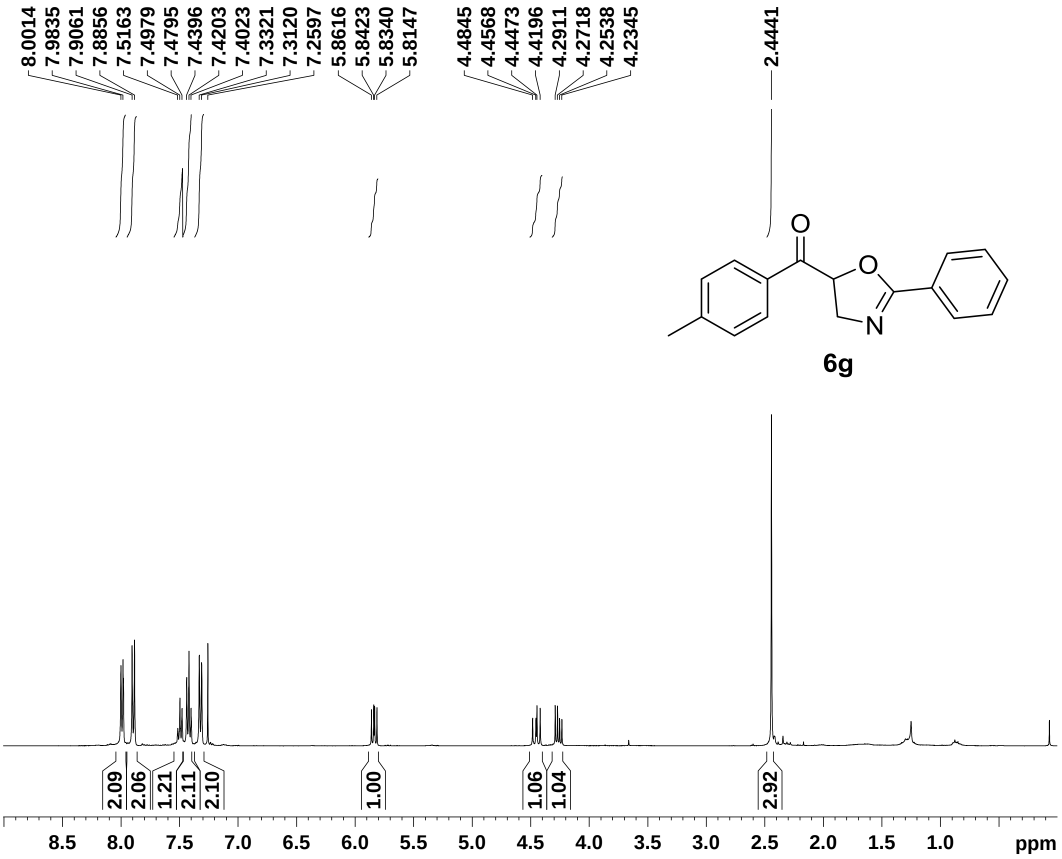

University of HUDDERSFIELD

```
Current Data Parameters
NAME            SO-441
EXPNO               30
PROCNO               1

F2 - Acquisition Parameters
Date_         20160113
Time             11.06
INSTRUM          spect
PROBHD   5 mm PABBO BB/
PULPROG           zg30
TD               65536
SOLVENT          CDCl3
NS                  16
DS                   2
SWH           8223.685 Hz
FIDRES        0.125483 Hz
AQ           3.9845889 sec
RG              147.88
DW              60.800 usec
DE               10.69 usec
TE               293.7 K
D1          2.00000000 sec
TD0                  1

======== CHANNEL f1 ========
SFO1       400.1324710 MHz
NUC1                1H
P1                8.00 usec
PLW1       24.00000000 W

F2 - Processing parameters
SI               32768
SF         400.1300100 MHz
WDW                 EM
SSB      0
LB                0.30 Hz
GB       0
PC                1.50
```

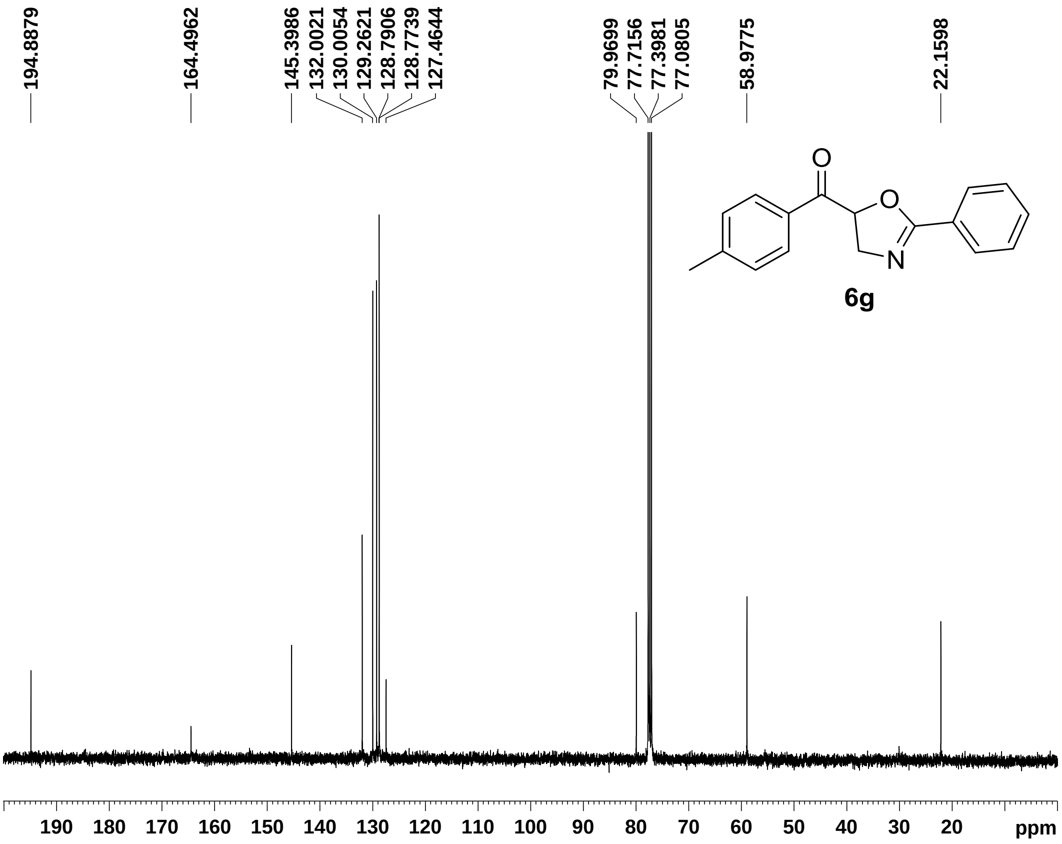

University of HUDDERSFIELD

```
Current Data Parameters
NAME            SO-441
EXPNO               40
PROCNO               1

F2 - Acquisition Parameters
Date_         20160114
Time              1.38
INSTRUM          spect
PROBHD   5 mm PABBO BB/
PULPROG         zgpg30
TD               65536
SOLVENT          CDCl3
NS                1024
DS                   4
SWH          24038.461 Hz
FIDRES        0.366798 Hz
AQ           1.3631488 sec
RG              181.72
DW              20.800 usec
DE                8.18 usec
TE               294.6 K
D1          2.00000000 sec
D11         0.03000000 sec
TD0                  1

======== CHANNEL f1 ========
SFO1       100.6228303 MHz
NUC1               13C
P1                9.00 usec
PLW1       77.00000000 W

======== CHANNEL f2 ========
SFO2       400.1316005 MHz
NUC2                1H
CPDPRG[2       waltz16
PCPD2            90.00 usec
PLW2       24.00000000 W
PLW12       0.17567000 W
PLW13       0.14229999 W

F2 - Processing parameters
SI               65536
SF         100.6127324 MHz
WDW                 EM
SSB      0
LB                1.00 Hz
GB       0
PC                1.40
```

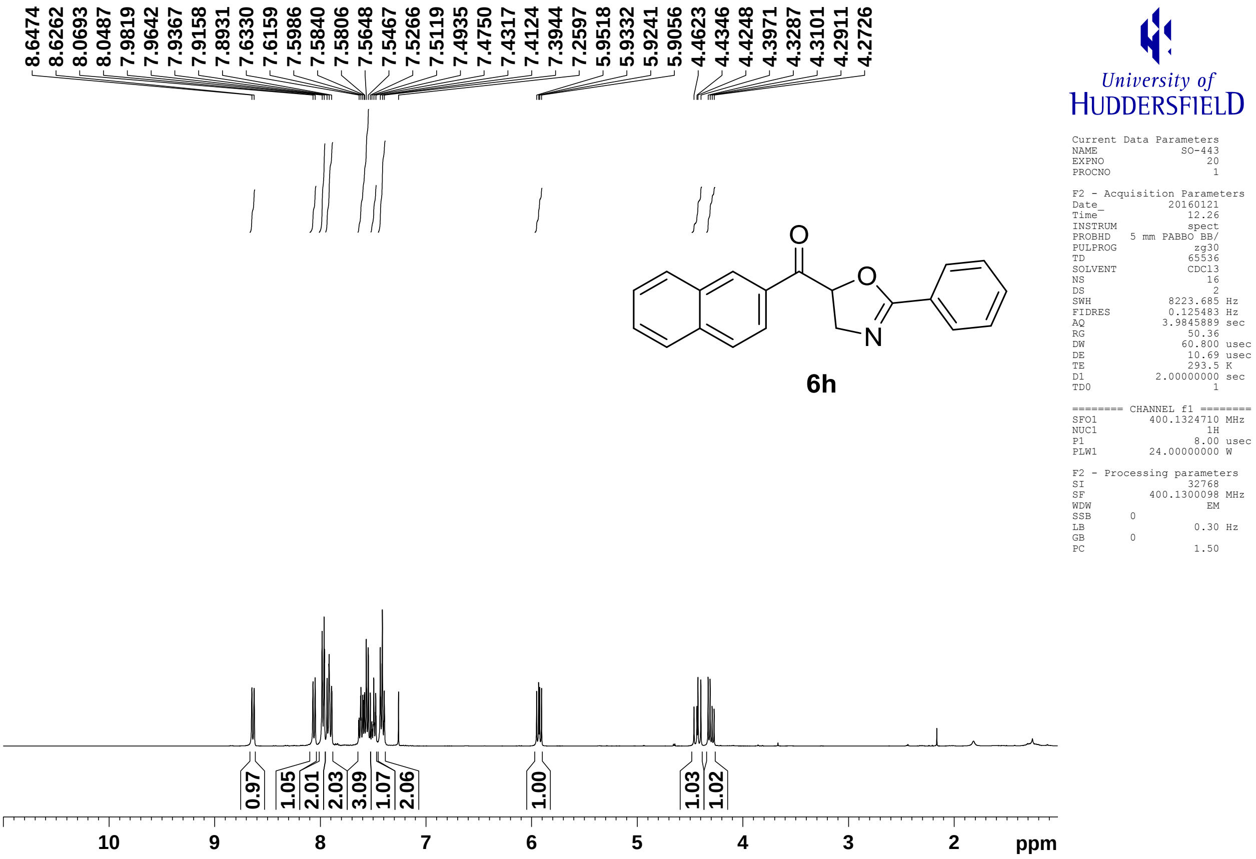
8.6474 8.6262 8.0693 8.0487 7.9819 7.9642 7.9367 7.9158 7.8931 7.6330 7.6159 7.5986 7.5840 7.5806 7.5648 7.5467 7.5266 7.5119 7.4935 7.4750 7.4317 7.4124 7.3944 7.2597 5.9518 5.9332 5.9241 5.9056 4.4623 4.4346 4.4248 4.3971 4.3287 4.3101 4.2911 4.2726
University of HUDDERSFIELD
Current Data Parameters
NAME SO-443
EXPNO 20
PROCNO 1
F2 - Acquisition Parameters
Date_ 20160121
Time 12.26
INSTRUM spect
PROBHD 5 mm PABBO BB/
PULPROG zg30
TD 65536
SOLVENT CDCl3
NS 16
DS 2
SWH 8223.685 Hz
FIDRES 0.125483 Hz
AQ 3.9845889 sec
RG 50.36
DW 60.800 usec
DE 10.69 usec
TE 293.5 K
D1 2.00000000 sec
TD0 1
======== CHANNEL f1 ========
SFO1 400.1324710 MHz
NUC1 1H
P1 8.00 usec
PLW1 24.00000000 W
F2 - Processing parameters
SI 32768
SF 400.1300098 MHz
WDW EM
SSB 0
LB 0.30 Hz
GB 0
PC 1.50
O
N
6h
0.97 1.05 2.01 2.03 3.09 1.07 2.06 1.00 1.03 1.02
10 9 8 7 6 5 4 3 2 ppm

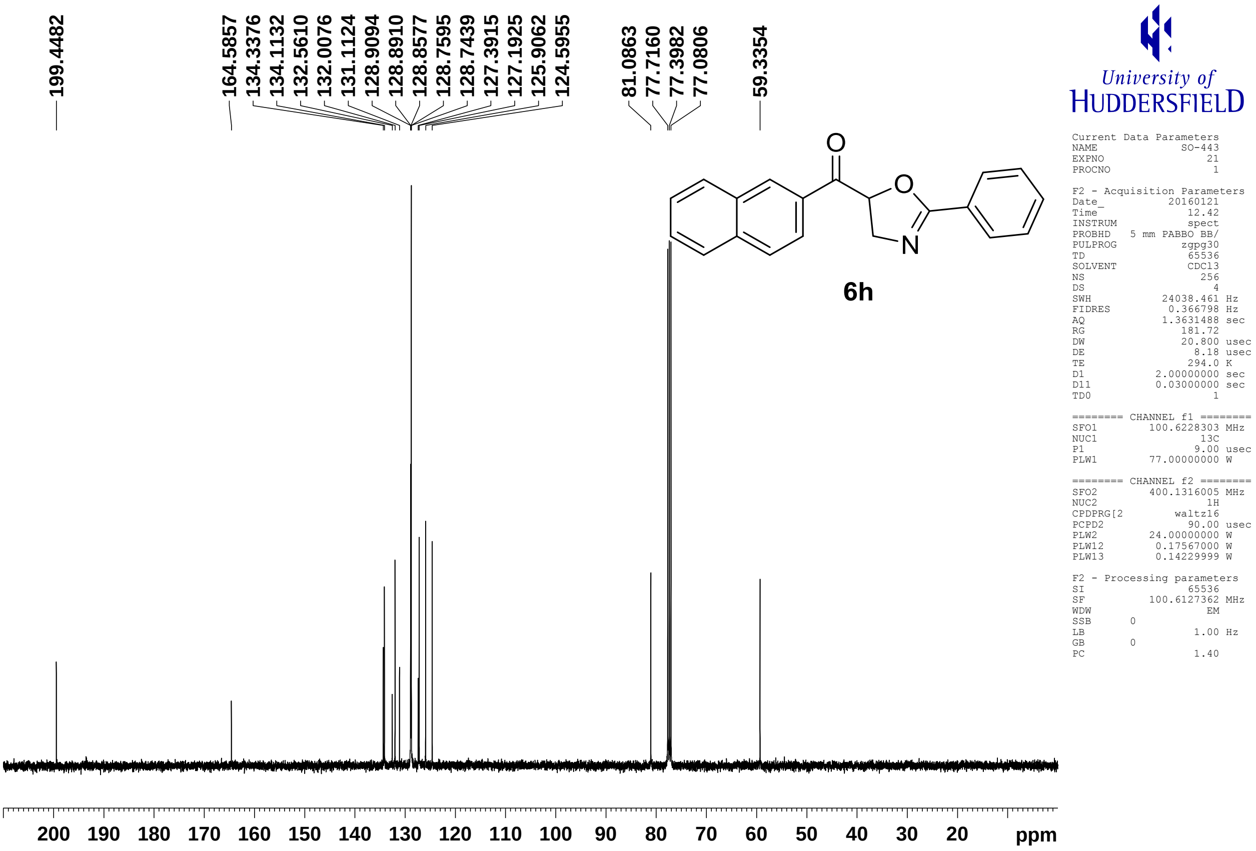
199.4482 164.5857 134.3376 134.1132 132.5610 132.0076 131.1124 128.9094 128.8910 128.8577 128.7595 128.7439 127.3915 127.1925 125.9062 124.5955 81.0863 77.7160 77.3982 77.0806 59.3354
University of HUDDERSFIELD
Current Data Parameters
NAME SO-443
EXPNO 21
PROCNO 1
F2 - Acquisition Parameters
Date_ 20160121
Time 12.42
INSTRUM spect
PROBHD 5 mm PABBO BB/
PULPROG zgpg30
TD 65536
SOLVENT CDCl3
NS 256
DS 4
SWH 24038.461 Hz
FIDRES 0.366798 Hz
AQ 1.3631488 sec
RG 181.72
DW 20.800 usec
DE 8.18 usec
TE 294.0 K
D1 2.00000000 sec
D11 0.03000000 sec
TD0 1
======== CHANNEL f1 ========
SFO1 100.6228303 MHz
NUC1 13C
P1 9.00 usec
PLW1 77.00000000 W
======== CHANNEL f2 ========
SFO2 400.1316005 MHz
NUC2 1H
CPDPRG[2 waltz16
PCPD2 90.00 usec
PLW2 24.00000000 W
PLW12 0.17567000 W
PLW13 0.14229999 W
F2 - Processing parameters
SI 65536
SF 100.6127362 MHz
WDW EM
SSB 0
LB 1.00 Hz
GB 0
PC 1.40
O
N
6h
200 190 180 170 160 150 140 130 120 110 100 90 80 70 60 50 40 30 20 ppm

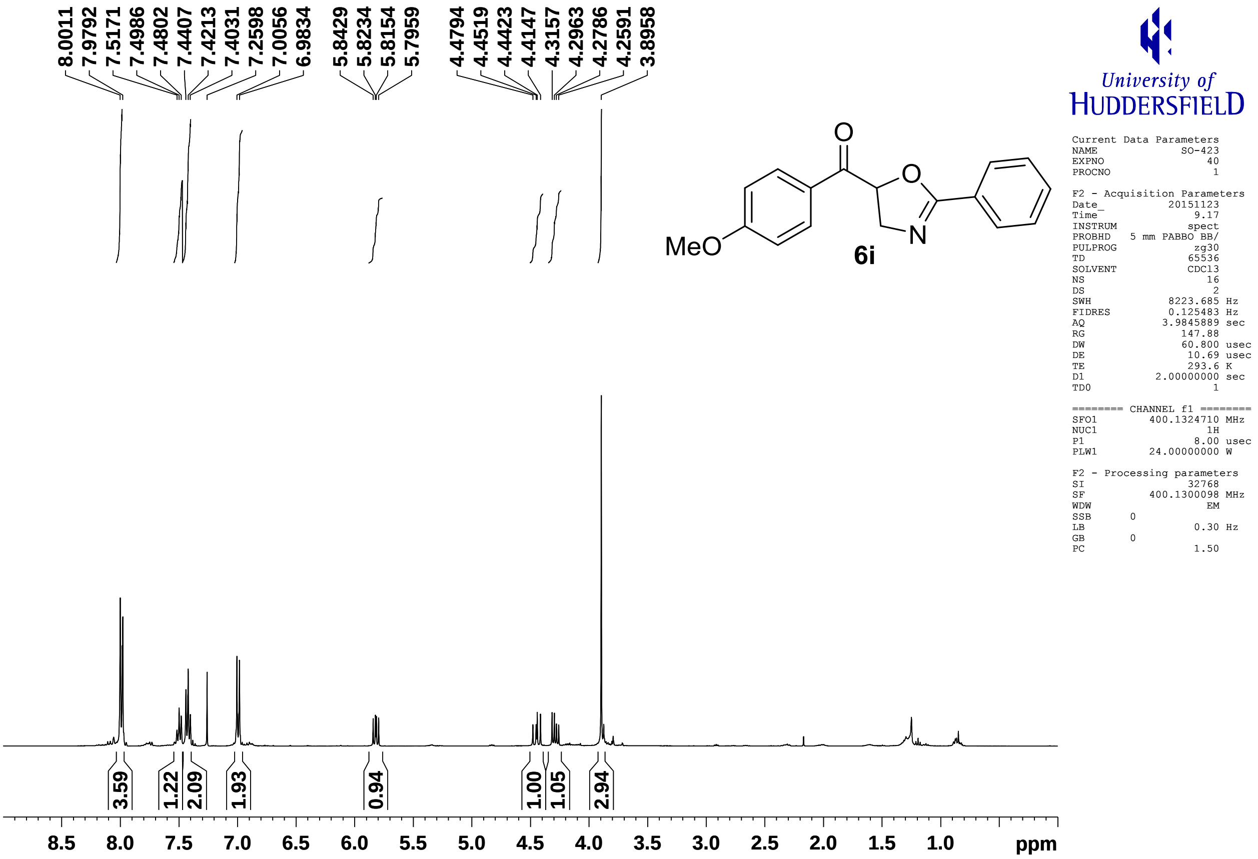
8.0011 7.9792 7.5171 7.4986 7.4802 7.4407 7.4213 7.4031 7.2598 7.0056 6.9834 5.8429 5.8234 5.8154 5.7959 4.4794 4.4519 4.4423 4.4147 4.3157 4.2963 4.2786 4.2591 3.8958
3.59 1.22 2.09 1.93 0.94 1.00 1.05 2.94
8.5 8.0 7.5 7.0 6.5 6.0 5.5 5.0 4.5 4.0 3.5 3.0 2.5 2.0 1.5 1.0 ppm
MeO
6i
University of HUDDERSFIELD
Current Data Parameters
NAME SO-423
EXPNO 40
PROCNO 1
F2 - Acquisition Parameters
Date_ 20151123
Time 9.17
INSTRUM spect
PROBHD 5 mm PABBO BB/
PULPROG zg30
TD 65536
SOLVENT CDCl3
NS 16
DS 2
SWH 8223.685 Hz
FIDRES 0.125483 Hz
AQ 3.9845889 sec
RG 147.88
DW 60.800 usec
DE 10.69 usec
TE 293.6 K
D1 2.00000000 sec
TD0 1
======== CHANNEL f1 ========
SFO1 400.1324710 MHz
NUC1 1H
P1 8.00 usec
PLW1 24.00000000 W
F2 - Processing parameters
SI 32768
SF 400.1300098 MHz
WDW EM
SSB 0
LB 0.30 Hz
GB 0
PC 1.50

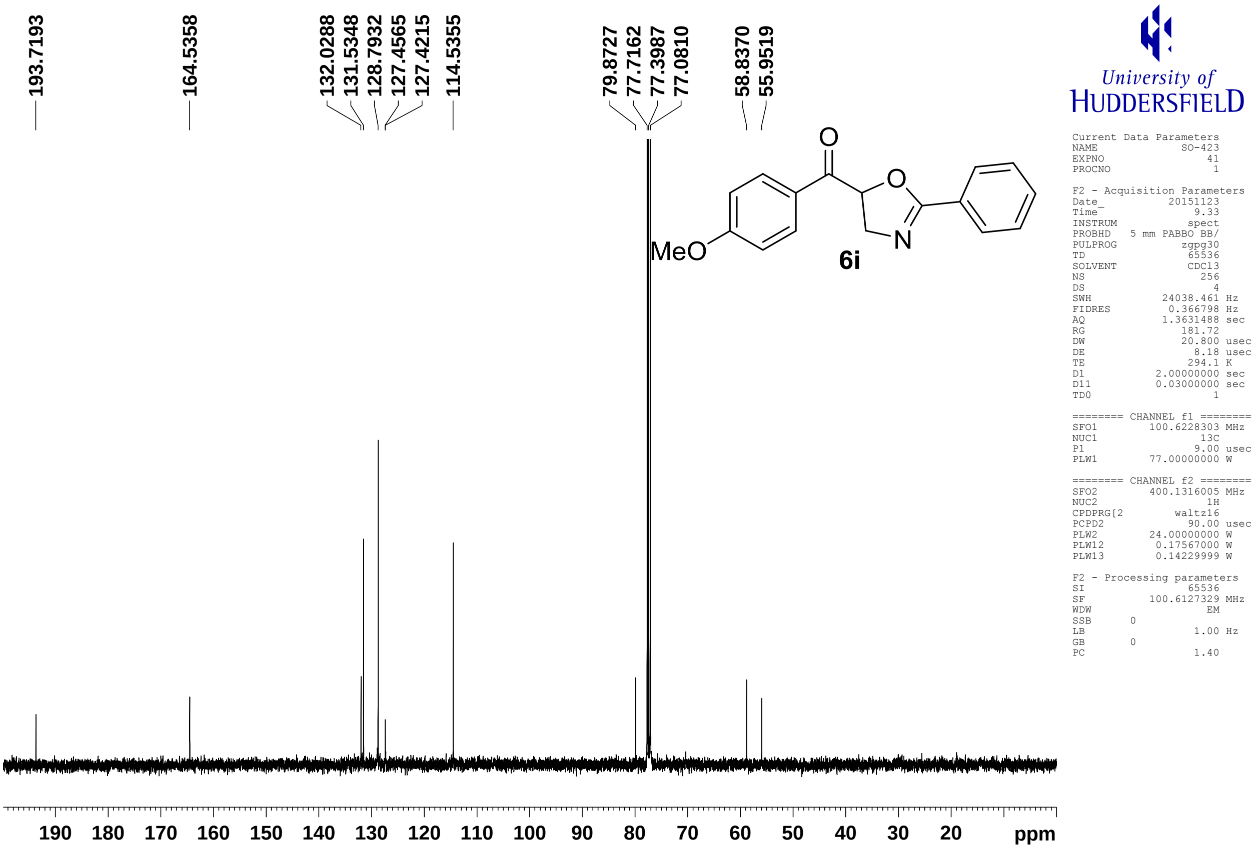
193.7193 164.5358 132.0288 131.5348 128.7932 127.4565 127.4215 114.5355 79.8727 77.7162 77.3987 77.0810 58.8370 55.9519
190 180 170 160 150 140 130 120 110 100 90 80 70 60 50 40 30 20 ppm
MeO
6i
University of HUDDERSFIELD
Current Data Parameters
NAME SO-423
EXPNO 41
PROCNO 1
F2 - Acquisition Parameters
Date_ 20151123
Time 9.33
INSTRUM spect
PROBHD 5 mm PABBO BB/
PULPROG zgpg30
TD 65536
SOLVENT CDCl3
NS 256
DS 4
SWH 24038.461 Hz
FIDRES 0.366798 Hz
AQ 1.3631488 sec
RG 181.72
DW 20.800 usec
DE 8.18 usec
TE 294.1 K
D1 2.00000000 sec
D11 0.03000000 sec
TD0 1
======== CHANNEL f1 ========
SFO1 100.6228303 MHz
NUC1 13C
P1 9.00 usec
PLW1 77.00000000 W
======== CHANNEL f2 ========
SFO2 400.1316005 MHz
NUC2 1H
CPDPRG[2 waltz16
PCPD2 90.00 usec
PLW2 24.00000000 W
PLW12 0.17567000 W
PLW13 0.14229999 W
F2 - Processing parameters
SI 65536
SF 100.6127329 MHz
WDW EM
SSB 0
LB 1.00 Hz
GB 0
PC 1.40

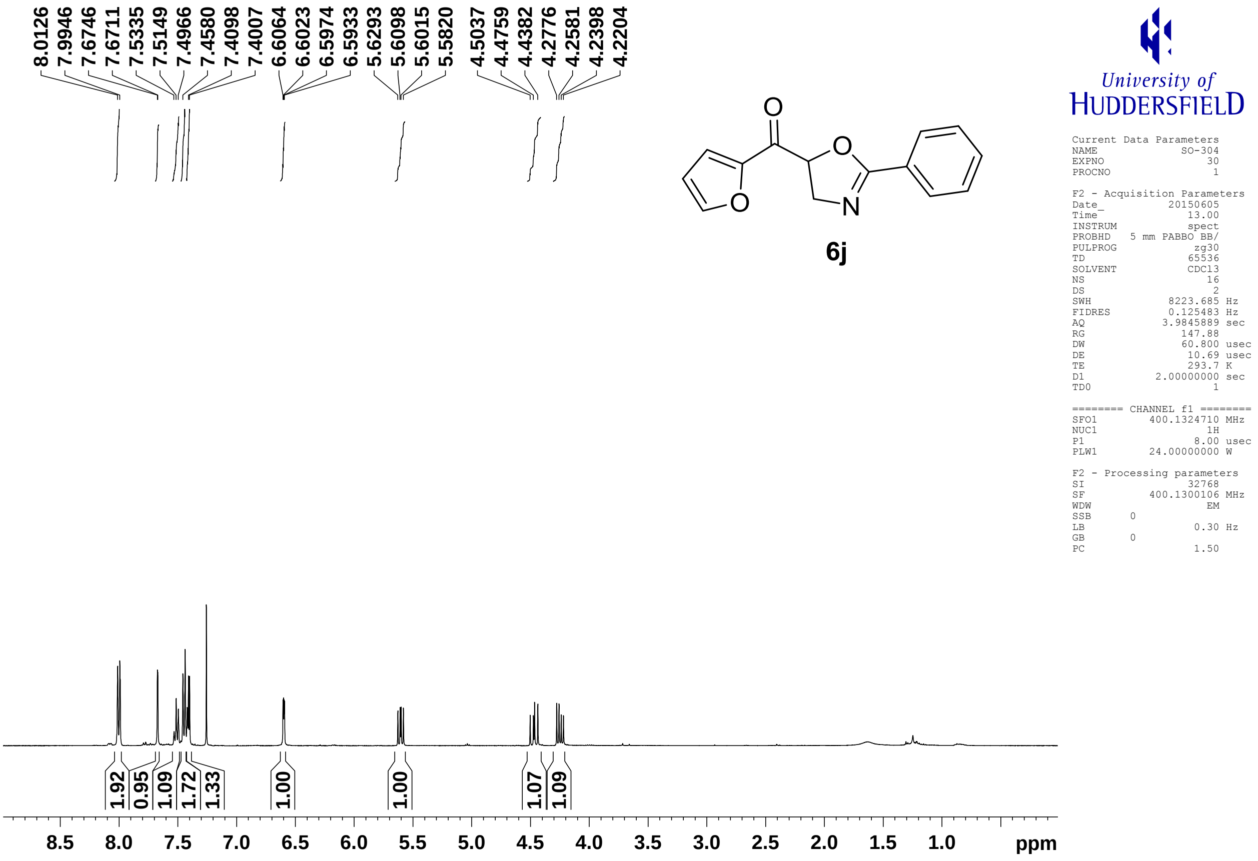
8.0126
7.9946
7.6746
7.6711
7.5335
7.5149
7.4966
7.4580
7.4098
7.4007
6.6064
6.6023
6.5974
6.5933
5.6293
5.6098
5.6015
5.5820
4.5037
4.4759
4.4382
4.2776
4.2581
4.2398
4.2204
6j
University of HUDDERSFIELD
Current Data Parameters
NAME SO-304
EXPNO 30
PROCNO 1
F2 - Acquisition Parameters
Date_ 20150605
Time 13.00
INSTRUM spect
PROBHD 5 mm PABBO BB/
PULPROG zg30
TD 65536
SOLVENT CDCl3
NS 16
DS 2
SWH 8223.685 Hz
FIDRES 0.125483 Hz
AQ 3.9845889 sec
RG 147.88
DW 60.800 usec
DE 10.69 usec
TE 293.7 K
D1 2.00000000 sec
TD0 1
======== CHANNEL f1 ========
SFO1 400.1324710 MHz
NUC1 1H
P1 8.00 usec
PLW1 24.00000000 W
F2 - Processing parameters
SI 32768
SF 400.1300106 MHz
WDW EM
SSB 0
LB 0.30 Hz
GB 0
PC 1.50
1.92
0.95
1.09
1.72
1.33
1.00
1.00
1.07
1.09
8.5 8.0 7.5 7.0 6.5 6.0 5.5 5.0 4.5 4.0 3.5 3.0 2.5 2.0 1.5 1.0 ppm

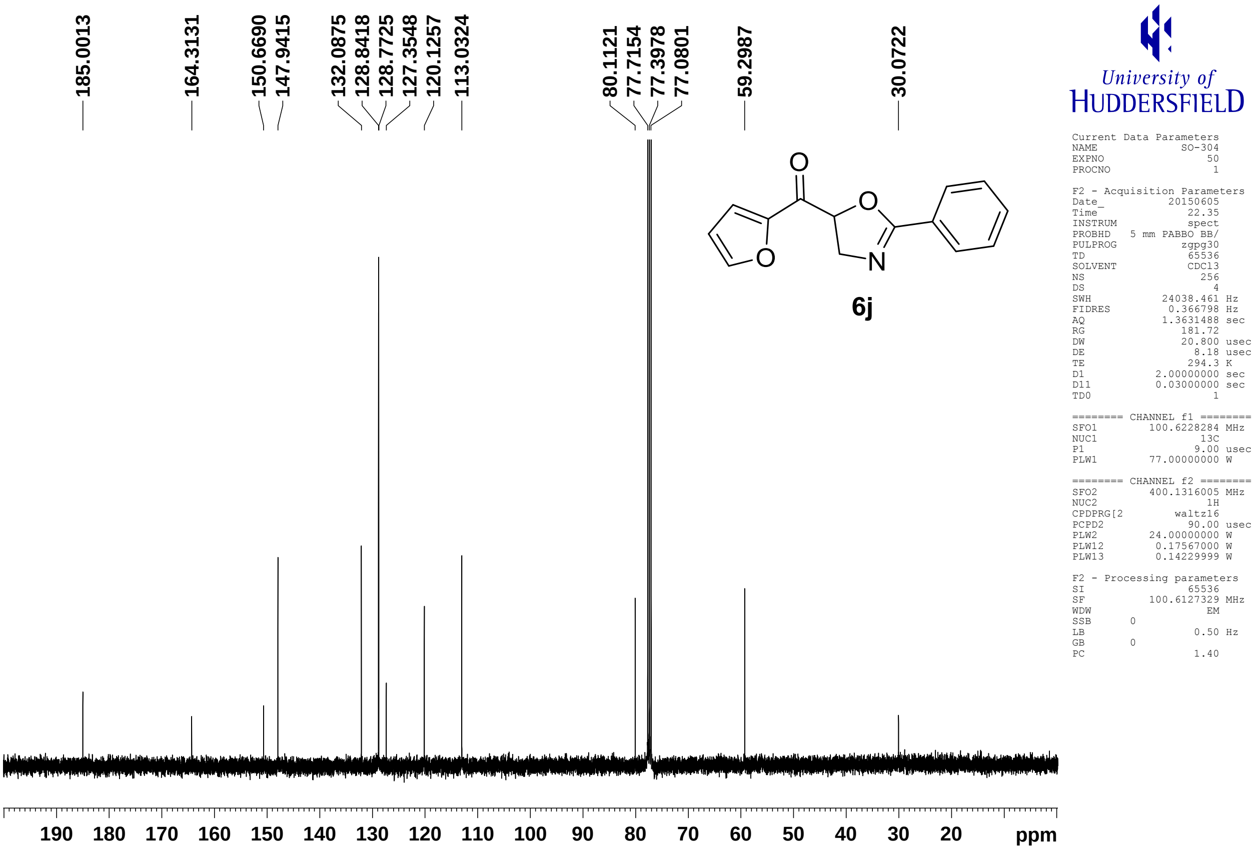
185.0013
164.3131
150.6690
147.9415
132.0875
128.8418
128.7725
127.3548
120.1257
113.0324
80.1121
77.7154
77.3978
77.0801
59.2987
30.0722
6j
University of HUDDERSFIELD
Current Data Parameters
NAME SO-304
EXPNO 50
PROCNO 1
F2 - Acquisition Parameters
Date_ 20150605
Time 22.35
INSTRUM spect
PROBHD 5 mm PABBO BB/
PULPROG zgpg30
TD 65536
SOLVENT CDCl3
NS 256
DS 4
SWH 24038.461 Hz
FIDRES 0.366798 Hz
AQ 1.3631488 sec
RG 181.72
DW 20.800 usec
DE 8.18 usec
TE 294.3 K
D1 2.00000000 sec
D11 0.03000000 sec
TD0 1
======== CHANNEL f1 ========
SFO1 100.6228284 MHz
NUC1 13C
P1 9.00 usec
PLW1 77.00000000 W
======== CHANNEL f2 ========
SFO2 400.1316005 MHz
NUC2 1H
CPDPRG[2 waltz16
PCPD2 90.00 usec
PLW2 24.00000000 W
PLW12 0.17567000 W
PLW13 0.14229999 W
F2 - Processing parameters
SI 65536
SF 100.6127329 MHz
WDW EM
SSB 0
LB 0.50 Hz
GB 0
PC 1.40
190 180 170 160 150 140 130 120 110 100 90 80 70 60 50 40 30 20 ppm

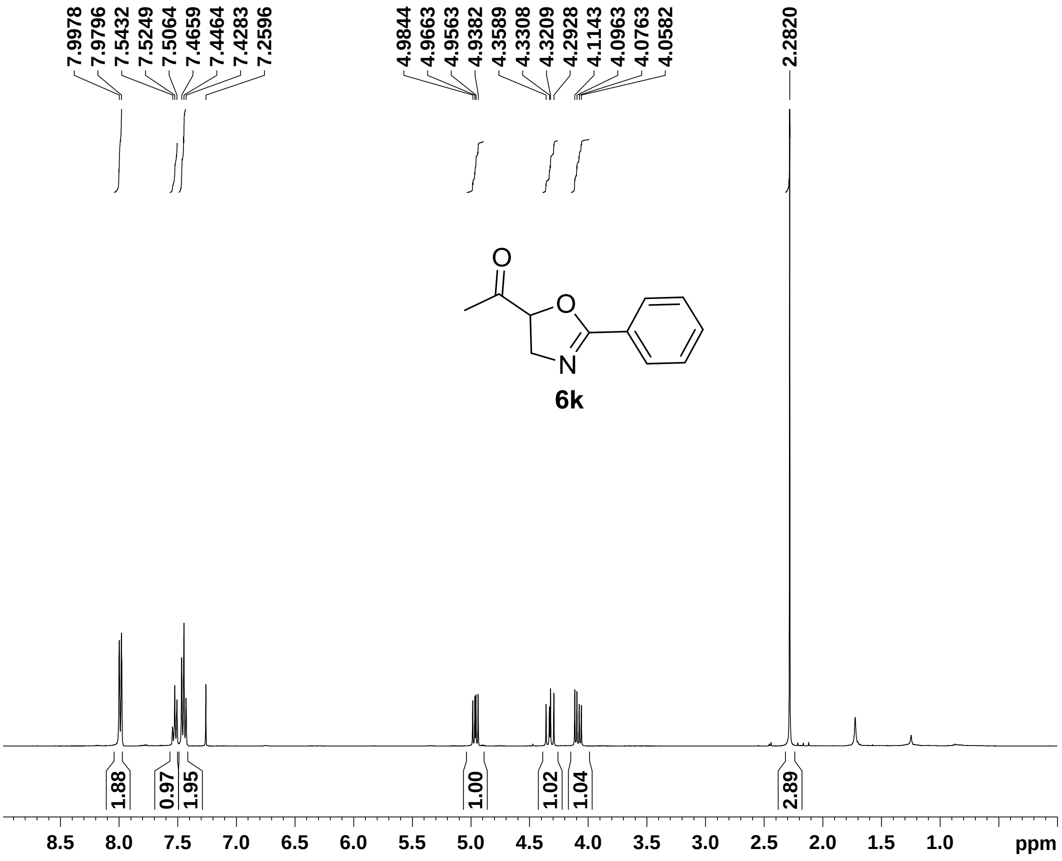

University of
HUDDERSFIELD

```
Current Data Parameters
NAME              SO-406
EXPNO                 20
PROCNO                 1

F2 - Acquisition Parameters
Date_           20151105
Time                9.30
INSTRUM            spect
PROBHD   5 mm PABBO BB/
PULPROG             zg30
TD                 65536
SOLVENT            CDCl3
NS                    16
DS                     2
SWH             8223.685 Hz
FIDRES          0.125483 Hz
AQ             3.9845889 sec
RG                104.33
DW                60.800 usec
DE                 10.69 usec
TE                 293.4 K
D1            2.00000000 sec
TD0                    1

======== CHANNEL f1 ========
SFO1         400.1324710 MHz
NUC1                  1H
P1                  8.00 usec
PLW1         24.00000000 W

F2 - Processing parameters
SI                 32768
SF           400.1300098 MHz
WDW                   EM
SSB      0
LB                  0.30 Hz
GB       0
PC                  1.50
```

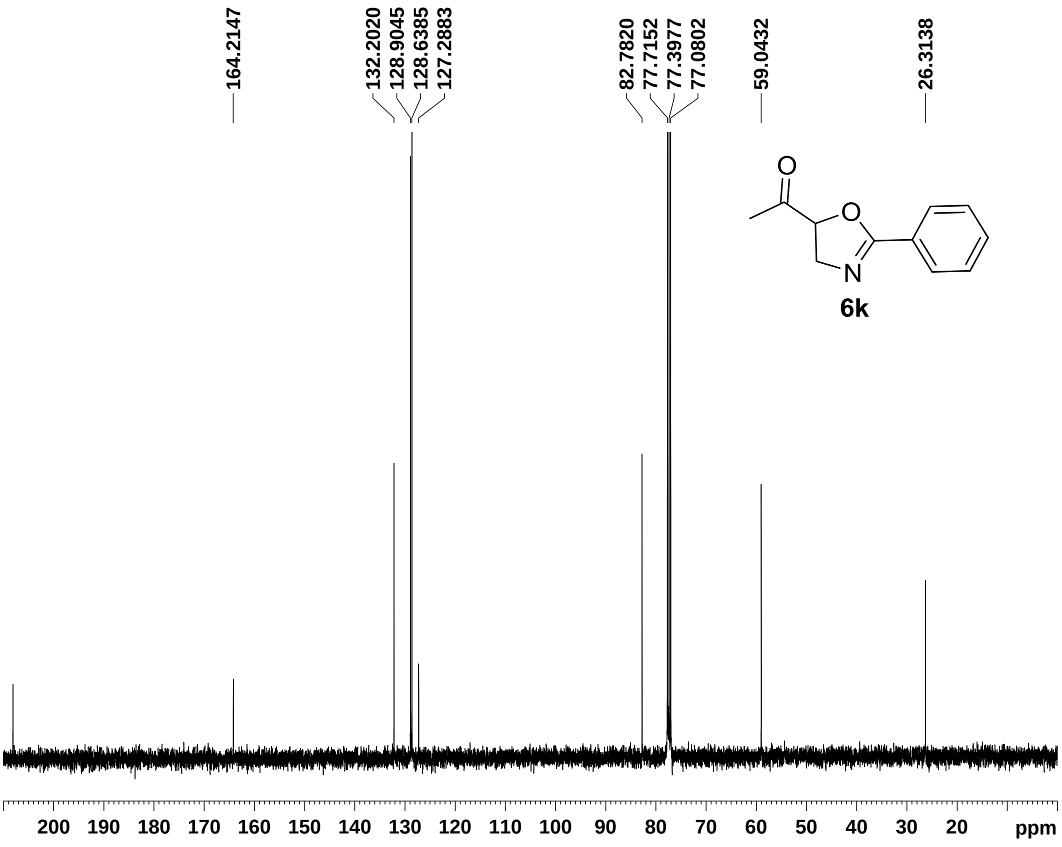

University of
HUDDERSFIELD

```
Current Data Parameters
NAME              SO-406
EXPNO                 21
PROCNO                 1

F2 - Acquisition Parameters
Date_           20151105
Time                9.45
INSTRUM            spect
PROBHD   5 mm PABBO BB/
PULPROG           zgpg30
TD                 65536
SOLVENT            CDCl3
NS                   256
DS                     4
SWH            24038.461 Hz
FIDRES          0.366798 Hz
AQ             1.3631488 sec
RG                181.72
DW                20.800 usec
DE                  8.18 usec
TE                 293.9 K
D1            2.00000000 sec
D11           0.03000000 sec
TD0                    1

======== CHANNEL f1 ========
SFO1         100.6228303 MHz
NUC1                 13C
P1                  9.00 usec
PLW1         77.00000000 W

======== CHANNEL f2 ========
SFO2         400.1316005 MHz
NUC2                  1H
CPDPRG[2         waltz16
PCPD2              90.00 usec
PLW2         24.00000000 W
PLW12         0.17567000 W
PLW13         0.14229999 W

F2 - Processing parameters
SI                 65536
SF           100.6127332 MHz
WDW                   EM
SSB      0
LB                  1.00 Hz
GB       0
PC                  1.40
```

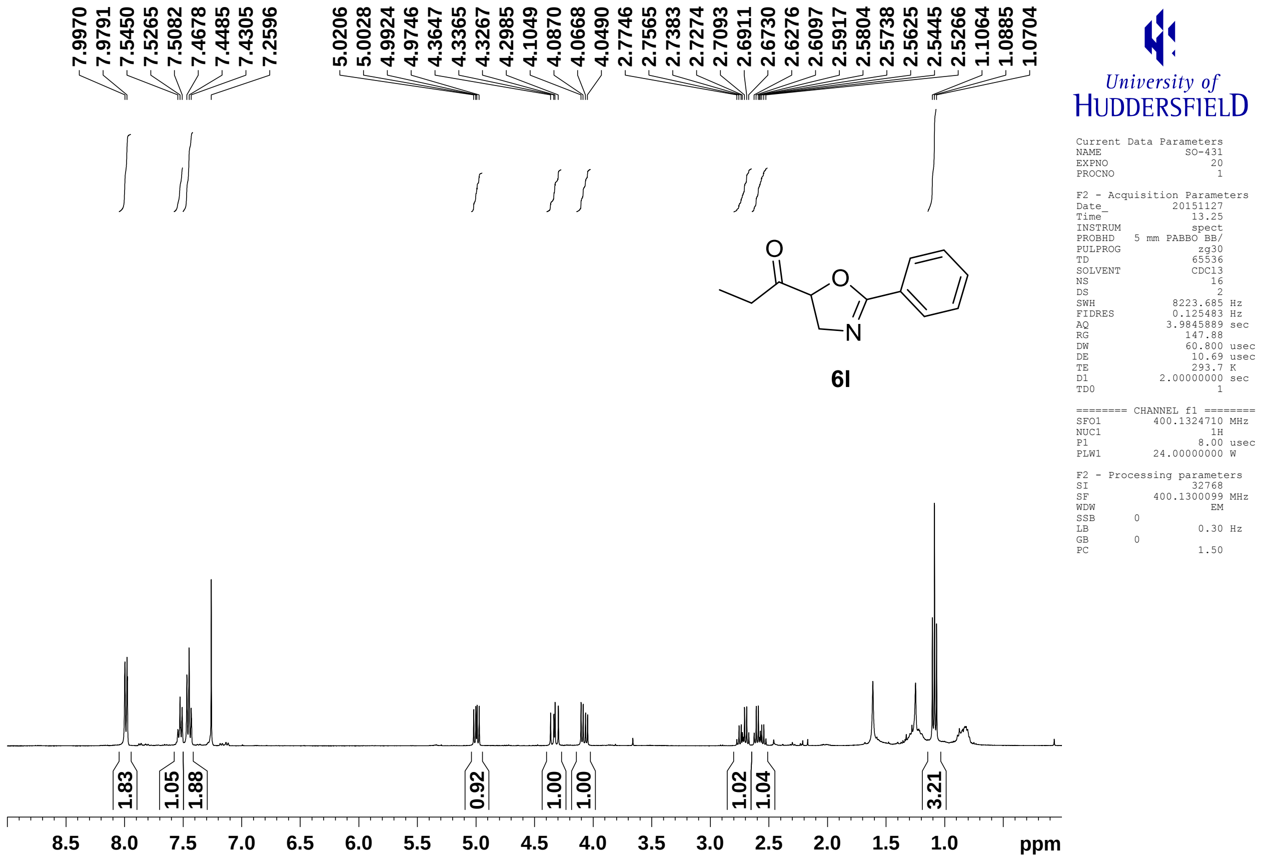
7.9970
7.9791
7.5450
7.5265
7.5082
7.4678
7.4485
7.4305
7.2596
5.0206
5.0028
4.9924
4.9746
4.3647
4.3365
4.3267
4.2985
4.1049
4.0870
4.0668
4.0490
2.7746
2.7565
2.7383
2.7274
2.7093
2.6911
2.6730
2.6276
2.6097
2.5917
2.5804
2.5738
2.5625
2.5445
2.5266
1.1064
1.0885
1.0704
University of HUDDERSFIELD
Current Data Parameters
NAME SO-431
EXPNO 20
PROCNO 1
F2 - Acquisition Parameters
Date_ 20151127
Time 13.25
INSTRUM spect
PROBHD 5 mm PABBO BB/
PULPROG zg30
TD 65536
SOLVENT CDCl3
NS 16
DS 2
SWH 8223.685 Hz
FIDRES 0.125483 Hz
AQ 3.9845889 sec
RG 147.88
DW 60.800 usec
DE 10.69 usec
TE 293.7 K
D1 2.00000000 sec
TD0 1
======== CHANNEL f1 ========
SFO1 400.1324710 MHz
NUC1 1H
P1 8.00 usec
PLW1 24.00000000 W
F2 - Processing parameters
SI 32768
SF 400.1300099 MHz
WDW EM
SSB 0
LB 0.30 Hz
GB 0
PC 1.50
O
O
N
6l
1.83
1.05
1.88
0.92
1.00
1.00
1.02
1.04
3.21
8.5
8.0
7.5
7.0
6.5
6.0
5.5
5.0
4.5
4.0
3.5
3.0
2.5
2.0
1.5
1.0
ppm

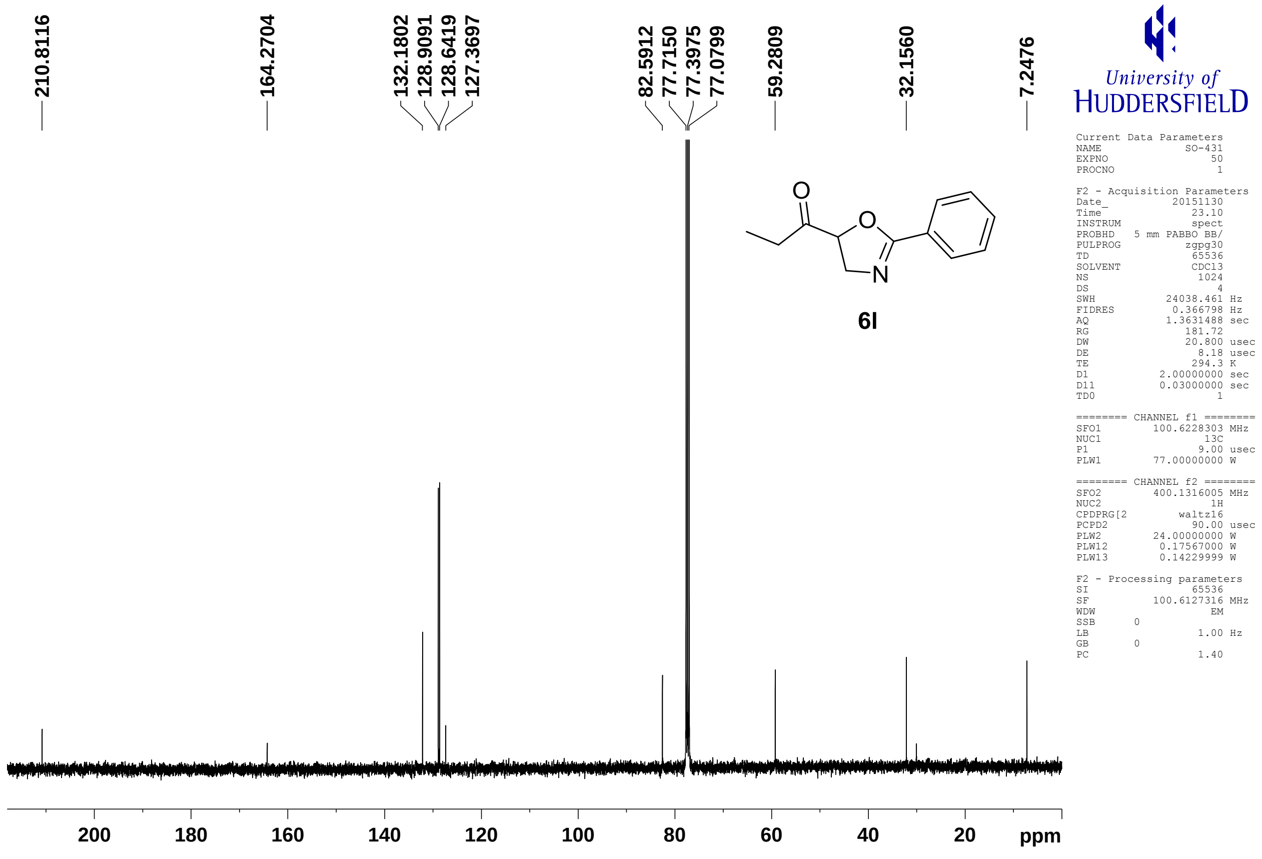
210.8116
164.2704
132.1802
128.9091
128.6419
127.3697
82.5912
77.7150
77.3975
77.0799
59.2809
32.1560
7.2476
University of HUDDERSFIELD
Current Data Parameters
NAME SO-431
EXPNO 50
PROCNO 1
F2 - Acquisition Parameters
Date_ 20151130
Time 23.10
INSTRUM spect
PROBHD 5 mm PABBO BB/
PULPROG zgpg30
TD 65536
SOLVENT CDCl3
NS 1024
DS 4
SWH 24038.461 Hz
FIDRES 0.366798 Hz
AQ 1.3631488 sec
RG 181.72
DW 20.800 usec
DE 8.18 usec
TE 294.3 K
D1 2.00000000 sec
D11 0.03000000 sec
TD0 1
======== CHANNEL f1 ========
SFO1 100.6228303 MHz
NUC1 13C
P1 9.00 usec
PLW1 77.00000000 W
======== CHANNEL f2 ========
SFO2 400.1316005 MHz
NUC2 1H
CPDPRG[2 waltz16
PCPD2 90.00 usec
PLW2 24.00000000 W
PLW12 0.17567000 W
PLW13 0.14229999 W
F2 - Processing parameters
SI 65536
SF 100.6127316 MHz
WDW EM
SSB 0
LB 1.00 Hz
GB 0
PC 1.40
O
O
N
6l
200
180
160
140
120
100
80
60
40
20
ppm

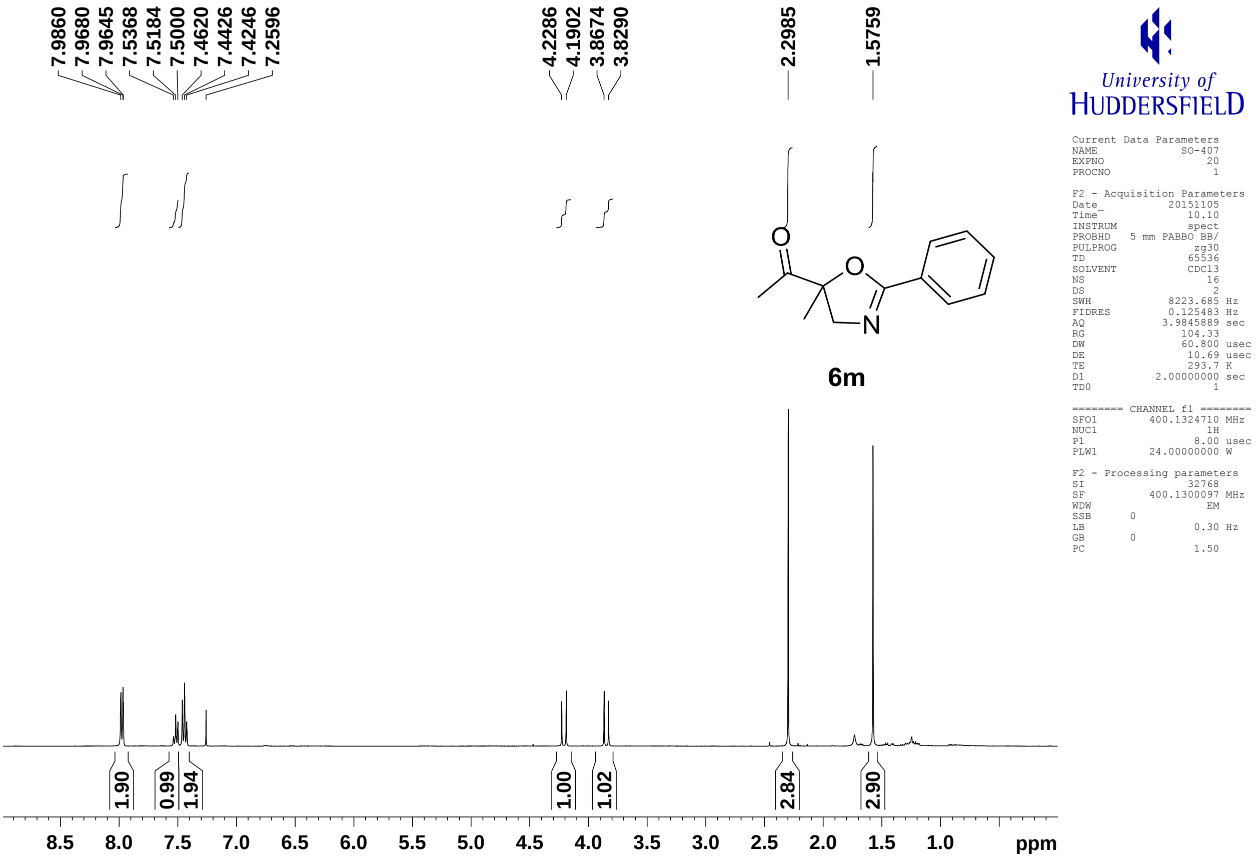
7.9860
7.9680
7.9645
7.5368
7.5184
7.5000
7.4620
7.4426
7.4246
7.2596
4.2286
4.1902
3.8674
3.8290
2.2985
1.5759
O
O
N
6m
1.90
0.99
1.94
1.00
1.02
2.84
2.90
8.5 8.0 7.5 7.0 6.5 6.0 5.5 5.0 4.5 4.0 3.5 3.0 2.5 2.0 1.5 1.0 ppm
University of HUDDERSFIELD
Current Data Parameters
NAME SO-407
EXPNO 20
PROCNO 1
F2 - Acquisition Parameters
Date_ 20151105
Time 10.10
INSTRUM spect
PROBHD 5 mm PABBO BB/
PULPROG zg30
TD 65536
SOLVENT CDCl3
NS 16
DS 2
SWH 8223.685 Hz
FIDRES 0.125483 Hz
AQ 3.9845889 sec
RG 104.33
DW 60.800 usec
DE 10.69 usec
TE 293.7 K
D1 2.00000000 sec
TD0 1
======== CHANNEL f1 ========
SFO1 400.1324710 MHz
NUC1 1H
P1 8.00 usec
PLW1 24.00000000 W
F2 - Processing parameters
SI 32768
SF 400.1300097 MHz
WDW EM
SSB 0
LB 0.30 Hz
GB 0
PC 1.50

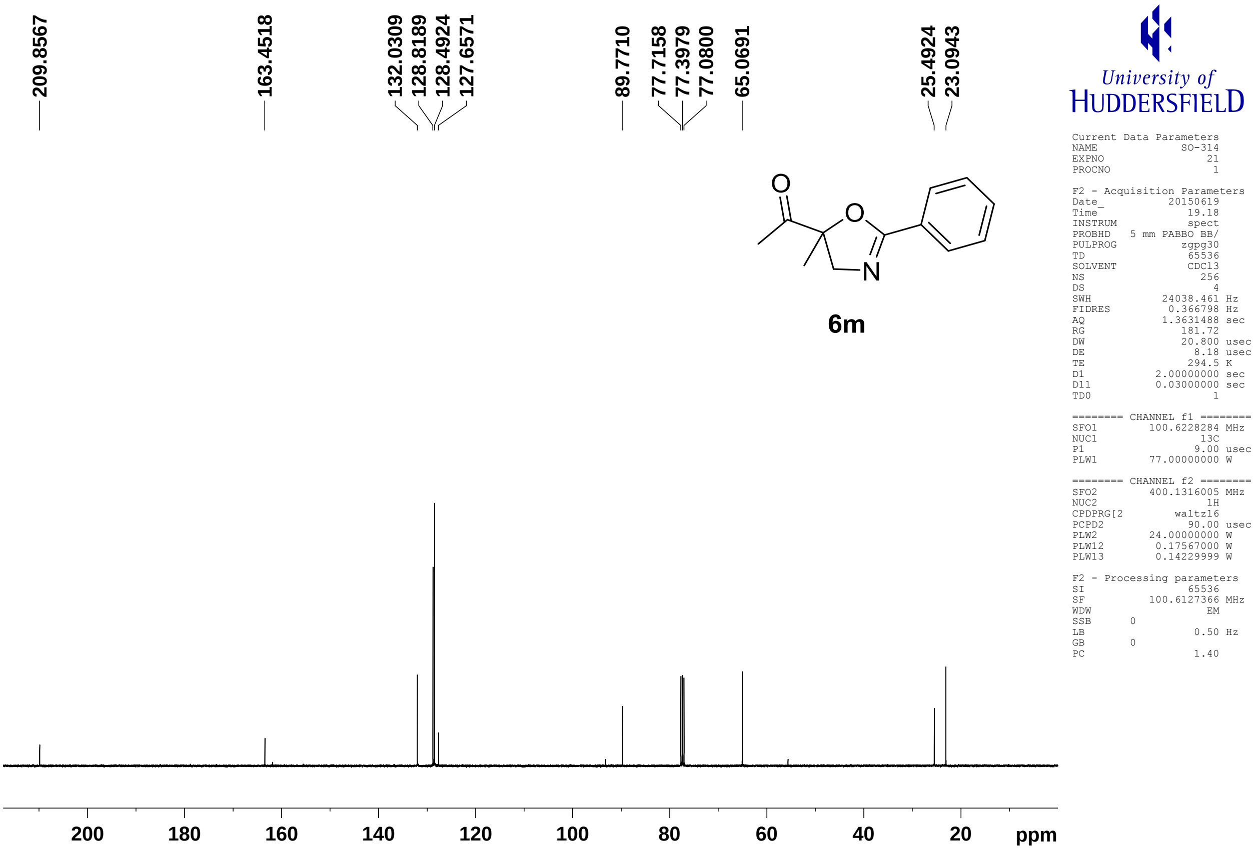
209.8567
163.4518
132.0309
128.8189
128.4924
127.6571
89.7710
77.7158
77.3979
77.0800
65.0691
25.4924
23.0943
O
O
N
6m
200 180 160 140 120 100 80 60 40 20 ppm
University of HUDDERSFIELD
Current Data Parameters
NAME SO-314
EXPNO 21
PROCNO 1
F2 - Acquisition Parameters
Date_ 20150619
Time 19.18
INSTRUM spect
PROBHD 5 mm PABBO BB/
PULPROG zgpg30
TD 65536
SOLVENT CDCl3
NS 256
DS 4
SWH 24038.461 Hz
FIDRES 0.366798 Hz
AQ 1.3631488 sec
RG 181.72
DW 20.800 usec
DE 8.18 usec
TE 294.5 K
D1 2.00000000 sec
D11 0.03000000 sec
TD0 1
======== CHANNEL f1 ========
SFO1 100.6228284 MHz
NUC1 13C
P1 9.00 usec
PLW1 77.00000000 W
======== CHANNEL f2 ========
SFO2 400.1316005 MHz
NUC2 1H
CPDPRG[2 waltz16
PCPD2 90.00 usec
PLW2 24.00000000 W
PLW12 0.17567000 W
PLW13 0.14229999 W
F2 - Processing parameters
SI 65536
SF 100.6127366 MHz
WDW EM
SSB 0
LB 0.50 Hz
GB 0
PC 1.40

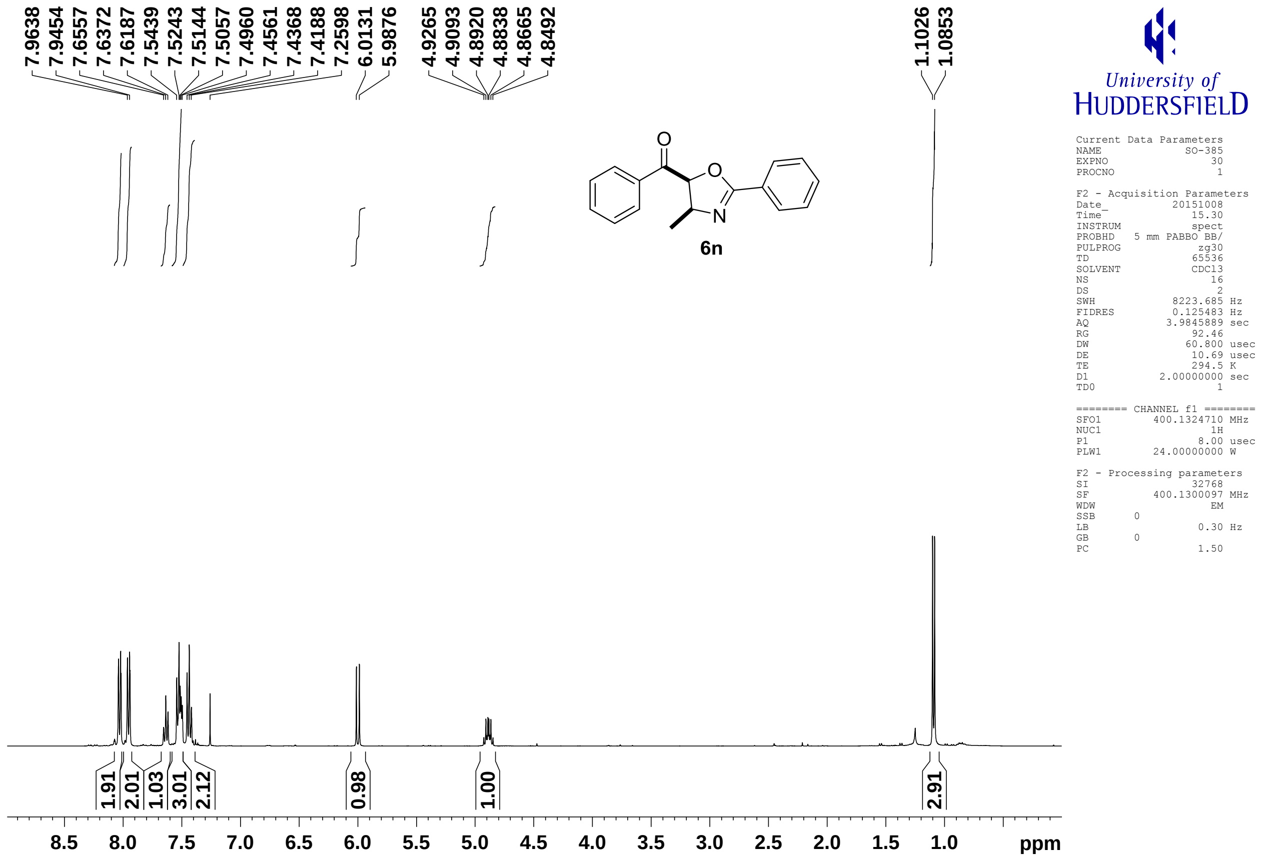
7.9638
7.9454
7.6557
7.6372
7.6187
7.5439
7.5243
7.5144
7.5057
7.4960
7.4561
7.4368
7.4188
7.2598
6.0131
5.9876
4.9265
4.9093
4.8920
4.8838
4.8665
4.8492
1.1026
1.0853
6n
1.91
2.01
1.03
3.01
2.12
0.98
1.00
2.91
8.5 8.0 7.5 7.0 6.5 6.0 5.5 5.0 4.5 4.0 3.5 3.0 2.5 2.0 1.5 1.0 ppm
University of HUDDERSFIELD
Current Data Parameters
NAME SO-385
EXPNO 30
PROCNO 1
F2 - Acquisition Parameters
Date_ 20151008
Time 15.30
INSTRUM spect
PROBHD 5 mm PABBO BB/
PULPROG zg30
TD 65536
SOLVENT CDCl3
NS 16
DS 2
SWH 8223.685 Hz
FIDRES 0.125483 Hz
AQ 3.9845889 sec
RG 92.46
DW 60.800 usec
DE 10.69 usec
TE 294.5 K
D1 2.00000000 sec
TD0 1
======== CHANNEL f1 ========
SFO1 400.1324710 MHz
NUC1 1H
P1 8.00 usec
PLW1 24.00000000 W
F2 - Processing parameters
SI 32768
SF 400.1300097 MHz
WDW EM
SSB 0
LB 0.30 Hz
GB 0
PC 1.50

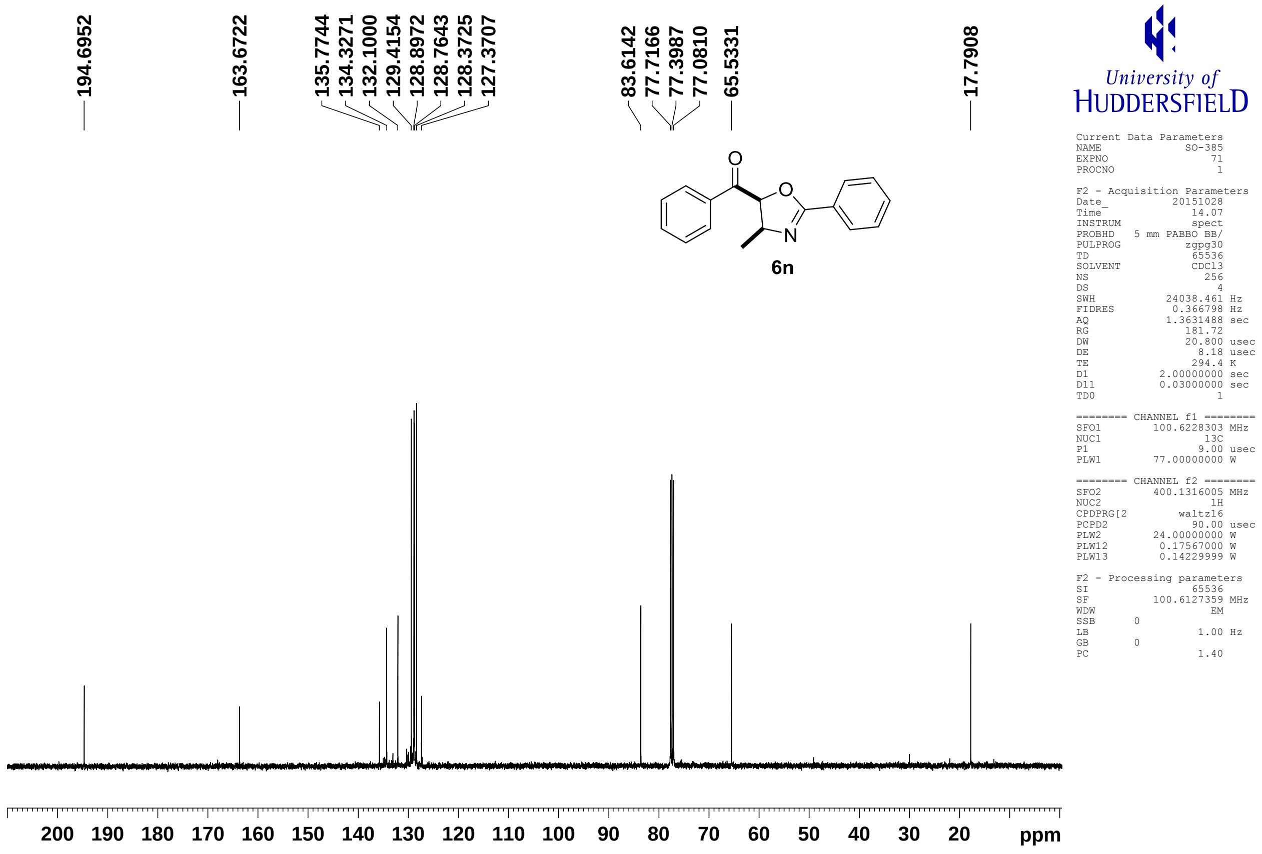
194.6952
163.6722
135.7744
134.3271
132.1000
129.4154
128.8972
128.7643
128.3725
127.3707
83.6142
77.7166
77.3987
77.0810
65.5331
17.7908
6n
200 190 180 170 160 150 140 130 120 110 100 90 80 70 60 50 40 30 20 ppm
University of HUDDERSFIELD
Current Data Parameters
NAME SO-385
EXPNO 71
PROCNO 1
F2 - Acquisition Parameters
Date_ 20151028
Time 14.07
INSTRUM spect
PROBHD 5 mm PABBO BB/
PULPROG zgpg30
TD 65536
SOLVENT CDCl3
NS 256
DS 4
SWH 24038.461 Hz
FIDRES 0.366798 Hz
AQ 1.3631488 sec
RG 181.72
DW 20.800 usec
DE 8.18 usec
TE 294.4 K
D1 2.00000000 sec
D11 0.03000000 sec
TD0 1
======== CHANNEL f1 ========
SFO1 100.6228303 MHz
NUC1 13C
P1 9.00 usec
PLW1 77.00000000 W
======== CHANNEL f2 ========
SFO2 400.1316005 MHz
NUC2 1H
CPDPRG[2 waltz16
PCPD2 90.00 usec
PLW2 24.00000000 W
PLW12 0.17567000 W
PLW13 0.14229999 W
F2 - Processing parameters
SI 65536
SF 100.6127359 MHz
WDW EM
SSB 0
LB 1.00 Hz
GB 0
PC 1.40

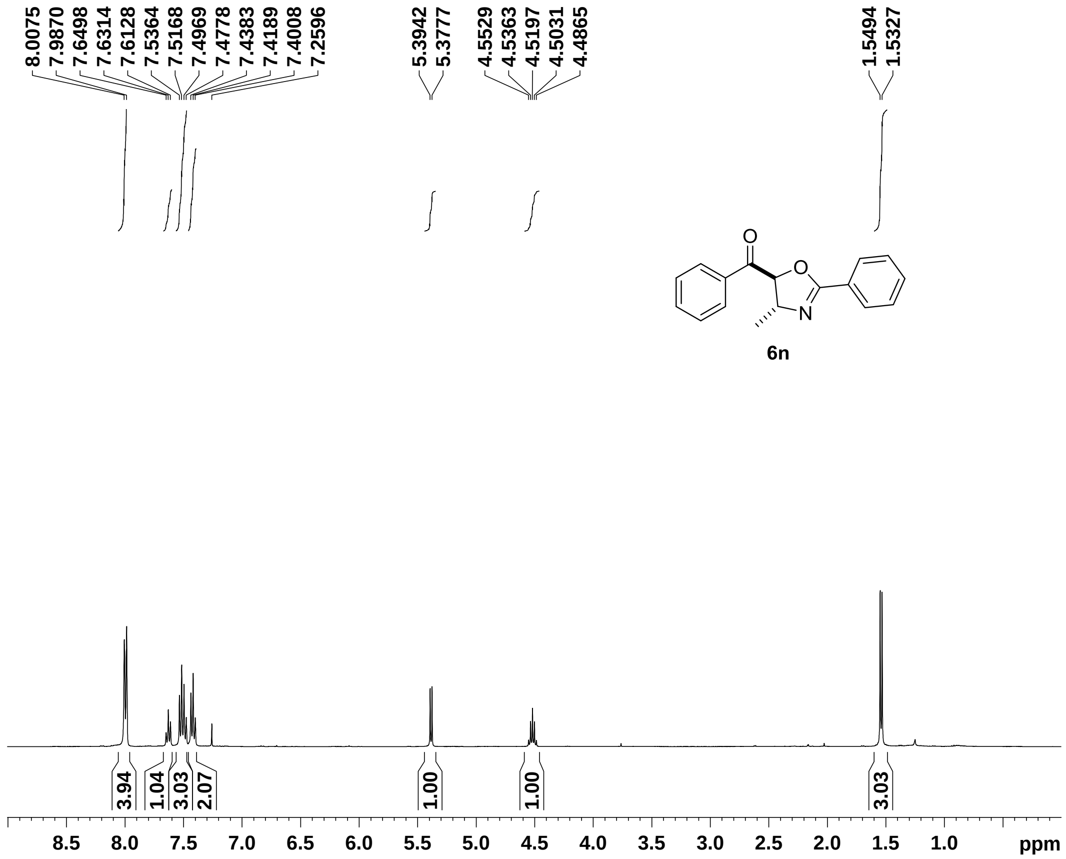

University of HUDDERSFIELD

```
Current Data Parameters
NAME              SO-385
EXPNO                 60
PROCNO                 1

F2 - Acquisition Parameters
Date_           20151028
Time               13.32
INSTRUM            spect
PROBHD   5 mm PABBO BB/
PULPROG             zg30
TD                 65536
SOLVENT            CDCl3
NS                    16
DS                     2
SWH             8223.685 Hz
FIDRES          0.125483 Hz
AQ             3.9845889 sec
RG                 50.36
DW                60.800 usec
DE                 10.69 usec
TE                 294.1 K
D1            2.00000000 sec
TD0                    1

======== CHANNEL f1 ========
SFO1         400.1324710 MHz
NUC1                  1H
P1                  8.00 usec
PLW1         24.00000000 W

F2 - Processing parameters
SI                 32768
SF           400.1300097 MHz
WDW                   EM
SSB      0
LB                  0.30 Hz
GB       0
PC                  1.50
```

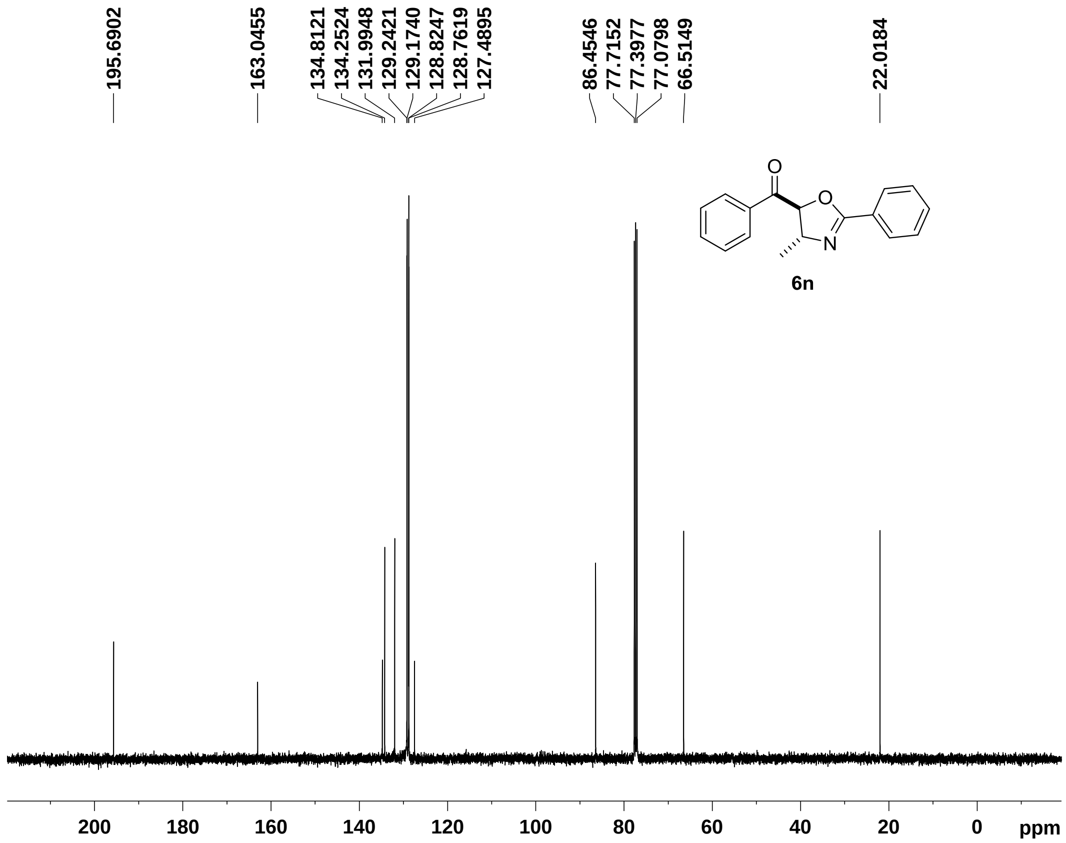

University of HUDDERSFIELD

```
Current Data Parameters
NAME              SO-385
EXPNO                 61
PROCNO                 1

F2 - Acquisition Parameters
Date_           20151028
Time               13.47
INSTRUM            spect
PROBHD   5 mm PABBO BB/
PULPROG           zgpg30
TD                 65536
SOLVENT            CDCl3
NS                   256
DS                     4
SWH            24038.461 Hz
FIDRES          0.366798 Hz
AQ             1.3631488 sec
RG                181.72
DW                20.800 usec
DE                  8.18 usec
TE                 294.4 K
D1            2.00000000 sec
D11           0.03000000 sec
TD0                    1

======== CHANNEL f1 ========
SFO1         100.6228303 MHz
NUC1                 13C
P1                  9.00 usec
PLW1         77.00000000 W

======== CHANNEL f2 ========
SFO2         400.1316005 MHz
NUC2                  1H
CPDPRG[2         waltz16
PCPD2              90.00 usec
PLW2         24.00000000 W
PLW12         0.17567000 W
PLW13         0.14229999 W

F2 - Processing parameters
SI                 65536
SF           100.6127352 MHz
WDW                   EM
SSB      0
LB                  1.00 Hz
GB       0
PC                  1.40
```

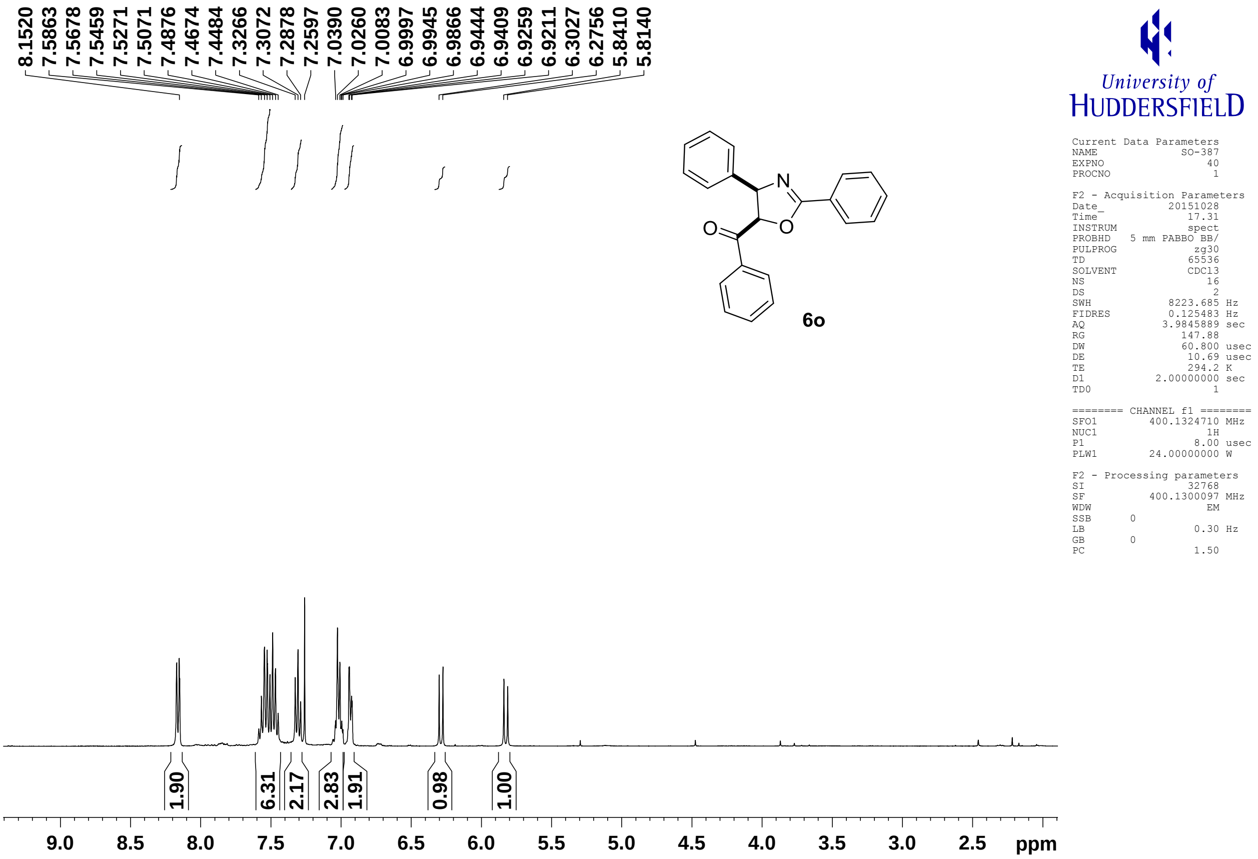
8.1520 7.5863 7.5678 7.5459 7.5271 7.5071 7.4876 7.4674 7.4484 7.3266 7.3072 7.2878 7.2597 7.0390 7.0260 7.0083 6.9997 6.9945 6.9866 6.9444 6.9409 6.9259 6.9211 6.3027 6.2756 5.8410 5.8140
University of HUDDERSFIELD
Current Data Parameters
NAME SO-387
EXPNO 40
PROCNO 1
F2 - Acquisition Parameters
Date_ 20151028
Time 17.31
INSTRUM spect
PROBHD 5 mm PABBO BB/
PULPROG zg30
TD 65536
SOLVENT CDCl3
NS 16
DS 2
SWH 8223.685 Hz
FIDRES 0.125483 Hz
AQ 3.9845889 sec
RG 147.88
DW 60.800 usec
DE 10.69 usec
TE 294.2 K
D1 2.00000000 sec
TD0 1
======== CHANNEL f1 ========
SFO1 400.1324710 MHz
NUC1 1H
P1 8.00 usec
PLW1 24.00000000 W
F2 - Processing parameters
SI 32768
SF 400.1300097 MHz
WDW EM
SSB 0
LB 0.30 Hz
GB 0
PC 1.50
6o
1.90 6.31 2.17 2.83 1.91 0.98 1.00
9.0 8.5 8.0 7.5 7.0 6.5 6.0 5.5 5.0 4.5 4.0 3.5 3.0 2.5 ppm

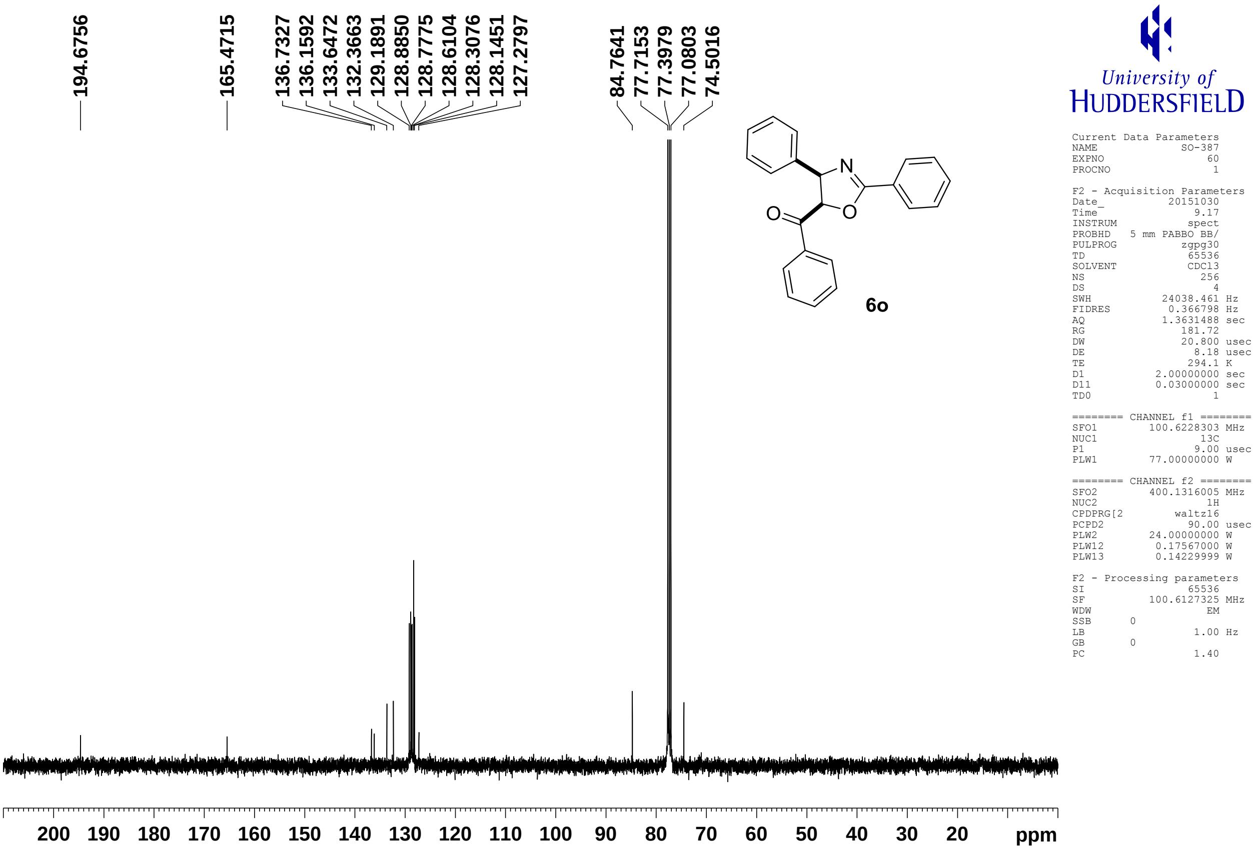
194.6756 165.4715 136.7327 136.1592 133.6472 132.3663 129.1891 128.8850 128.7775 128.6104 128.3076 128.1451 127.2797 84.7641 77.7153 77.3979 77.0803 74.5016
University of HUDDERSFIELD
Current Data Parameters
NAME SO-387
EXPNO 60
PROCNO 1
F2 - Acquisition Parameters
Date_ 20151030
Time 9.17
INSTRUM spect
PROBHD 5 mm PABBO BB/
PULPROG zgpg30
TD 65536
SOLVENT CDCl3
NS 256
DS 4
SWH 24038.461 Hz
FIDRES 0.366798 Hz
AQ 1.3631488 sec
RG 181.72
DW 20.800 usec
DE 8.18 usec
TE 294.1 K
D1 2.00000000 sec
D11 0.03000000 sec
TD0 1
======== CHANNEL f1 ========
SFO1 100.6228303 MHz
NUC1 13C
P1 9.00 usec
PLW1 77.00000000 W
======== CHANNEL f2 ========
SFO2 400.1316005 MHz
NUC2 1H
CPDPRG[2 waltz16
PCPD2 90.00 usec
PLW2 24.00000000 W
PLW12 0.17567000 W
PLW13 0.14229999 W
F2 - Processing parameters
SI 65536
SF 100.6127325 MHz
WDW EM
SSB 0
LB 1.00 Hz
GB 0
PC 1.40
6o
200 190 180 170 160 150 140 130 120 110 100 90 80 70 60 50 40 30 20 ppm

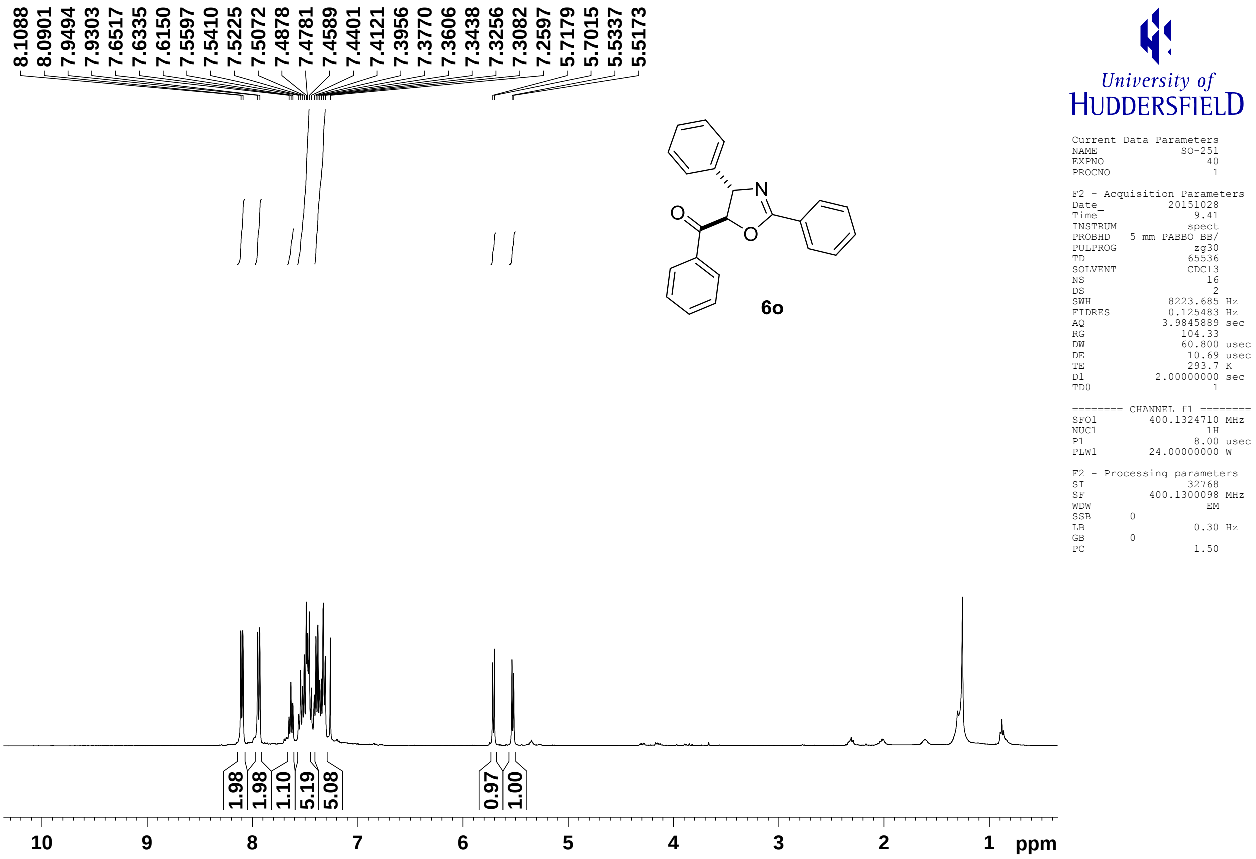
8.1088
8.0901
7.9494
7.9303
7.6517
7.6335
7.6150
7.5597
7.5410
7.5225
7.5072
7.4878
7.4781
7.4589
7.4401
7.4121
7.3956
7.3770
7.3606
7.3438
7.3256
7.3082
7.2597
5.7179
5.7015
5.5337
5.5173
N
O
O
6o
University of HUDDERSFIELD
Current Data Parameters
NAME SO-251
EXPNO 40
PROCNO 1
F2 - Acquisition Parameters
Date_ 20151028
Time 9.41
INSTRUM spect
PROBHD 5 mm PABBO BB/
PULPROG zg30
TD 65536
SOLVENT CDCl3
NS 16
DS 2
SWH 8223.685 Hz
FIDRES 0.125483 Hz
AQ 3.9845889 sec
RG 104.33
DW 60.800 usec
DE 10.69 usec
TE 293.7 K
D1 2.00000000 sec
TD0 1
======== CHANNEL f1 ========
SFO1 400.1324710 MHz
NUC1 1H
P1 8.00 usec
PLW1 24.00000000 W
F2 - Processing parameters
SI 32768
SF 400.1300098 MHz
WDW EM
SSB 0
LB 0.30 Hz
GB 0
PC 1.50
1.98
1.98
1.10
5.19
5.08
0.97
1.00
10 9 8 7 6 5 4 3 2 1 ppm

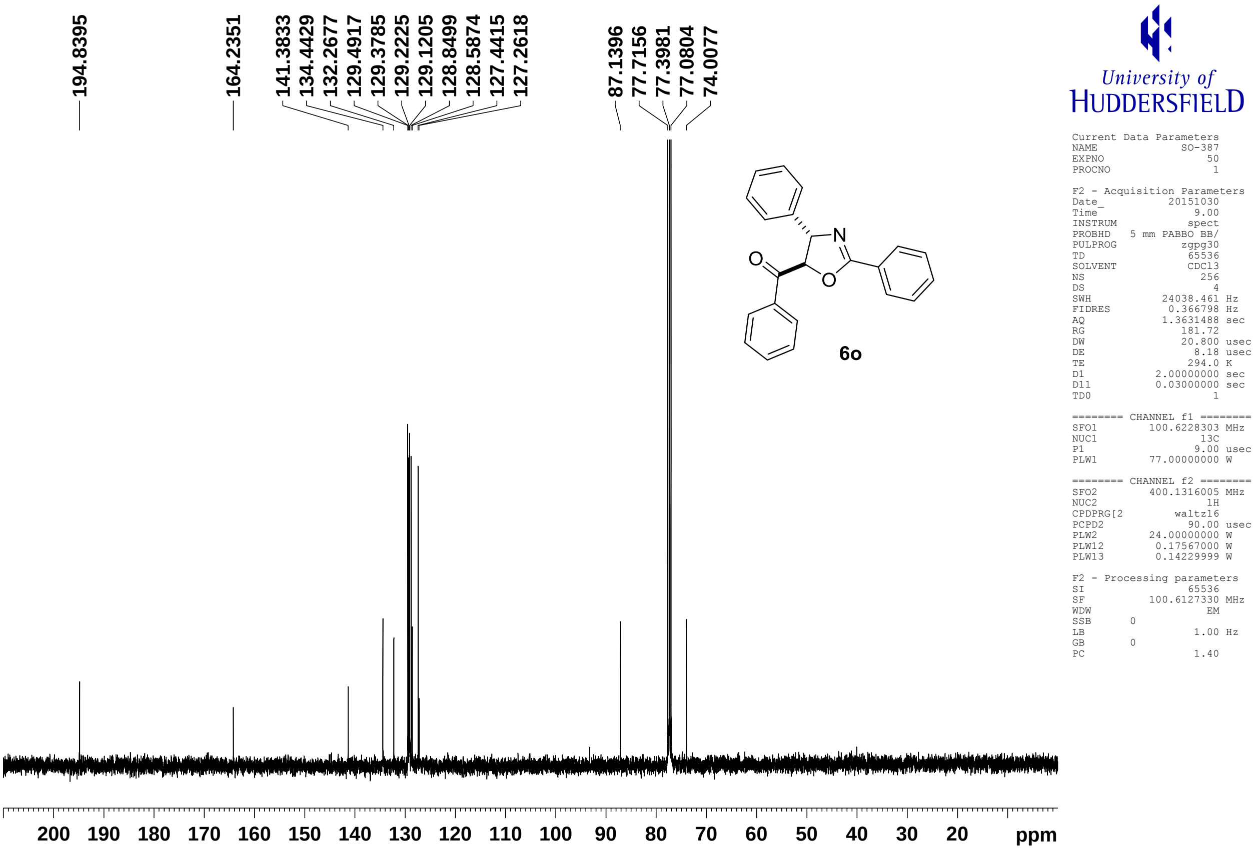
194.8395
164.2351
141.3833
134.4429
132.2677
129.4917
129.3785
129.2225
129.1205
128.8499
128.5874
127.4415
127.2618
87.1396
77.7156
77.3981
77.0804
74.0077
N
O
O
6o
University of HUDDERSFIELD
Current Data Parameters
NAME SO-387
EXPNO 50
PROCNO 1
F2 - Acquisition Parameters
Date_ 20151030
Time 9.00
INSTRUM spect
PROBHD 5 mm PABBO BB/
PULPROG zgpg30
TD 65536
SOLVENT CDCl3
NS 256
DS 4
SWH 24038.461 Hz
FIDRES 0.366798 Hz
AQ 1.3631488 sec
RG 181.72
DW 20.800 usec
DE 8.18 usec
TE 294.0 K
D1 2.00000000 sec
D11 0.03000000 sec
TD0 1
======== CHANNEL f1 ========
SFO1 100.6228303 MHz
NUC1 13C
P1 9.00 usec
PLW1 77.00000000 W
======== CHANNEL f2 ========
SFO2 400.1316005 MHz
NUC2 1H
CPDPRG[2 waltz16
PCPD2 90.00 usec
PLW2 24.00000000 W
PLW12 0.17567000 W
PLW13 0.14229999 W
F2 - Processing parameters
SI 65536
SF 100.6127330 MHz
WDW EM
SSB 0
LB 1.00 Hz
GB 0
PC 1.40
200 190 180 170 160 150 140 130 120 110 100 90 80 70 60 50 40 30 20 ppm

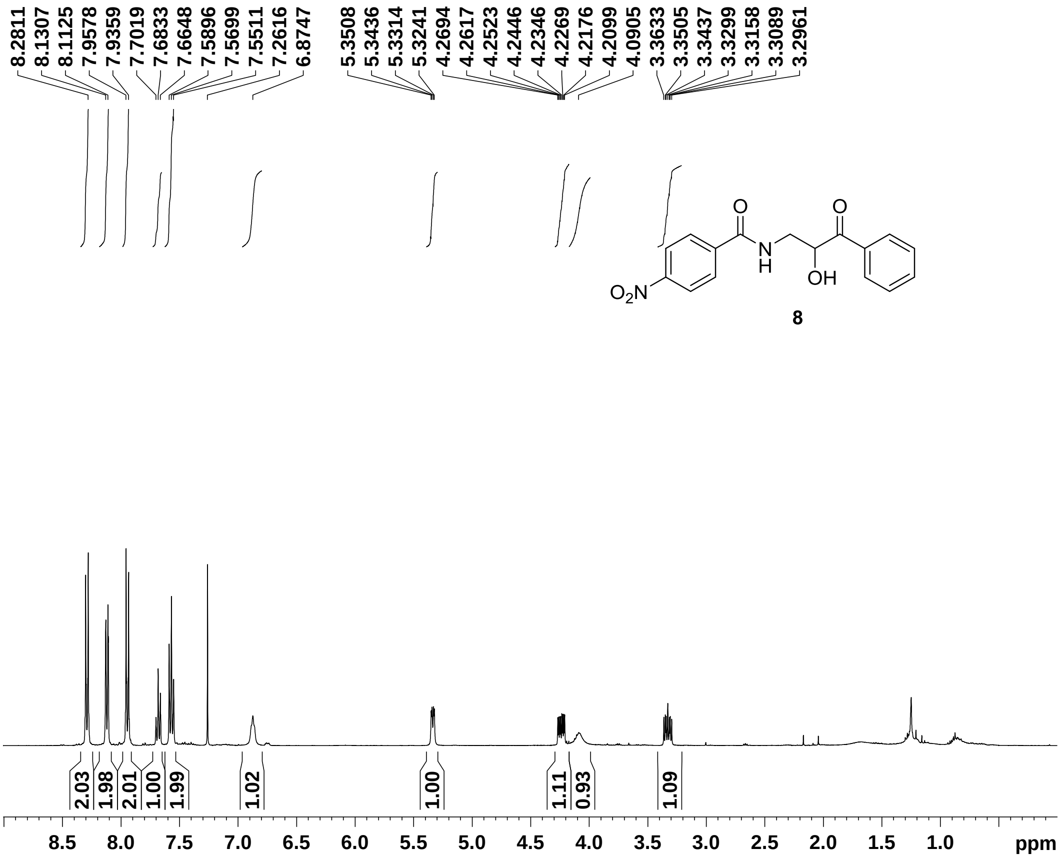

University of HUDDERSFIELD

Current Data Parameters
NAME SO-382
EXPNO 20
PROCNO 1

F2 - Acquisition Parameters
Date_ 20151012
Time 5.26
INSTRUM spect
PROBHD 5 mm PABBO BB/
PULPROG zg30
TD 65536
SOLVENT CDCl3
NS 16
DS 2
SWH 8223.685 Hz
FIDRES 0.125483 Hz
AQ 3.9845889 sec
RG 147.88
DW 60.800 usec
DE 10.69 usec
TE 294.0 K
D1 2.00000000 sec
TD0 1

======== CHANNEL f1 ========
SFO1 400.1324710 MHz
NUC1 1H
P1 8.00 usec
PLW1 24.00000000 W

F2 - Processing parameters
SI 32768
SF 400.1300090 MHz
WDW EM
SSB 0
LB 0.30 Hz
GB 0
PC 1.50

SO-382 C13

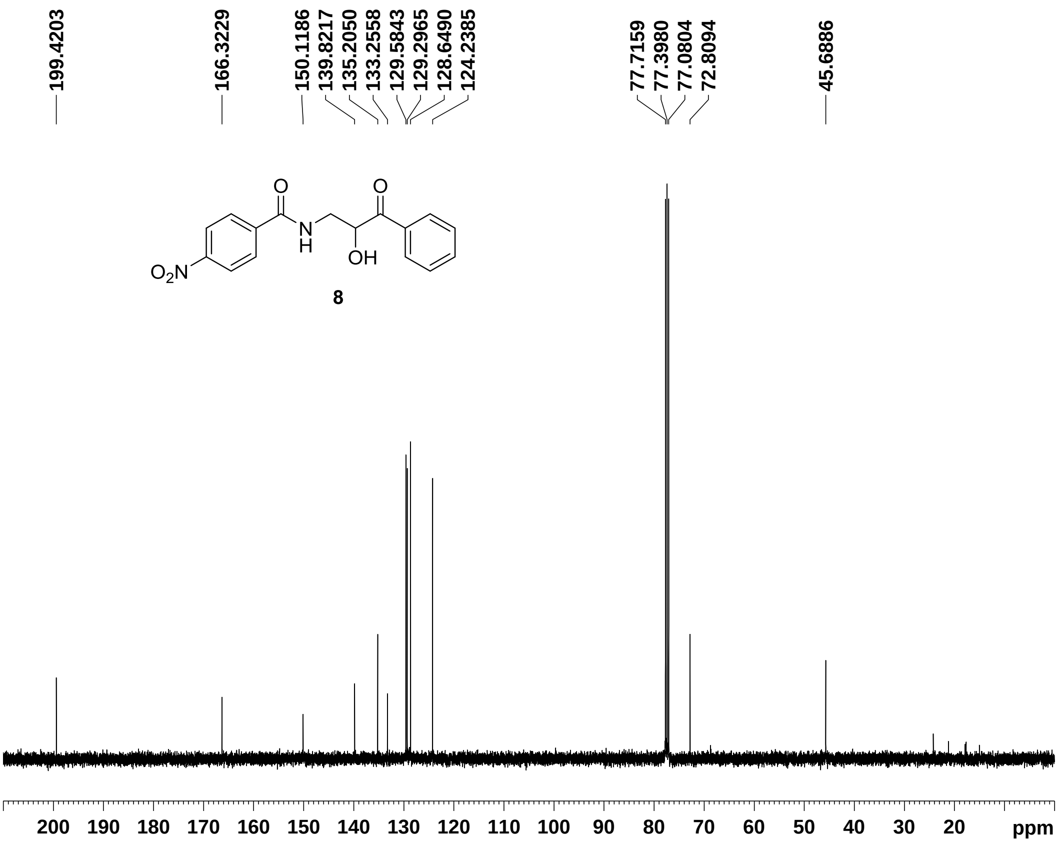

University of HUDDERSFIELD

Current Data Parameters
NAME SO-382
EXPNO 21
PROCNO 1

F2 - Acquisition Parameters
Date_ 20150708
Time 20.45
INSTRUM spect
PROBHD 5 mm PABBO BB/
PULPROG zgpg30
TD 65536
SOLVENT CDCl3
NS 256
DS 4
SWH 24038.461 Hz
FIDRES 0.366798 Hz
AQ 1.3631488 sec
RG 181.72
DW 20.800 usec
DE 8.18 usec
TE 295.0 K
D1 2.00000000 sec
D11 0.03000000 sec
TD0 1

======== CHANNEL f1 ========
SFO1 100.6228284 MHz
NUC1 13C
P1 9.00 usec
PLW1 77.00000000 W

======== CHANNEL f2 ========
SFO2 400.1316005 MHz
NUC2 1H
CPDPRG[2 waltz16
PCPD2 90.00 usec
PLW2 24.00000000 W
PLW12 0.17567000 W
PLW13 0.14229999 W

F2 - Processing parameters
SI 65536
SF 100.6127324 MHz
WDW EM
SSB 0
LB 0.50 Hz
GB 0
PC 1.40
